# ZNFX1 promotes AMPK-mediated autophagy against *Mycobacterium tuberculosis* by stabilizing *Prkaa2* mRNA

**DOI:** 10.1172/jci.insight.171850

**Published:** 2024-01-09

**Authors:** Honglin Liu, Zhenyu Han, Liru Chen, Jing Zhang, Zhanqing Zhang, Yaoxin Chen, Feichang Liu, Ke Wang, Jieyu Liu, Na Sai, Xinying Zhou, Chaoying Zhou, Shengfeng Hu, Qian Wen, Li Ma

**Affiliations:** Institute of Molecular Immunology, School of Laboratory Medicine and Biotechnology, Southern Medical University, Guangzhou, China.

**Keywords:** Immunology, Infectious disease, Autophagy, RNA processing, Tuberculosis

## Abstract

Tuberculosis has the highest mortality rate worldwide for a chronic infectious disease caused by a single pathogen. RNA-binding proteins (RBPs) are involved in autophagy — a key defense mechanism against *Mycobacterium tuberculosis* (*M*. *tuberculosis*) infection — by modulating RNA stability and forming intricate regulatory networks. However, the functions of host RBPs during *M*. *tuberculosis* infection remain relatively unexplored. Zinc finger NFX1-type containing 1 (ZNFX1), a conserved RBP critically involved in immune deficiency diseases and mycobacterial infections, is significantly upregulated in *M*. *tuberculosis*–infected macrophages. Here, we aimed to explore the immunoregulatory functions of ZNFX1 during *M*. *tuberculosis* infection. We observed that *Znfx1* knockout markedly compromised the multifaceted immune responses mediated by macrophages. This compromise resulted in reduced phagocytosis, suppressed macrophage activation, increased *M*. *tuberculosis* burden, progressive lung tissue injury, and chronic inflammation in *M*. *tuberculosis*–infected mice. Mechanistic investigations revealed that the absence of ZNFX1 inhibited autophagy, consequently mediating immune suppression. ZNFX1 critically maintained AMPK-regulated autophagic flux by stabilizing protein kinase AMP-activated catalytic subunit alpha 2 mRNA, which encodes a key catalytic α subunit of AMPK, through its zinc finger region. This process contributed to *M*. *tuberculosis* growth suppression. These findings reveal a function of ZNFX1 in establishing anti–*M*. *tuberculosis* immune responses, enhancing our understanding of the roles of RBPs in tuberculosis immunity and providing a promising approach to bolster antituberculosis immunotherapy.

## Abstract 2

Znfx1 functions as an RNA-binding protein to stabilize the key activating subunit of *Ampk* mRNA to maintain autophagy against *Mycobacterium tuberculosis*.

## Graphical abstract

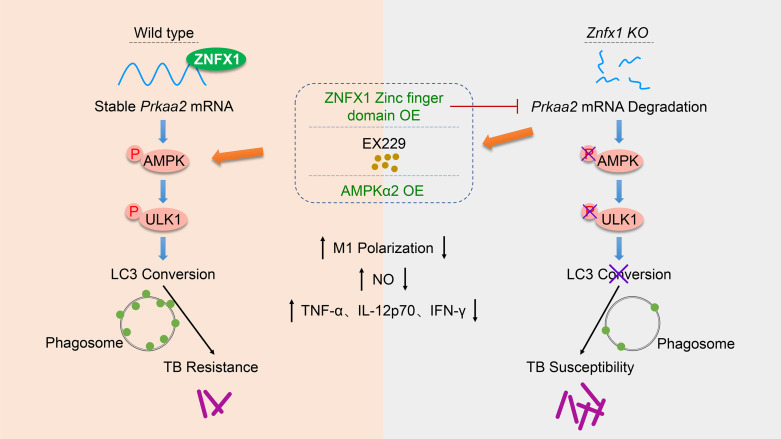

## Introduction

While the COVID-19 pandemic continues, tuberculosis (TB) remains the deadliest single-pathogen infectious disease (1). To combat the TB pathogen, *Mycobacterium tuberculosis* (*M*. *tuberculosis*), the host immune system employs multiple strategies, including the expression of cytokine and antimicrobial peptides, the production of ROS, apoptosis, and autophagy (2). However, over thousands of years of interactions with the host, *M*. *tuberculosis* has developed an arsenal of mechanisms to compromise host immune responses. Understanding the intricate details of this interaction between *M*. *tuberculosis* and the host holds substantial therapeutic potential for TB prevention and treatment.

Among the various mechanisms of immune cells against *M*. *tuberculosis*, autophagy has garnered special attention. Autophagy serves as a direct bactericidal mechanism of macrophages through the fusion of the *M*. *tuberculosis*–containing phagosome with a lysosome. Additionally, it regulates the antigen presentation activity of macrophages against *M*. *tuberculosis* (3). For instance, the treatment of *M*. *tuberculosis*–infected human monocyte-derived macrophages (hMDMs) with adenosine triphosphate (ATP) stimulates autophagy by inducing phagolysosomal fusion, resulting in a decrease in intracellular mycobacterial viability (4). The fusion between phagosomes and lysosomes is enhanced when extracellular proteins are phagocytosed and microtubule-associated protein light chain 3-II (LC3-II) is recruited to phagosome membranes. This facilitates the recruitment of MHC-II molecules to phagolysosomes, enabling them to load antigenic peptides. Consequently, this leads to the upregulation of MHC-II on the surface of antigen-presenting cells, stimulating CD4^+^ Th cells (5). Activation of autophagy necessitates the formation and activation of the unc-51 like kinase 1 (ULK1) kinase complex, which is tightly regulated, with AMPK as an activator and mTOR as an inhibitor (6). AMPK activation is crucial for establishing an immune response against *M*. *tuberculosis* infection. The AMPK signaling complex comprises 2 catalytic α, 2 β, and 3 γ regulatory subunits. Classical 5′-AMP binding is necessary for AMPK activation, serving as an energy-sensing mechanism. However, nonclassical mechanisms to activate AMPK include the binding of other ligands at the allosteric drug and metabolite site on the α subunit as well as Ca^2+^ signaling, DNA damage, and glucose starvation (7). Given that the function of AMPK depends on its coordinated subunits, maintaining appropriate expression levels of these subunits is critical for sustaining AMPK activity against *M*. *tuberculosis*.

Intracellular protein levels are regulated at various stages, including gene transcription, posttranscription, and translation. Posttranscriptional regulation determines the fate of mRNAs and noncoding RNAs, including RNA stability, transport, splicing, modification, and translation, which is tightly associated with RNA-binding proteins (RBPs) (8, 9). RBPs interact stably or transiently with RNAs, playing a role in processing and regulating virtually all RNAs. Through concise, albeit not fully elucidated, mechanisms, RBPs spatially and temporally regulate mRNA expression to orchestrate various cellular processes, including autophagy (10). For example, in neuroblastoma, the neuronal origin HuD has been found to dampen mTOR complex 1 activity and promote autophagy by stabilizing mRNAs coding for growth factor receptor bound protein 10 and ADP ribosylation factor like GTPase 6 interacting protein 1. This contributes to the suppression of apoptosis and consequently sustains tumor growth (11). Pumilio RNA binding family member 2, induced upon aging, functions in inhibiting the translation of the mRNA encoding the mitochondrial fission factor, which impairs mitochondrial fission and mitophagy, thereby mediating age-related mitochondrial dysfunctions (12). Additionally, the efficiency of autophagy relies on the regulation of mRNA stability. As a central component in autophagy, LC3-II has been demonstrated to be an RBP that directly binds to mRNAs. This association is promoted by autophagic activation and triggers the rapid degradation of target mRNAs before autolysosome formation. Through this mechanism, LC3-II rapidly degrades protein arginine methyltransferase 1 mRNA, which encodes a negative regulator of autophagy, subsequently facilitating autophagy (13).

Zinc finger proteins (ZFPs) are classical RBPs, comprising approximately 30 types based on the zinc finger domain structure as approved by the Human Genome Organisation Gene Nomenclature Committee (14). ZFPs consist of 1 α-helix and 2 antiparallel β-folding regions, which spontaneously fold into a finger-like structure. This configuration includes a pair of cysteine residues (C) at the N-terminal and a pair of histidine residues (H) at the C-terminal of the zinc finger, creating the classical C2H2 domain structure of ZFPs. These 4 residues create a cavity that accommodates 1 zinc ion, forming a tetrahedral structure. Due to their structural characteristics, ZFPs can selectively bind to specific target structures, thereby regulating gene expression, cell differentiation, embryonic development, and pathogenesis, including TB (15, 16). For instance, the transcriptional repressor protein zinc finger and BTB domain 25 (ZBTB25), along with corepressor Sin3a, associate with histone deacetylase 1 (HDAC1) to form a silencing complex on the promoter. This complex downregulates *IL-12B* in *M*. *tuberculosis*–infected macrophages. Inhibition of HDAC1 and ZBTB25 enhances autophagy, activates JAK2 and STAT4, and promotes the killing of intracellular pathogens (17). However, owing to the large number of existing ZFPs, their roles in regulating host immune response against *M*. *tuberculosis* remain to be elucidated.

In this study, we aimed to identify differentially expressed host ZFPs that are critically involved in the response against *M*. *tuberculosis* infection and found the zinc finger NFX1-type containing 1 (ZNFX1), a conserved member of the ZFP family, was significantly upregulated in mouse bone marrow–derived macrophages (BMDMs) in response to *M*. *tuberculosis* infection, suggesting its involvement in TB development. Furthermore, we intended to explore the function and mechanism of ZNFX1 in *M*. *tuberculosis* infection. Our research aids in deepening the understanding of the immune mechanisms underlying TB and provides a potential approach for anti-TB immunotherapy.

## Results

### M. tuberculosis–inducible ZNFX1 contributes to the immune response against M. tuberculosis.

**Figure 1:**
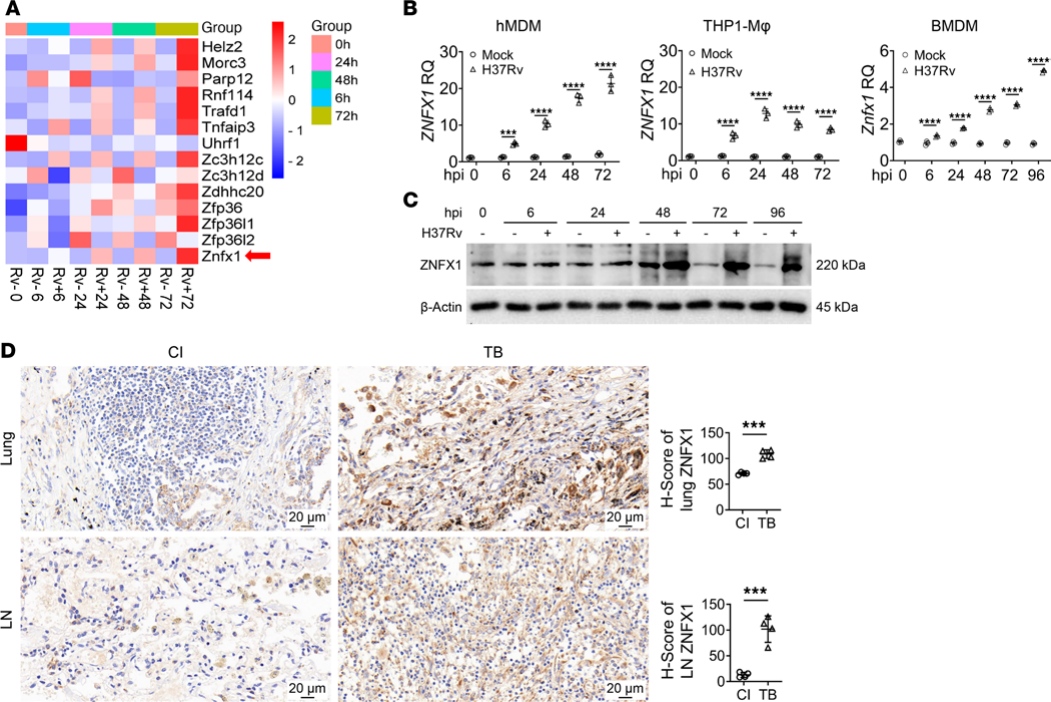
Upregulation of ZNFX1 following *M*. *tuberculosis* infection. (**A**) Heatmap of expression data of several ZFPs identified using quantitative real-time PCR (qPCR). (**B**) Quantification of mRNA coding for ZNFX1 in different macrophages following H37Rv infection (MOI = 2), using qPCR. hpi, hours postinfection; RQ, relative quantification. (**C**) Western blot analysis of ZNFX1 expression in H37Rv-infected BMDMs. (**D**) Immunohistochemistry detection and statistical analysis of ZNFX1 expression in the lung tissues and LNs of patients with CI or TB (*n* = 4). A 2-way ANOVA with Holm-Šídák post hoc test (**B**) or an unpaired *t* test (**D**) was used for statistical analysis. Data are presented as mean ± SD and are representative of at least 3 experiments with similar observations. ***P < 0.001; ****P < 0.0001.

To explore differentially expressed host ZFPs that play critical roles in response to *M*. *tuberculosis* infection, we screened bioRxiv preprinted protein data sets of BMDMs using liquid chromatography-mass spectrometry analysis (18). This screening identified approximately 50 ZFPs with significant changes in expression following *M*. *tuberculosis* infection (Supplemental Figure 1; supplemental material available online with this article; https://doi.org/10.1172/jci.insight.171850DS1). To investigate the involvement of these ZFPs in the immune response against *M*. *tuberculosis* infection, we examined their expression over time (0, 6, 24, 48, and 72 hours) in macrophages following infection with the *M*. *tuberculosis* virulent strain H37Rv, comparing it with mock infection. Among the differentially expressed ZFPs in response to *M*. *tuberculosis* infection (Figure 1A), ZNFX1 stood out because of the known genetic association of its human homolog with Mendelian susceptibility to mycobacterial disease (MSMD) (19). This suggested a potential role for ZNFX1 in the development of *M*. *tuberculosis*–induced TB. We validated the upregulation of ZNFX1 following H37Rv infection in both human and murine macrophages (Figure 1, B and C; see complete unedited blots in the supplemental material). To extend these bioinformatic findings to clinical relevance, we examined ZNFX1 expression in clinical samples. We observed that ZNFX1 expression was significantly elevated in the lung tissues and lymph nodes (LNs) of patients with TB compared with those tissues in patients with non-TB chronic inflammation (CI). This observation highlighted a correlation between ZNFX1 and TB pathogenesis (Figure 1D). Consequently, ZNFX1 was selected for further analysis.

**Figure 2:**
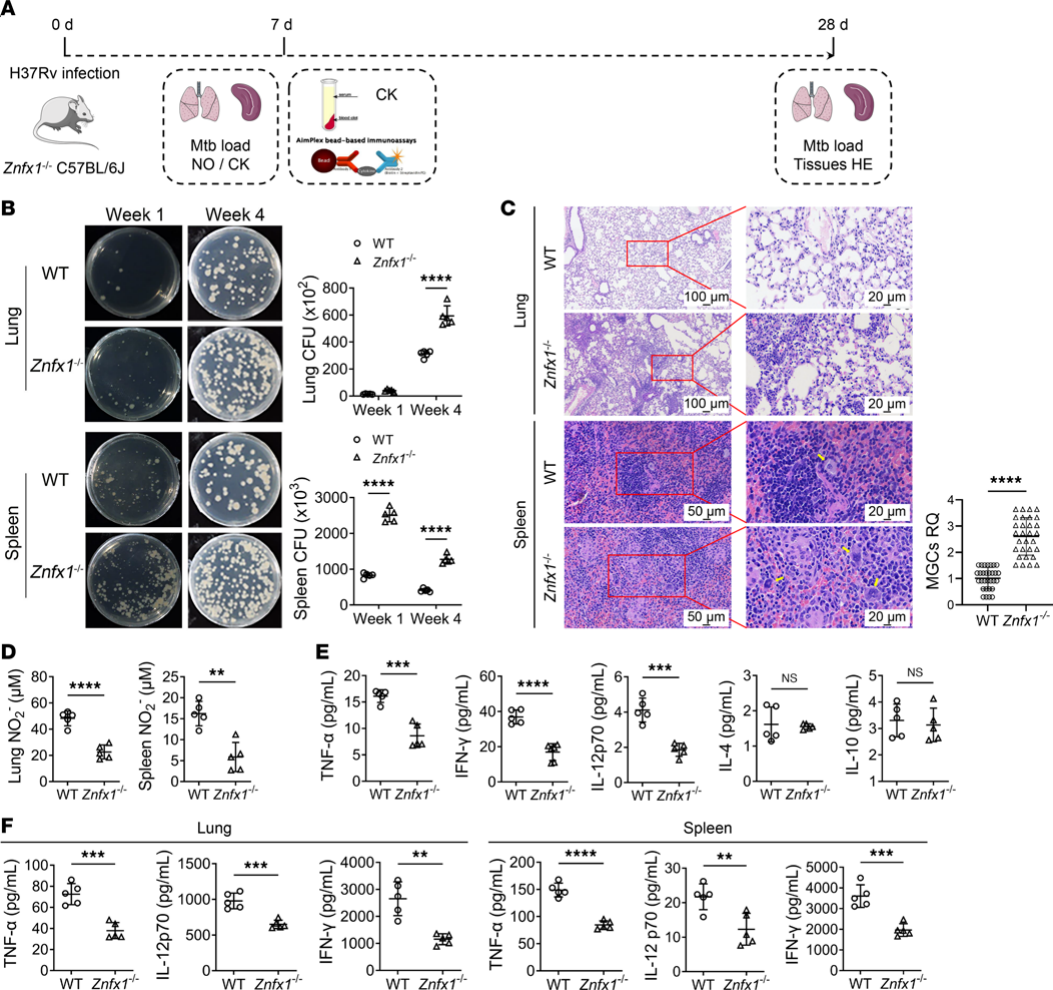
In vivo immune response of *Znfx1*^–/–^ mice infected with H37Rv. (**A**) Schematic diagram of the time points of assays during the in vivo evaluation of *Znfx1*^–/–^-induced immune responses against *M*. *tuberculosis* infection. CK, cytokine. (**B**) In vivo *M*. *tuberculosis* load in the lung and spleen tissues of *Znfx1*^–/–^ mice at 1 and 4 weeks after H37Rv infection (*n* = 5). (**C**) H&E staining of the lung and spleen tissues of *Znfx1*^–/–^ mice. The splenic MGCs were quantified (*n* = 5, with 30 randomly selected fields of view for statistics). Yellow arrows indicate MGCs. (**D**) Measurement of NO production indicated as the concentration of NO_2_^–^ in the lung and spleen tissues 1 week postinfection (*n* = 5). (**E**) Luminex multiplex assays of cytokine expression in the peripheral blood of mice 1 week after H37Rv infection (*n* = 5). (**F**) ELISA of cytokine expression in the lung and spleen of mice 1 week after H37Rv infection (*n* = 5). A 2-way ANOVA with Holm-Šídák post hoc test (**B**) or an unpaired 2-tailed *t* test was used (**C**–**F**) was used for statistical analysis. Data are presented as mean ± SD and are representative of at least 3 experiments with similar observations. **P < 0.01; ***P < 0.001; ****P < 0.0001.

Human and murine ZNFX1 proteins share high sequence similarity (87.64%), possess similar function domains (Supplemental Figure 2A), and serve identical functions (20). To investigate the roles of ZNFX1 during *M*. *tuberculosis* infection, we infected *Znfx1*-knockout (*Znfx1*^–/–^) mice (Supplemental Figure 2, B and C) with H37Rv (Figure 2A). Bacterial load assessments in vivo revealed significantly higher levels of *M*. *tuberculosis* in *Znfx1*^–/–^ mice than in WT mice, accompanied by an inverse trend of *M*. *tuberculosis* growth in the lung and spleen tissues (Figure 2B). Histological examination revealed that *Znfx1* knockout resulted in marked tissue damage in the lungs, accompanied by inflammatory infiltration in both the lung and spleen tissues. Moreover, we observed a significant increase in splenic multinucleated giant cells (MGCs), which is indicative of chronic inflammation (21) (Figure 2C).

Consistent with the elevated in vivo *M*. *tuberculosis* load, *Znfx1*^–/–^ mice exhibited suppressed production of the bactericidal NO (Figure 2D). Furthermore, *Znfx1* knockout inhibited the expression of TNF-α, IL-12p70, and IFN-γ, which are cytokines associated with T cell activity and macrophage activation by T cells during *M*. *tuberculosis* infection. This effect was observed both in the peripheral blood (Figure 2E) and in the lung and spleen tissues 1 week after infection (Figure 2F). However, ZNFX1 did not appear to affect IL-4 and IL-10 expression in the early stages of infection (Figure 2E). Collectively, these observations suggest that ZNFX1 plays a role in promoting the anti–*M*. *tuberculosis* immune response.

### ZNFX1 regulates macrophage activity against M. tuberculosis infection.

**Figure 3:**
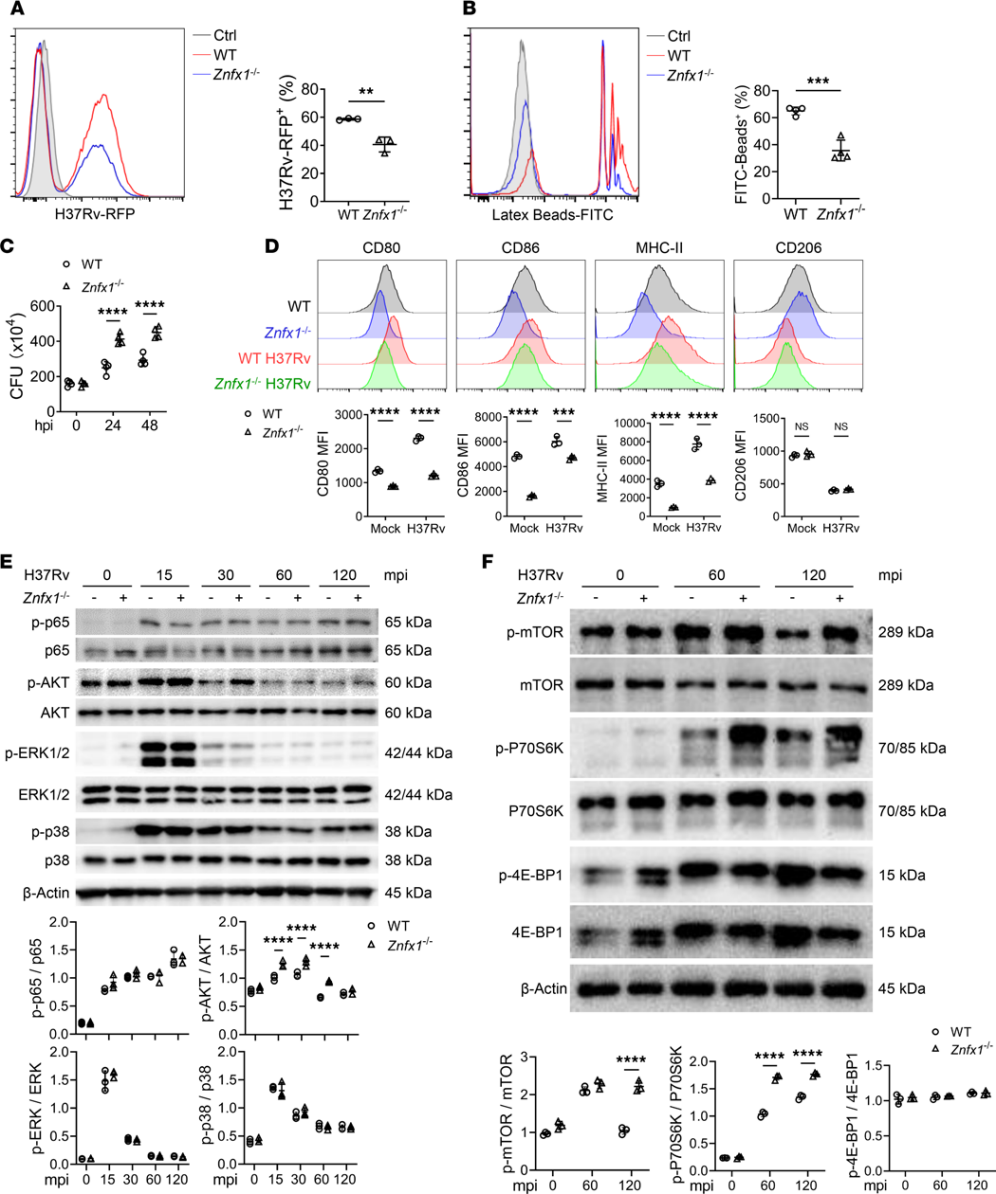
Regulation of macrophage activity by ZNFX1 and exploration of possible signaling mechanisms. (**A**) Flow cytometry analysis of red fluorescence–positive WT and *Znfx1*^–/–^ BMDMs infected with H37Rv carrying the red fluorescence protein (RFP) gene (i.e., H37Rv-RFP) at MOI = 10 at 2 hpi (*n* = 3). (**B**) Flow cytometry analysis of green fluorescence–positive WT and *Znfx1*^–/–^ BMDMs incubated with FITC-conjugated latex beads (*n* = 4). (**C**) CFU assays of intracellular *M*. *tuberculosis* levels in H37Rv-infected WT and *Znfx1*^–/–^ BMDMs at MOI = 5 (*n* = 4). (**D**) Flow cytometry analysis of CD80, CD86, MHC-II, and CD206 expression on the surface of H37Rv-infected WT and *Znfx1*^–/–^ BMDMs at MOI = 2 at 24 hpi (*n* = 3). (**E**) Western blot assay of the regulatory effects of ZNFX1 on activation of signaling pathways following *M*. *tuberculosis* infection at MOI = 5. p-, phosphorylated. (**F**) Western blot assay of the regulatory effects of ZNFX1 on activation of the autophagy-associated mTOR signaling pathways. An unpaired 2-tailed *t* test (**A** and **B**) or a 2-way ANOVA with Holm-Šídák post hoc test (**C**–**F**) was used for statistical analysis. Data are presented as mean ± SD and are representative of at least 3 experiments with similar observations. **P < 0.01; ***P < 0.001; ****P < 0.0001.

Considering the involvement of ZNFX1 in monocytosis (19), we initially investigated the population of macrophages, the first line of defense against *M*. *tuberculosis* infection (22), in the lungs, spleens, and LNs in *Znfx1*^–/–^ mice. However, the results of flow cytometry analysis did not reveal any significant differences (Supplemental Figure 3A). Consistent with its in vivo immune-suppressive effects, *Znfx1*^–/–^ BMDMs exhibited reduced phagocytic activity when challenged with H37Rv labeled with a red fluorescent protein (H37Rv-RFP; Figure 3A). This reduced phagocytic activity was also evident when cells were incubated with FITC-latex beads (Figure 3B and Supplemental Figure 3B). Moreover, *Znfx1* knockout significantly enhanced intracellular *M*. *tuberculosis* multiplication (Figure 3C). *Znfx1*^–/–^ BMDMs expressed significantly reduced levels of CD80, CD86, and MHC-II in response to H37Rv infection, without influencing the expression of CD206, a marker of type II macrophages (Figure 3D). However, compared with WT BMDMs, H37Rv-infected *Znfx1*^–/–^ BMDMs did not significantly affect the expression of IL-1β, IL-6, or TNF-α but suppressed IL-12p35 expression (Supplemental Figure 3C). Collectively, these results indicate that ZNFX1 directly modulates the immune activities of macrophages during responses to *M*. *tuberculosis*, possibly in a cytokine-independent manner.

### ZNFX1 regulates anti–M. tuberculosis immune responses in macrophages through autophagy.

As an interferon-stimulated gene (ISG), ZNFX1 plays a crucial role in regulating the IFN signaling pathway and innate immune response by reducing the half-life of ISG mRNA. The absence of ZNFX1 was expected to lead to increased ISG expression and cellular response to IFN (23). Additionally, ZNFX1 serves as a sensor for double-stranded nucleic acids during viral infection (20). To determine whether ZNFX1 functions similarly in *M*. *tuberculosis*–infected macrophages, we stimulated *Znfx1*^–/–^ BMDMs with H37Rv, LPS, and vesicular stomatitis virus (VSV) and assessed the expression levels of ISGs. Similar to previous reports, the expression levels of IFIT1, IRF1, and MX1 in *Znfx1*^–/–^ BMDMs were higher than those in WT BMDMs following H37Rv infection, despite the increased intracellular *M*. *tuberculosis* burden in *Znfx1*^–/–^ BMDMs (Supplemental Figure 4A). However, no significant difference in responses to LPS and VSV treatments was observed between *Znfx1*^–/–^ and WT BMDMs, ruling out the possibility of ZNFX1 functioning as a dsRNA sensor in BMDMs (Supplemental Figure 4A). Furthermore, knockout of *Znfx1* did not affect the activation of STAT1 in H37Rv-infected BMDMs (Supplemental Figure 4B). Moreover, treatment with IFN-γ similarly promoted phagocytosis and intracellular suppression of H37Rv survival in both WT and *Znfx1*^–/–^ BMDMs, indicating that *Znfx1* knockout did not influence the response to IFN-γ in BMDMs (Supplemental Figure 4C).

**Figure 4:**
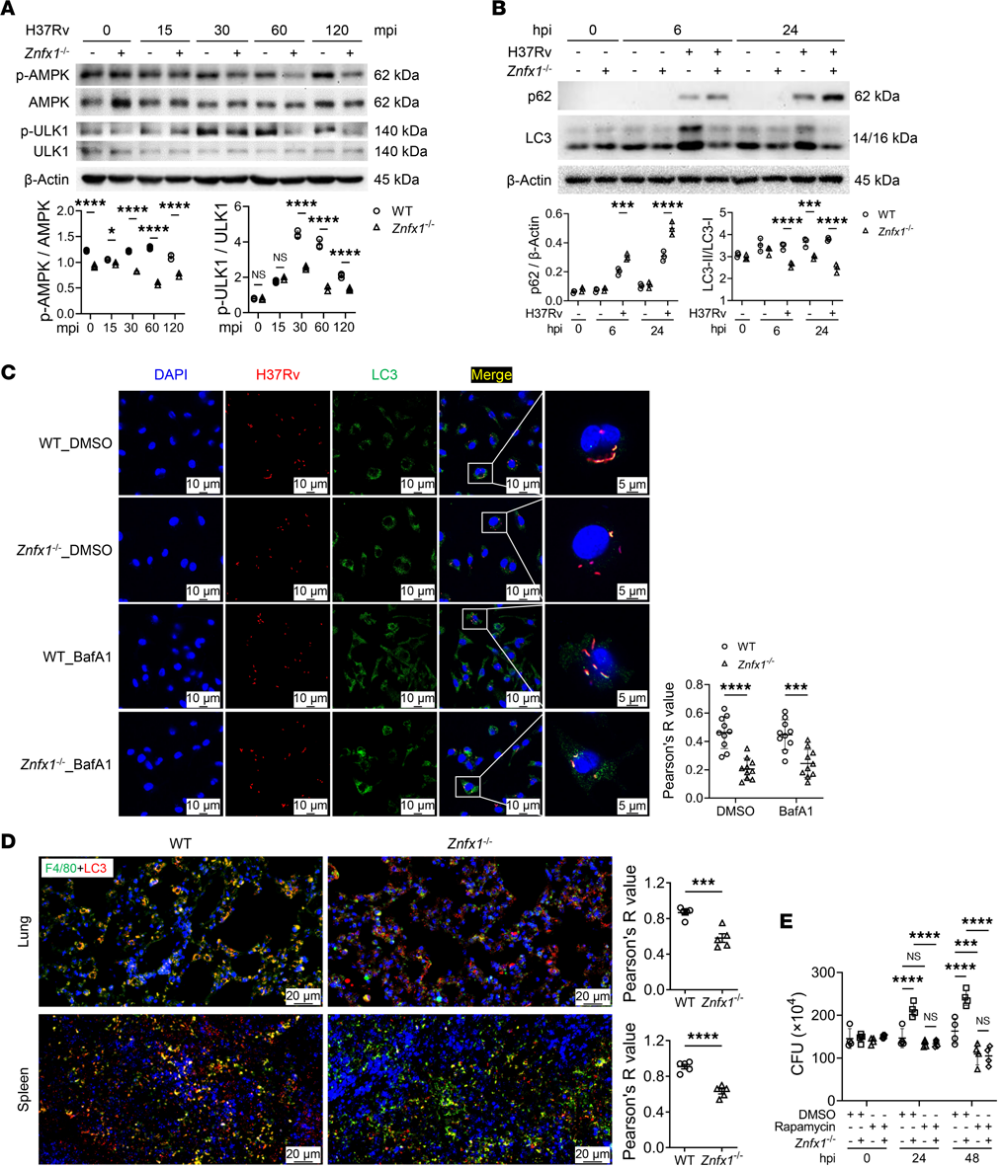
ZNFX1 regulation of macrophage bactericidal activity through the autophagy signaling pathway. (**A**) Western blot assay of the regulatory effects of ZNFX1 on AMPK activation. mpi, minutes postinfection. (**B**) Western blot assay of the regulatory effects of ZNFX1 on the levels of p62 and LC3-I/II conversion. (**C**) Immunofluorescence assays of LC3 puncta in *Znfx1*^–/–^ BMDMs treated with BafA1, followed by infection with H37Rv-RFP (*n* = 3, with 10 randomly selected fields of view for statistics). (**D**) Double-staining immunofluorescence assays of LC3 in F4/80^+^ macrophages in the lung and spleen tissues of WT and *Znfx1*^–/–^ mice following H37Rv infection (*n* = 5, with 5 randomly selected fields of view for statistics). “Pearson’s R value” refers to Pearson’s correlation coefficient. (**E**) CFU assays of *M*. *tuberculosis* load in WT and *Znfx1*^–/–^ BMDMs treated with rapamycin (*n* = 5). A 2-way ANOVA with Holm-Šídák post hoc test (**A**–**D**) was used for statistical analysis. Data are presented as mean ± SD and are representative of at least 3 experiments with similar observations. *P < 0.05; ***P < 0.001; ****P < 0.0001.

To explore the potential functional mechanism of ZNFX1, we evaluated several classical signaling pathways previously reported to be activated during *M*. *tuberculosis* infection (24). Among these pathways, only the activation status of AKT was altered in *Znfx1*^–/–^ BMDMs (Figure 3E). AKT is a multifaceted modulator known to regulate cytokine induction, DNA damage response, and autophagy (25). Autophagy is a well-characterized bactericidal mechanism during *M*. *tuberculosis* infection (26). Accordingly, the activation of the mTOR pathway, a direct downstream target of AKT that suppresses autophagy, was significantly enhanced in *Znfx1*^–/–^ BMDMs (Figure 3F). Conversely, the activation of AMPK and ULK1 was significantly compromised in *Znfx1*^–/–^ BMDMs compared with that in WT BMDMs (Figure 4A). Autophagy was markedly repressed in *Znfx1*^–/–^ BMDMs, as indicated by the accumulation of p62 and the suppression of LC3-I/II conversion (Figure 4B). Furthermore, *Znfx1* knockout reduced LC3-I/II puncta in H37Rv-RFP–containing vacuoles compared with WT BMDMs, even in the presence of bafilomycin A1 (BafA1), an inhibitor of V-ATPase (Figure 4C). These findings suggest that ZNFX1 regulates the anti-infective activity of macrophages through autophagy, which was further validated by reduced LC3-I/II puncta in F4/80^+^ macrophages in the lung and spleen tissues of H37Rv-infected *Znfx1*^–/–^ mice (Figure 4D). Moreover, treatment with rapamycin, an autophagy agonist, significantly inhibited intracellular *M*. *tuberculosis* growth in WT BMDMs at 48 hpi and reversed the enhanced intracellular *M*. *tuberculosis* proliferation in *Znfx1*^–/–^ mice at both 24 and 48 hpi (Figure 4E).

### AMPKα2 mediates the role of ZNFX1 in macrophage anti–M. tuberculosis immune responses.

**Figure 5:**
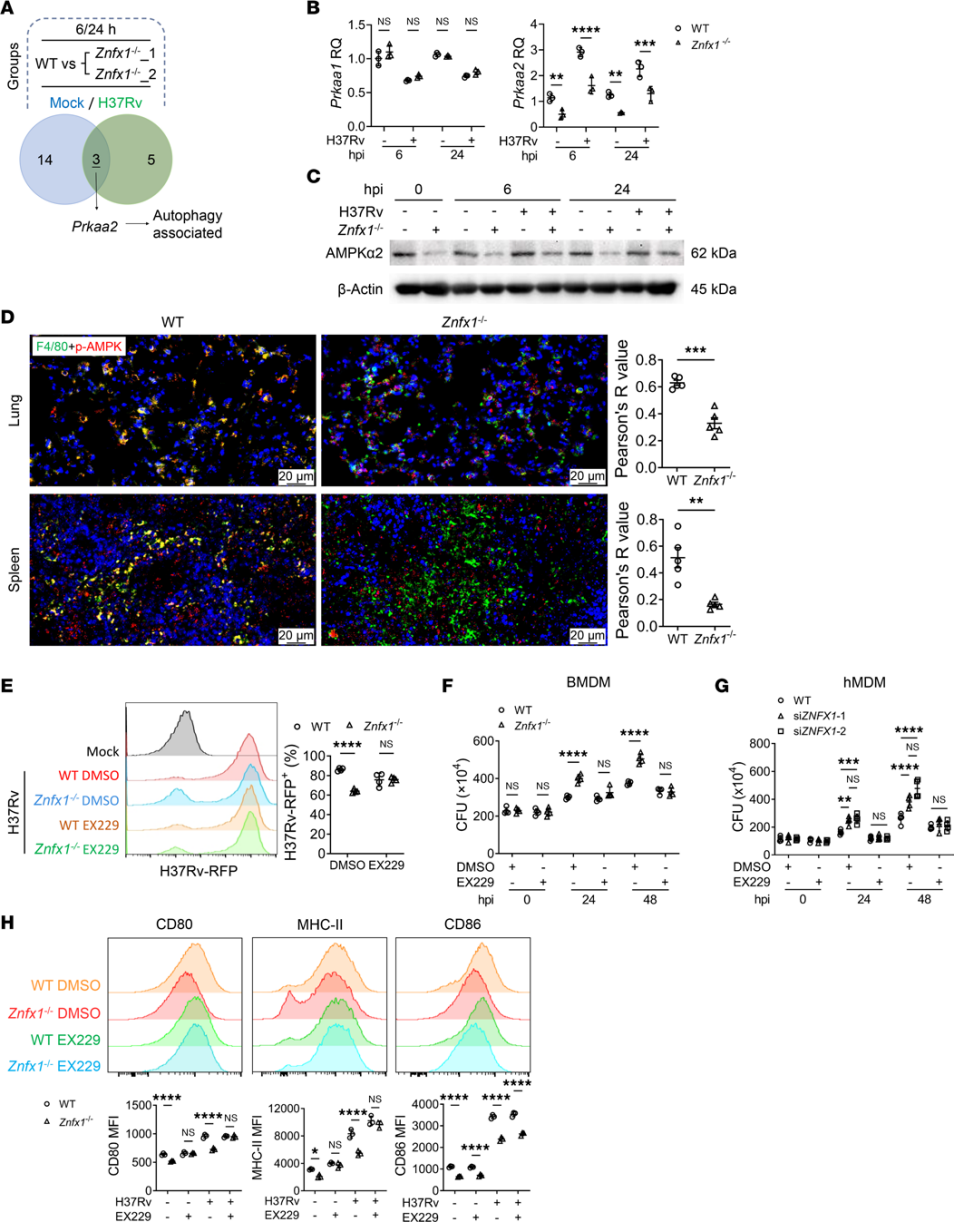
AMPKα2-mediated regulatory effects of ZNFX1 on autophagy and macrophage activity. (**A**) High-throughput RNA sequencing analysis of H37Rv-infected (MOI = 2) WT and *Znfx1*^–/–^ BMDMs at 6 and 24 hpi identified *Prkaa2* as the downstream target of ZNFX1. (**B** and **C**) Expression levels of *Prkaa2* and its coded protein AMPKα2 following H37Rv (MOI = 2) in WT and *Znfx1*^–/–^ BMDMs, using qPCR (**B**) and Western blotting (**C**), respectively. (**D**) Double-staining immunofluorescence assays of p-AMPK in F4/80^+^ macrophages in the lung and spleen tissues of WT and *Znfx1*^–/–^ mice following H37Rv infection (*n* = 5, with 5 randomly selected fields of view for statistics). “Pearson’s R value” refers to Pearson’s correlation coefficient. (**E**) Flow cytometry analysis of red fluorescence–positive WT and *Znfx1*^–/–^ BMDMs treated with EX229, followed by infection with H37Rv-RFP (MOI = 10, *n* = 4). (**F** and **G**) CFU assays of intracellular *M*. *tuberculosis* levels in H37Rv-infected (MOI = 5) WT and *Znfx1*^–/–^ BMDMs (**F**) and *ZNFX1*-silenced hMDMs (**G**) following EX229 treatment (*n* = 4). si, siRNA. (**H**) Flow cytometry analysis of CD80, CD86, and MHC-II expression on the surface of WT and *Znfx1*^–/–^ BMDMs treated with EX229 and infected with H37Rv (MOI = 2, *n* = 3). A 2-way ANOVA with Holm-Šídák post hoc test (**B** and **E**–**H**) or an unpaired 2-tailed *t* test (**D**) was used for statistical analysis. Data are presented as mean ± SD and are representative of at least 3 experiments with similar observations. **P < 0.01; ***P < 0.001; ****P < 0.0001.

To investigate the regulatory mechanism of ZNFX1 on autophagy, we conducted high-throughput RNA sequencing on *Znfx1*^–/–^ BMDMs infected with H37Rv. Using criteria log_2_ fold-change (log_2_FC) > 1 and *P* < 0.05, we performed serial Venn analysis and identified 3 genes (protein kinase AMP-activated catalytic subunit alpha 2 [*Prkaa2*], *Mmp9*, and *Gdf3*) with significantly differential expression in *Znfx1*^–/–^ BMDMs compared with in WT BMDMs at both 6 and 24 hpi, regardless of H37Rv infection (Supplemental Table 3). Among these 3 genes, only *Prkaa2* directly participated in autophagy by encoding the critical component of the AMPK signaling pathway — AMPKα2 (Figure 5A; National Center for Biotechnology Information [NCBI] Sequence Read Archive data: PRJNA929691). qPCR and Western blot analysis validated that the absence of ZNFX1 led to a significant downregulation of *Prkaa2*, irrespective of H37Rv infection (Figure 5, B and C). In contrast, the expression of another component of AMPKα, *Prkaa1*, remained unresponsive to Z*nfx1* knockout (Figure 5, B and C). Additionally, the levels of p-AMPK in F4/80^+^ macrophages in the lung and spleen tissues of *Znfx1*^–/–^ mice were significantly lower than those in WT mice (Figure 5D). The expression of *Prkaa2* increased following H37Rv infection (Supplemental Figure 5A), indicating its close association with the response to *M*. *tuberculosis* infection and implicating AMPKα2 in mediating the functions of ZNFX1. To test this hypothesis, we used EX229, an AMPK stimulator, to treat H37Rv-infected *Znfx1*^–/–^ BMDMs, following confirmation of its stimulative activity (Supplemental Figure 5B). Similarly, we used EX229 to treat *ZNFX1*-silenced hMDMs, after validating the silencing efficiency (Supplemental Figure 5C). EX229 treatment restored the formation of LC3-I/II puncta in *Znfx1*^–/–^ BMDMs (Supplemental Figure 5D), along with AMPK and ULK1 activation (Supplemental Figure 5E), enhanced LC3-I/II conversion, and reduced p62 levels (Supplemental Figure 5F). Similar results were observed in *ZNFX1*-silenced hMDMs (Supplemental Figure 5, G and H). Activation of AMPKα2 increased the phagocytosis of *M*. *tuberculosis* by *Znfx1*^–/–^ BMDMs (Figure 5E) and inhibited intracellular *M*. *tuberculosis* survival in both *Znfx1*^–/–^ BMDMs and *ZNFX1*-silenced hMDMs, reversing the pro–*M*. *tuberculosis* effects induced by lack of ZNFX1 (Figure 5, F and G). Overexpression of *Prkaa2* in *Znfx1*^–/–^ BMDMs also promoted the formation of LC3-I/II puncta (Supplemental Figure 6, A and B) and enhanced the bactericidal activity against intracellular *M*. *tuberculosis* (Supplemental Figure 6C). These findings indicate a crucial role of ZNFX1 in autophagy and bactericidal activity against *M*. *tuberculosis* in BMDMs, which is mediated by AMPKα2.

**Figure 6:**
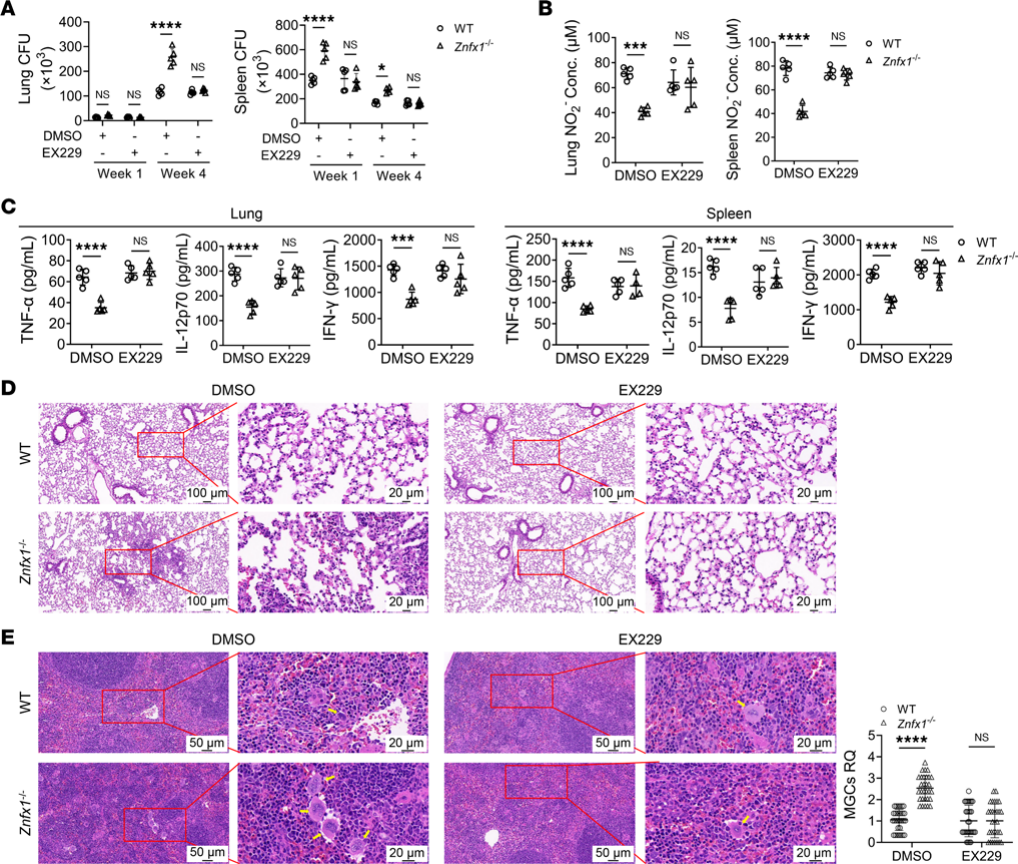
Recovery of the anti–*M*. *tuberculosis* immune response through activation of AMPK signaling resulting from *Znfx1* knockout. WT and *Znfx1*^–/–^ mice were treated with EX229 and infected with H37Rv. (**A**) CFU assays of *M*. *tuberculosis* load in the lung and spleen (*n* = 5). (**B**) ELISA of cytokine expression in the lung and spleen of mice 1 week after H37Rv infection (*n* = 5). (**C**) Assays of NO production in the lung and spleen 1 week postinfection (*n* = 5). (**D** and **E**) H&E staining of the lung and spleen. The MGCs in the spleen were quantified. Yellow arrows indicate MGCs (*n* = 5, with 30 randomly selected fields of view for statistics). A 2-way ANOVA with Holm-Šídák post hoc test (**A**–**C** and **E**) was used for statistical analysis. Data are presented as mean ± SD and are representative of at least 3 experiments with similar observations. *P < 0.05; ***P < 0.001; ****P < 0.0001.

Considering the contribution of ZNFX1 to host immune response in vivo, we evaluated the role of AMPKα2 in vitro and in vivo. EX229 treatment restored the surface expression of CD80 and MHC-II, except CD86, in *Znfx1*^–/–^ BMDMs (Figure 5H). EX229 treatment of H37Rv-infected mice led to reduced *M*. *tuberculosis* loads in the lung and spleen tissues (Figure 6A) and restored the production of NOS as well as the protective cytokines TNF-α, IL-12p70, and IFN-γ (Figure 6, B and C) in *Znfx1*^–/–^ mice. Furthermore, AMPK activation contributed to injury repair in lung tissues, compromised inflammatory infiltration (Figure 6D), and suppressed CI, as indicated by the splenic MGCs resulting from *Znfx1* knockout after H37Rv infection (Figure 6E). Collectively, these results demonstrate that AMPK mediates the immunoregulatory activity of ZNFX1.

### ZNFX1 stabilizes Prkaa2 mRNA through its zinc finger region.

**Figure 7:**
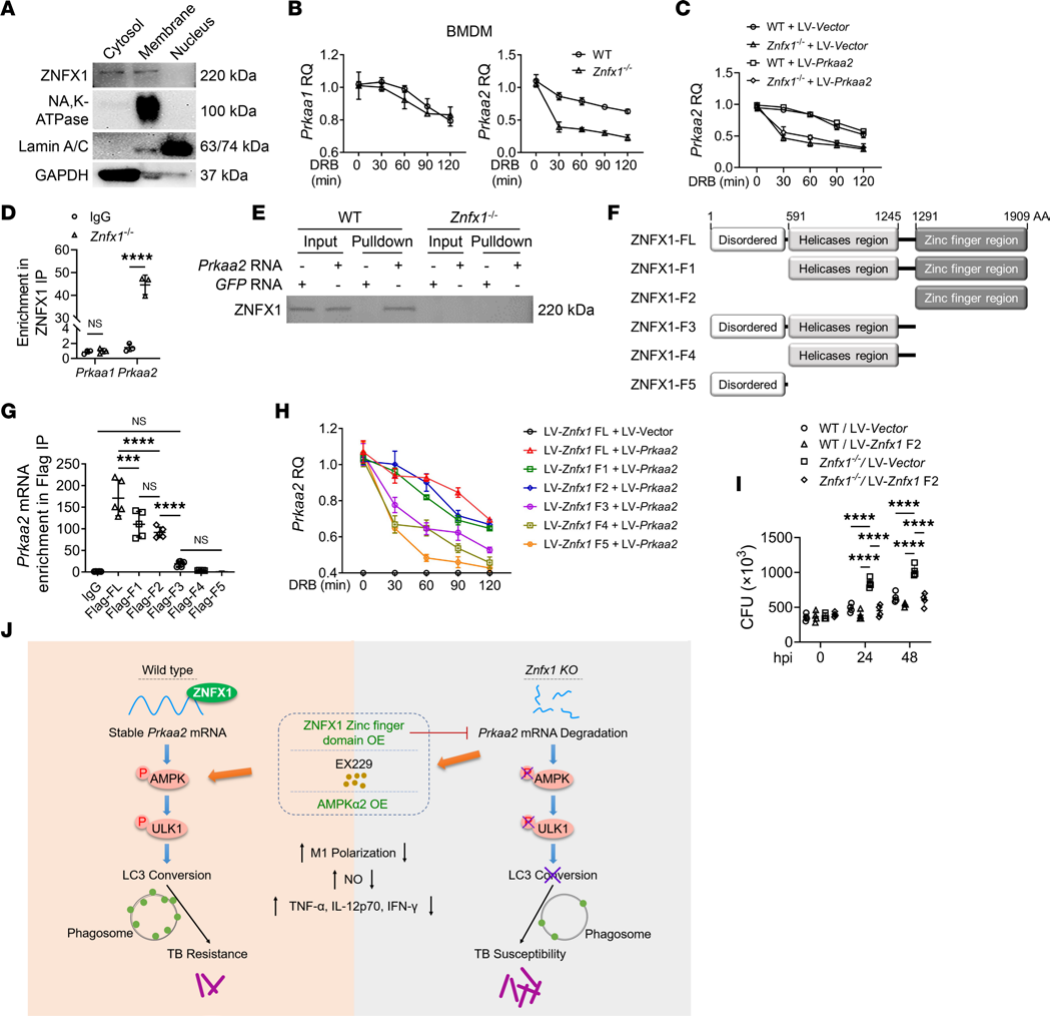
Stabilization of *Prkaa2* mRNA by ZNFX1 through interaction with its zinc finger region. (**A**) Western blotting of ZNFX1 in the cytosol, membrane, and nucleus fraction of BMDMs. (**B**) qPCR analysis of *Prkaa1* and *Prkaa2* in *Znfx1*^–/–^ BMDMs treated with DRB (*n* = 3). (**C**) qPCR analysis of *Prkaa2* in WT and *Znfx1*^–/–^ BMDMs infected with LV-*Prkaa2* (*n* = 3). LV, lentiviral. (**D**) RIP assay using anti-ZNFX1 antibody and qPCR analysis of the association between ZNFX1 protein and *Prkaa1* and *Prkaa2* mRNA in BMDMs (*n* = 3). (**E**) RNA pulldown assay using biotinylated *Prkaa2* and GFP transcripts and Western blotting of the association between *Prkaa2* mRNA and ZNFX1 protein in BMDMs. (**F**) Schematic diagram of recombinant plasmids carrying full-length or various truncated forms of the *Znfx1* gene accompanied by a FLAG tag. (**G**) RIP assay using anti-FLAG antibody and qPCR analysis of the association between full-length or truncated forms of ZNFX1 protein and *Prkaa2* mRNA in HEK293T cells transfected with various *Znfx1* expression plasmids (*n* = 5). (**H**) qPCR analysis of *Prkaa2* in HEK293T cells transfected with various *Znfx1* expression plasmids and treated with DRB (*n* = 3). (**I**) CFU assay of *M*. *tuberculosis* load in WT and *Znfx1*^–/–^ BMDMs transfected with the F2 truncated form of *Znfx1* (*n* = 4). (**J**) Schematic diagram of the molecular mechanism of ZNFX1 in the regulation of autophagy against *M*. *tuberculosis* infection. OE, overexpression. A 2-way ANOVA with Holm-Šídák post hoc test (**D** and **I**) or a 1-way ANOVA followed by multiple-comparison test (**G**) was used for statistical analysis. Data are presented as mean ± SD and are representative of at least 3 experiments with similar observations. ***P < 0.001; ****P < 0.0001.

Both enhanced activation and upregulated expression of AMPKα2 contribute to AMPKα2-mediated autophagy (27). ZFPs, as the largest protein family, localize to different intracellular locations to perform diverse functions (28). To investigate how ZNFX1 regulates AMPKα2 expression, we first identified the subcellular localization of ZNFX1. Fractionation experiments showed that ZNFX1 is primarily localized in the cytoplasm and membrane, rather than in the nucleus (Figure 7A), preliminarily excluding the possibility that ZNFX1 transcriptionally regulates AMPKα2 expression. AMPKα2 is reportedly phosphorylated by liver kinase B1 (LKB1) on the mitochondrial membrane (29), which is consistent with the membrane localization of ZNFX1 found in this study and previous reports indicating mitochondrial localization (20). We initially supposed that ZNFX1 functions as a scaffold protein to promote the association between AMPKα2 and its upstream kinase. However, co-IP detection did not support the association between ZNFX1 and AMPKα2 or LKB1 in BMDMs, regardless of H37Rv infection (Supplemental Figure 7A). This observation excluded the potential scaffold role of ZNFX1 in promoting AMPKα2 phosphorylation by LKB1.

Thereafter, we explored the possibility that ZNFX1 might regulate AMPKα2 expression levels. Treatments with the protein translation inhibitor cycloheximide (CHX) were applied to H37Rv-infected *Znfx1*^–/–^ BMDMs. We observed a marked reduction in AMPKα2 and total AMPKα protein levels in both *Znfx1*^–/–^ and WT BMDMs following CHX treatment, with similar degradation rates. This observation indicated that ZNFX1 was not involved in AMPKα protein stabilization (Supplemental Figure 7B). Considering a previous report that ZNFX1 modulated the half-life of mRNAs coding for ISGs at the posttranscriptional level (23), we treated *Znfx1*^–/–^ BMDMs with 6-dichlorobenzimidazole 1-β-D-ribofuranoside (DRB), an inhibitor of RNA polymerase II for ceasing transcription elongation. Subsequently, the mRNA levels of *Prkaa1* and *Prkaa2* were compared. *Prkaa2* mRNA, but not *Prkaa1* mRNA, decayed remarkably faster in *Znfx1*^–/–^ BMDMs than in WT BMDMs following DRB treatment (Figure 7B). Similar results were observed in ZNFX1-silenced hMDMs (Supplemental Figure 7C), suggesting that ZNFX1 plays a key role in stabilizing the level of *Prkaa2* mRNA. The effect of ZNFX1 could be observed even for ectopically expressed *Prkaa2* mRNA. In the presence of DRB, the degradation rate of *Prkaa2* mRNA was substantially faster in *Znfx1*^–/–^ BMDMs than in WT BMDMs (Figure 7C). Furthermore, the specific association between ZNFX1 and *Prkaa2* mRNA, but not *Prkaa1* mRNA, was validated using RNA immunoprecipitation (RIP) assay (Figure 7D). The RNA pulldown experiment indicated that *Prkaa2* mRNA could readily combine with the endogenous ZNFX1 protein of BMDMs (Figure 7E). Comparing the stabilizing capacity of ZNFX1 for *Prkaa2* mRNA, but not *Prkaa1* mRNA, with that of ISGs observed in human primary fibroblasts (23) emphasized the interacting specificity and efficacy between ZNFX1 protein and *Prkaa2* mRNA. These results strongly support that ZNFX1 functions in maintaining the stability of *Prkaa2* mRNA, consistent with the transcriptomic profile.

To determine the binding domain of ZNFX1 with *Prkaa2* mRNA, we expressed truncated ZNFX1 proteins tagged with FLAG peptide in HEK293T cells (Figure 7F and Supplemental Figure 7D). RIP assays indicated that the zinc finger domain of ZNFX1 was the primary region responsible for binding and stabilizing *Prkaa2* mRNA, whereas the helicase domain partially contributed to this association in the presence of the N-terminal–disordered domain (Figure 7G). To verify this finding, we performed rescue experiments. Following DRB treatment, the 2 truncated ZNFX1 fragments containing the zinc finger domain notably protected the *Prkaa2* mRNA levels from degradation, nearly back to the level attained by the full-length ZNFX1. In contrast, other domains of ZNFX1 did not show such a rescuing effect (Figure 7H). Overexpression of ZNFX1 fragments containing the zinc finger domain strongly restored the bactericidal activity of BMDMs (Figure 7I).

Collectively, these findings demonstrate that *M*. *tuberculosis*–inducible ZNFX1 upregulation enhances AMPKα2 stability to promote autophagy, which subsequently suppresses intracellular *M*. *tuberculosis* proliferation and improves the activity of macrophages against *M*. *tuberculosis* (Figure 7J).

## Discussion

ZNFX1 is not only a ZFP but also a conserved member of helicases, which is the largest enzyme family in organisms found in all cellular lifeforms and many viruses (30, 31). It participates in all processes involving DNA or RNA (32–34). However, the association of ZFPs and helicases with TB remains unelucidated. Our study revealed that the absence of ZNFX1 contributed to an enhanced *M*. *tuberculosis* burden and compromised the anti–*M*. *tuberculosis* immune response. ZNFX1 contains the C-X(1-6)-H-X-C-X3-C(H/C)-X(3-4)-(H/C)-X(1-10)-C domain structure, which is a DNA-binding region also found in the human nuclear transcriptional repressor NF-X1 (35). However, ZNFX1 was found to function in macrophages as the stabilizer of *Prkaa2* mRNA via direct binding with the zinc finger region, promoting autophagy and enhancing the bactericidal activity of macrophages. The evidence in this study demonstrates that ZNFX1 is crucial for anti–*M*. *tuberculosis* immune responses of macrophage.

Multiple biallelic mutations in the *ZNFX1* gene in humans have been associated with severe inherited immunodeficiency, such as MSMD, which presents with multiple clinical symptoms, including monocytosis, neutrophilia, thrombocytopenia, hepatomegaly, and splenomegaly (19). Among 2 patients with bacillus Calmette-Guérin (BCG) disease, disseminated TB, and intermittent monocytosis, *ZNFX1* was the only common gene with 2 rare nonsynonymous homozygous mutations predicted to result in loss of function (36). ZNFX1 recognizes dsRNA and promotes type I IFN response in the early stage of viral infection, thereby enhancing antiviral immune effects (20). In contrast, upon stimulation with dsRNA or dsDNA, primary cells from patients with autosomal recessive immunodeficiency carrying deleterious variants of *ZNFX1* showed an extended half-life of ISG mRNA, unbalanced innate immune response, and decreased clearance of virus infection (23). Moreover, in silico analysis predicted the absence of a signal peptide sequence targeting specific organelles in ZNFX1 (37). ZNFX1 has been found in ribonucleoprotein granules called stress granules (36), *Caenorhabditis elegans* germ granules (i.e., P granules) in early germline blastomeres (38), and liquid-like perinuclear condensates (i.e., nuage) in the germline (39). These non-membrane-bound organelles are formed by the spontaneous self-assembly of RNAs and specific proteins, such as RBPs, playing key roles in the epigenetic regulation of gene expression. To date, little evidence is available on the effects of ZNFX1 mutation on TB, possibly due to the lack of available materials. Individuals with deficiencies in ZNFX1 have been proposed to suffer more from TB than from MSMD, as the virulence of *M*. *tuberculosis* is approximately 1,000 times greater than that of BCG (36). Consistently, in our study, we observed that the absence of ZNFX1 resulted in a basal defect in phagocytosis and the expression of costimulators, including CD80, CD86, and MHC-II. Our results, along with previous study findings, indicate the importance of ZNFX1 in maintaining the basal level of host immunity.

Autophagy is activated in immune cells upon recognition of pathogen-associated molecular patterns or damage-associated molecular patterns to modulate bactericidal activities, antigen presentation, and cytokine production (3, 40). As a critical regulatory mechanism for immune metabolism and cellular nutrient homeostasis, autophagy participates in the self-renewal, survival, differentiation, and function of various immune cells, including neutrophils (41), B1a cells (42), NKT cells (43), peripheral effector T cells (44), CD8^+^ memory T cells (45), and monocytes (46). Moreover, autophagy is a potent mechanism for counteracting the suppression of phagolysosome-related antibacterial activity by *M*. *tuberculosis* (47, 48). It also plays a crucial role as a key regulatory factor in the process of epigenetic reprogramming during the induction of training immune memory formation by BCG (49). For these reasons, autophagy is considered a natural immune defense mechanism that effectively controls intracellular *M*. *tuberculosis*. Thus, the regulation of autophagy by ZNFX1 plays a crucial role in establishing anti–*M*. *tuberculosis* immune responses.

ULK1 serves as the key regulator for autophagy initiation, bridging the upstream nutrient or energy receptors mTOR or AMPK with the downstream autophagosomes in vivo. During glucose starvation, AMPK catalyzes the activation of ULK1, which can be disrupted by high mTOR activity under nutrient sufficiency (50). In our study, high-throughput RNA-sequencing analysis revealed that *Prkaa2*, the gene coding for AMPKα2, is stably and positively correlated with ZNFX1 expression. When upregulated and activated by epigallocatechin-3-gallate pretreatment, AMPKα2 promotes adaptive autophagy, increases energy supply, and maintains mitochondrial function in response to doxorubicin-induced cardiotoxicity (27). Moreover, phosphorylation of Ser495 in phosphatase and tensin homolog–induced putative kinase 1 by AMPKα2 is essential for efficient mitophagy and improvement of mitochondrial function, preventing the progression of heart failure (HF). When the dominant AMPKα isoform switches from AMPKα2 to AMPKα1, HF is accelerated (51). In our study, in vitro and in vivo pharmaceutical treatments using the AMPK stimulator EX229 and ectopic expression of *Prkaa2* restored macrophage immune responses that were impaired due to *Znfx1* knockout. This further demonstrated the importance of *Prkaa2* and *Prkaa2*-involved autophagy in the roles of ZNFX1 against *M*. *tuberculosis* infection.

Helicases can be classified into 6 superfamilies based on their sequence, structure, and functional motifs (52). ZNFX1 is a member of the Upf1-like subfamily in the superfamily 1 (SF1) of helicases. Its function has been explored much less than that of the SF2 superfamily, especially in the field of antiviral immune responses. The zinc finger domain is located at the C-terminal of the helicase core region, and it is predicted to determine the primary function of defined helicases, including ZNFX1 (52). ZNFX1 was predicted to localize in the nucleus with over 80% probability, as it contains a nuclear localization motif. This prediction was made using the online protein subcellular localization prediction tool PSORT (https://www.genscript.com/psort.html). We observed that nucleus-localized ZNFX1 was present exclusively in undifferentiated murine hematopoietic progenitor cells obtained from murine BM (data not shown) and not in differentiated macrophages. The translocation of ZNFX1 may be a regulatory mechanism of ZNFX1 function. The subcellular localization of ZNFX1 in both the cytosol and membrane portions in macrophages, as found in our study, excluded its transcriptional regulatory role during the immune response. Our study findings demonstrated the association between ZNFX1 and *Prkaa2* mRNA, which suppressed the degradation of *Prkaa2* mRNA and, in turn, promoted the bactericidal activity of macrophages. This indicated the essential role of *Prkaa2* in mediating the anti–*M*. *tuberculosis* immunoregulatory function of ZNFX1. Further molecular events in the stabilization of *Prkaa2* mRNA by ZNFX1 warrant investigation, using more accurate and direct research methods, such as cryo-electron microscopy.

To date, the reported function of ZNFX1 has been restricted to ISG signaling, where the absence of ZNFX1 is associated with the extended half-life of ISGs and a loss of control over virus infection (20, 23, 53). Similarly, the absence of ZNFX1 led to an elevation in ISG expression in response to H37Rv, as observed in our study, though it did not influence the intracellular *M*. *tuberculosis* burden. In contrast with previous reports, no significant difference in ISG expression levels following LPS and VSV stimulation was observed between WT and *Znfx1*^–/–^ BMDMs. Such differences may be due to variations in the stimulators and cell types used, but not necessarily due to species diversity, given the high similarity in sequence and functional domains between human and mouse ZNFX1. Our experimental evidence demonstrated that the expression and immunoregulatory functions of ZNFX1 in response to *M*. *tuberculosis* infection were similar in both human and murine macrophages. Therefore, our study revealed a function of ZNFX1 in stabilizing *Prkaa2* mRNA, which is critical for the outcome of *M*. *tuberculosis* infection. Additionally, previous and current observations indicated that the function and mechanism of ZNFX1 may differ under different pathophysiology conditions, likely due to the complex regulatory network of RBPs (10). RBPs are involved in the complex regulatory network of life processes by interacting with RNA, DNA, and proteins. Their crucial roles in immune responses, such as modulating signaling pathways, controlling cytokine production, regulating immune cell activation and immune homeostasis, and influencing regulators like MCPIP1, TTP, and roquin 1, have been well recognized (54). However, our understanding of the regulatory networks of RBPs remains far from complete (10). Elucidation of these networks will enhance our insights into the regulation of multiple life processes. Moreover, although our study demonstrated that ZNFX1 selectively stabilized *Prkaa2* but not *Prkaa1*, it does not exclude the possibility that ZNFX1 may regulate other RNA species, resulting in similar phenotypes. Therefore, reanalyzing the RNA-sequencing (RNA-Seq) data or performing RIP-Seq is expected to reveal additional RNA species regulated by ZNFX1 during *M*. *tuberculosis* infection, which will shed light on the immune regulation against *M*. *tuberculosis* infection by ZFPs. These efforts have been undertaken and are currently in progress.

In summary, our study revealed that in the macrophage-mediated immune response against *M*. *tuberculosis* infection, the upregulation of ZNFX1 served as a protective strategy that promotes autophagy. This, in turn, contributed to the suppression of intracellular bacterial survival. These enhanced systemic immune responses led to the rescue of lung tissue injury and the prevention of inflammatory infiltration in lung and spleen tissues. In this process, ZNFX1 functions as an RBP to stabilize the key activating subunit of AMPK, maintaining autophagy to support the immune response. Our study expands the knowledge about the role of ZFPs in immune modulation and introduces a promising avenue for interference-based strategies in anti-TB immunotherapy.

## Methods

### Mice, cells, and agents.

Specific pathogen–free C57BL/6J mice were provided by Southern Medical University Laboratory Animal Management Centre of Southern Medicine University (Guangzhou, China). The experimental protocol was approved by the Biosafety Management Committee and the Medical Ethics Committee of Southern Medical University. The *Znfx1*^–/–^ mouse strain with C57BL/6 background was gifted by Anlong Xu (Beijing University of Chinese Medicine, Beijing, China) and was purified by Guangdong Laboratory Animals Monitoring Institute (Guangzhou, China). All female mice were used at the age of 6–8 weeks and were randomly divided into each experimental group. Genetically modified mice were identified by obtaining murine genome from tails using tissue DNA extraction kit (Omega Biotek) followed by PCR and standard agarose gel electrophoresis detection. Sequences of primers are listed in Supplemental Table 2. All these mice were bred at Southern Medical University Laboratory Animal Management Centre. Femurs of 6- to 8-week-old female mice were obtained and incubated in DMEM added to 40 ng/mL of murine M-CSF (PeproTech) for 5 days, with half medium replacement every 3 days, thus obtaining primary BMDMs.

Human CD14^+^ peripheral blood monocytes were positively separated from peripheral blood mononuclear cells (PBMCs) using MACS and cultured in RPMI 1640 medium (Corning) supplemented with 10% FBS (Corning), 2-ME (Thermo Fisher Scientific), and 100 ng/mL M-CSF (PeproTech) at 37°C with 5% CO_2_ for 7 days for induction of hMDMs. The culture medium was half replaced every 2 days. Human monocytic leukemia cell line THP-1 and human embryo kidney cell line HEK293T were purchased from American Type Culture Collection (ATCC) and cultured in RPMI 1640 (for THP-1) or DMEM (for HEK293T) medium (Corning) supplemented with FBS. Before experiments, THP-1 cells were treated with 100 ng/mL of PMA (Thermo Fisher Scientific) for 24 hours to differentiate into macrophages (THP-1 macrophages), and then the PMA-containing medium was replaced with the serum-free medium for a further 24 hours for rest.

The cells were treated with the following agents as indicated in the figure legends: EX229 (20 μM), CHX (50 μM) (MedChemExpress [MCE]); BafA1 (50 ng/mL; Santa Cruz Biotechnology), rapamycin (50 nM), DRB (50 μg/mL) (Merck Millipore); and murine IFN-γ (2 ng/mL, PeproTech).

### M. tuberculosis culture, infection, and CFU assay.

Standard *M*. *tuberculosis* strain H37Rv (ATCC 27294) was cultured in Difco Middlebrook 7H9 Broth (BD Biosciences) supplemented with 1:9 volume of oleic acid-albumin-dextrose-catalase and 0.05% Tween 80 (Merck Millipore) at 37°C with 5% CO_2_. For experiments, the suspension of H37Rv in the midlog phase was centrifuged at 1,500*g* at 25°C for 5 minutes and resuspended in serum-free DMEM containing 0.05% Tween 80. After grinding 30–50 times, the achieved homogenate containing a single bacterium was centrifuged again at 1,500*g* at 25°C for 5 minutes, and the obtained supernatant was detected using Biophotometer Plus spectrophotometer (Eppendorf) at OD 600 nm. The concentration of bacterium suspension with OD value detected at 600 nm of 0.207 was regarded to be 4 × 10^6^ colonies/mL. Varied MOI of H37Rv was used as indicated in different experiments. For detection of intracellular *M*. *tuberculosis* load, CFU assays were performed as previously reported (55). To compare the effects on intracellular *M*. *tuberculosis* survival in the face of different phagocytosis amount, the amounts of *M*. *tuberculosis* in WT or *Znfx1*^–/–^ BMDMs at different times postinfection were normalized to the average *M*. *tuberculosis* amount in each type of cells at 0 hours.

### RNA extraction, qPCR, and high-throughput RNA-Seq.

Total RNA of cells was extracted with TransZol (Transgen Biotech). Standard agarose gel electrophoresis was used to assess RNA integrity, and the RNA concentration and purity were detected using NanoDrop 2000 Ultraviolet-Vis Spectrophotometer (Thermo Fisher Scientific). Target gene expression was analyzed following reverse transcription of total RNA using TransScript One-Step gDNA Removal and cDNA Synthesis SuperMix kit (Transgen Biotech) and qPCR using TransStart Top Green qPCR SuperMix kit (Transgen Biotech) on the LightCycler 96 (Roche). The expression of β-actin was used as the reference normalization for quantification of target mRNA abundance with the 2^−ΔΔCT^ method.

For high-throughput RNA-Seq, 1 line of WT BMDMs and 2 lines of *Znfx1*^–/–^ BMDMs were infected with or without H37Rv at MOI = 2 for 6 and 24 hours. RNA from TransZol-lysed cells was analyzed on an Illumina HiSeq 2500 by Ribobio Co., Ltd. RNA-Seq data were aligned to mouse reference genome sequence (GRCm38 assembly) using HISAT2 (56). Differentially expressed genes (DEGs) between WT and each line of *Znfx1*^–/–^ BMDMs under mock or H37Rv infection at each time point were screened out according to the criteria log_2_FC > 1 and *P* < 0.05. Kyoto Encyclopedia of Genes and Genomes (KEGG) analysis was used to screen out the potentially affected signaling pathways by *Znfx1* knockout, and Venn analysis was used to find out the common KEGG pathways under mock or H37Rv infection, which included the 2 time points and the comparison groups between WT BMDMs and each line of *Znfx1*^–/–^ BMDMs. Subsequently, the DEGs enriched in the common KEGG pathways in BMDMs without infection, and in BMDMs with H37Rv infection, with the appearance frequency greater than or equal to 3, were analyzed using Venn analysis again to find out genes generally regulated by ZNFX1. Sequences of primers are listed in Supplemental Table 2.

### Western blotting, subcellular fractionation, and co-IP.

Cells were treated as indicated and washed with cold 1× PBS. Cells were lysed and total protein was extracted with RIPA buffer (20 mM HEPES at pH 7.9, 400 mM NaCl, 1 mM EDTA, 10 mM KCl, 20% glycerol, 1% NP-40, 0.5% sodium deoxycholate, 0.1% sodium dodecyl sulfate, supplemented with 1:10 volume of PhosSTOP Phosphatase Inhibitor Cocktail from F. Hoffmann-La Roche, 1 mM DTT, 1 mM PMSF). Proteins were separated using SDS-PAGE after protein quantification with the Bradford agent (Bio-Rad Laboratories), then blotted on the PVDF membrane (Merck Millipore) using the following antibodies with the dilution of 1:1,000 if not indicated otherwise: p-AMPKα (catalog 2535), AMPKα (catalog 5831), p-Akt (catalog 4060), Akt (catalog 9272), p-p38 MAPK (catalog 4511), p38 MAPK (catalog 8690), ERK1/2 (catalog 9102), p-ERK1/2 (catalog 4370), NF-κB p65 (catalog 4764), ULK1 (catalog 8054), p-mTOR (catalog 5536), mTOR (catalog 2972), p-p70S6K (catalog 9206), p70S6K (catalog 9202), p-4E-BP1 (catalog 2855), 4E-BP1 (catalog 9644), LC3I/II (catalog 12741), SQSTM1/p62 (catalog 5114), HA-Tag (catalog 3724), p-STAT1 (catalog 9167) (Cell Signaling Technology, CST); p-ULK1 (catalog 80218-1-RR), Flag tag (catalog 66008-4-Ig), STAT1 (catalog 166545-1-Ig) (Proteintech); p–NF-κB p65 (catalog AP0123, ABclonal Technology Co., Ltd); anti-human ZNFX1 (catalog ab179452, Abcam); anti-mouse ZNFX1 (customized by Dia-an Biosciences, no clone number or reference available); β-actin (1:5,000; catalog HC201, Transgen Biotech); and the corresponding HRP-conjugated secondary antibodies [1:5,000; Goat anti-Mouse IgG (H+L) catalog 31430, Goat anti-Rabbit IgG F(ab′)2 catalog 31234, Thermo Fisher Scientific]. The signals were developed using FDbio-Pico ECL (Hangzhou Fude Biological Technology Co., Ltd.) and obtained using FluorChem (ProteinSimple). ImageJ 1.53 (National Institutes of Health) was used to quantify the levels of proteins.

To detect the intracellular localization of ZNFX1, subcellular fractionation of BMDMs was performed using the Nucl-Cyto-Mem Preparation Kit (Applygen Technologies) as per the instruction of the manufacturer. The obtained cellular fractions of protein were analyzed using Western blotting, with Na,K-ATPase (catalog 3010), Lamin A/C (catalog 4777) (both from CST), and GAPDH (catalog 10494-1-AP) (Proteintech) as the reference proteins of membrane, nucleus fractions, and cytosol, respectively.

co-IP assays were performed using Protein A/G magnetic beads (MCE) with 2 μg of antibodies against ZNFX1 (customized by Dia-an Biosciences), AMPKα2 (catalog 18167-1-AP), and STK11/LKB1 (catalog 10746-1-AP) (both from Proteintech) and rabbit IgG isotype control (catalog I5006) (Merck Millipore), according to the following protocol. Briefly, 10^7^ BMDMs were collected after treatment, washed twice with PBS, and resuspended in IP buffer (containing 50 mM Tris-HCl at pH 8.8, 5 mM EDTA, 1% NP-40, 150 mM NaCl, 1 piece of proteinase inhibitor from Roche, pH adjusted to 7.2–7.4, diluted with ultrapure water to 50 mL) at 4°C for 20 minutes. Samples were then lysed with ultrasound (10 cycles of 30 seconds on/30 seconds off at 4°C; Diagenode Bioruptor) and centrifuged at 4°C, 11,000*g*, for 10 minutes. The supernatant was collected and quantified. Next, 2 mg of protein was coincubated with 2 μg of antibody at 4°C overnight, then mixed with 50 μL of the Protein A/G magnetic beads for further incubation at 4°C for 2–3 hours followed by washing with IP buffer 3 times on the magnetic shelf (MCE). The beads-protein-antibody mix was washed with the RIP buffer 5 times, and 50 μL of 2× loading buffer containing 5% 2-ME was added. Following separation of the magnetic beads and the supernatant, proteins in the supernatant were revealed using Western blotting as indicated in the figures.

### Immunohistochemistry.

Paraffin-embedded TB patient lung and LN tissues were provided and sliced into 5 μm–thick sections by Guangzhou Chest Hospital with the written consent of all participants. Levels of ZNFX1 in vivo were detected with immunohistochemistry (IHC) staining according to the previously described protocol (57), with the antibody against ZNFX1 (1:50; catalog HPA046629; Merck Millipore) and HRP-conjugated goat anti-rabbit IgG (H+L) (1:200; catalog 31460; Thermo Fisher Scientific). Paraffin-embedded sections of murine lungs and spleens were stained with anti-F4/80 antibody (1:100; catalog MA5-16630; Thermo Fisher Scientific) together with anti-LC3 antibody (1:100; catalog 14600-1-AP; Proteintech) or anti–p-AMPK antibody (1:100; catalog AF3423; Affinity) at 4°C overnight, followed by staining with goat anti-mouse IgG 488 (catalog A28175) for F4/80 and goat anti-rabbit IgG 647 (catalog A27040) (Thermo Fisher Scientific) for LC3 and p-AMPK at room temperature for 1 hour. Samples were then scanned with CaseViewer2.4 (3DHISTECH). The levels of ZNFX1 in the TB tissues and the levels of LC3 puncta or p-AMPK in macrophages in murine lung and spleen tissues were determined using ImageJ 1.53 by a reader following a protocol blinded to the knockout versus WT experimental condition.

### Animal experiments.

To assess the effects of ZNFX1 in anti–*M*. *tuberculosis* immune response, WT and *Znfx1*^–/–^ mice (*n* = 5) were exposed to 10^7^ of H37Rv in the aerosol generation device (Glas-Col) for 24 hours. Mice were sacrificed 1 and 4 weeks postinfection. About 200 μL of eye blood was collected and centrifuged at 1,500*g* at 25°C for 5 minutes, then frozen at –80°C for subsequent Luminex multiplex assays using ProcartaPlex Mix&Match Luminex Multiplex Assays (Thermo Fisher Scientific).

Part of the spleen and lung tissues was suspended in PBS and ground into single cells. The supernatants were stored at –80°C for ELISAs (Multi Sciences [Lianke] Biotech, Co., Ltd.) and detection of NO production with the Griess Reagent System (Promega [Beijing] Biotech Co., Ltd.) according to the instructions of the producers. Cells were lysed with 0.2% Triton-PBS for detecting bacterial loads using CFU assays. Another part of tissues was cut into 5 μm–thick slices following fixation with 4% paraformaldehyde-PBS and paraffin-embedded. H&E staining was used to assess tissue inflammation and damage, and IHC staining was performed to detect target proteins as indicated. In some experiments, mice were treated with 200 μM of EX229 through intraperitoneal injection 1 week after H37Rv infection.

### Flow cytometry analysis.

To analyze the phagocytosis activity of *Znfx1*^–/–^ BMDMs, cells were infected with H37Rv-RFP or incubated with FITC-conjugated Latex beads (catalog L4530, Merck Millipore). Cells were then collected and analyzed with flow cytometry.

To detect the numbers of macrophages in various tissues of *Znfx1*^–/–^ mice, single cells obtained from the spleens, the lungs, and the LNs of WT or *Znfx1*^–/–^ mice were incubated in 1% FBS-PBS containing the following antibodies in the dark at 4°C for 30 minutes before flow cytometry analysis: PerCP/Cyanine5.5 anti-mouse/human CD11b (M1/70; BioLegend), Violet Fluor 450–F4/80 (BM8.1; Tonbo Bioscience), and PE-Ly6c (HK1.4; BioLegend).

To assess the activation of BMDMs infected with H37Rv, cells were collected with trypsinization and incubated in 1% FBS-PBS containing the following antibodies at 4°C in the dark for 30 minutes: FITC-CD80 (16-10A1; Thermo Fisher Scientific), APC-CD86 (GL-1; Tonbo Bioscience), PE-Cy7–MHC-II (M5/114.15.2; Invitrogen), eFluor 450–CD11b (M1/70; Tonbo Bioscience), and PE-CD206 (C068C2; BioLegend). Cells were then centrifuged at 300*g* at 4°C for 5 minutes, fixed with 4% paraformaldehyde, and analyzed.

Cells were detected using Attune NxT flow cytometry (Thermo Fisher Scientific), and the data were analyzed with FlowJo 10 software (BD Biosciences).

### Immunofluorescence staining and confocal microscopy observation.

Macrophages were seeded on glass coverslips at 1 × 10^4^/500 μL RPMI 1640 in 12-well plates for 24 hours, then treated with EX229 followed by infection with H37Rv or treated with 50 ng/mL of BafA1 for 2 hours followed by infection with H37Rv-RFP. Intracellular LC3-I/II was assayed using immunofluorescence staining using anti–LC3-I/II antibody (1:200) (clone 12741, CST) as previously described (58). Cells were then counterstained with DAPI and observed with an Axiovert LSM 880 confocal laser scanning microscope (Carl Zeiss Microscopy). The LC3-I/II puncta and *M*. *tuberculosis*–containing vacuoles were quantified using ImageJ 1.53 software as reported previously (59).

### Recombinant plasmid construction and nucleic acid transfection.

Nucleic acid segments encoding *Prkaa2*, and full-length or function domain construct F2 of *Znfx1* with Flag tag, were subcloned into the recombinant pHAGE lentivirus plasmid and packaged to recombinant lentiviruses to infect BMDMs. F1 and F3–5 function domains of *Znfx1* were subcloned into the eukaryotic expression plasmid pcDNA3.1 with a Flag tag at the C-terminal. Recombinant pcDNA3.1 plasmids were transfected into HEK293T cells using polyethylenimine linear MW 40,000 (Yeasen Biotechnology [Shanghai] Co., Ltd.) as per the manufacturer’s instruction. Small RNAs targeting human *ZNFX1* and a scramble oligonucleotide were synthesized (Guangzhou Ribobio Co., Ltd) and then transfected into hMDMs using Lipofectamine 2000 (Thermo Fisher Scientific) according to the protocol of the manufacturer. Forty-eight hours later, the overexpression and silencing efficiency were detected using qPCR and Western blotting before further experiments. The targeting sequences of siRNAs are in Supplemental Table 2.

### RIP.

The binding of ZNFX1 with target mRNA was assayed according to a previously reported protocol (60), with some modifications as described below. Following treatment, 10^7^ of BMDMs or HEK293T cells were collected and resuspended in RIP buffer (i.e., IP buffer supplemented with 100 U/mL RNase inhibitor) at 4°C for 20 minutes. Samples were then lysed with ultrasound (5 cycles of 30 seconds on/30 seconds off at 4°C) and centrifuged at 4°C, 11,000*g*, for 10 minutes. The supernatant was collected and quantified. Next, 5–10 mg of protein was coincubated with 5–10 μg of antibody at 4°C overnight, then mixed with the Protein A/G magnetic beads for further incubation at 4°C for 2–3 hours. The beads-protein-antibody mix was washed with the IP buffer 3 times on the magnetic shelf, and 200 μL of RNase-free sterile water containing 40 μg/mL of proteinase K (Merck Millipore) was added. Samples were incubated at 45°C for 50 minutes, then purified with TransZol. Coprecipitated RNAs were isolated for further qPCR detection for *Prkaa1* and *Prkaa2*.

### RNA pulldown.

RNA pulldown was performed as previously described (61, 62). Biotinylated Prkaa2 and GFP transcripts were transcribed using High Yield T7 Biotin16 RNA Labeling Kit (APExBIO Technology) as per the manufacturer’s protocol.

### Statistics.

The representative data of at least 3 independent experiments were presented as means ± SD. A *t* test was used to compare the difference when 2 parameters were involved. The difference of a response variable affected by more than 3 (inclusive) parameters of 1 factor was compared using 1-way ANOVA. Two-way ANOVA was used to analyze the influence of 2 independent factors on a response variable and determine the existence of interaction between the 2 factors on this response variable. Holm-Šídák method was used for post hoc multiple comparisons. *P* < 0.05 indicated that the difference in treatment groups was statistically significant. All statistical analyses were performed using GraphPad Prism 9.4.1.

### Study approval.

The use of TB patient lung tissues was approved by the Ethics Committee at Guangzhou Chest Hospital. The PBMCs of healthy volunteers were provided by Guangzhou Blood Center. All the experimental protocols were reviewed and approved by the Medical Ethics Board and the Biosafety Management Committee of Southern Medical University (approval number: SMU-L2018240). All patients provided written informed consent. Patient information is presented in Supplemental Table 1.

### Data availability.

RNA-Seq data have been submitted to NCBI BioProject with the accession code PRJNA929691. All data generated or analyzed during this study are included in this published article. Supporting Data Values are provided in the labeled XLS file for all values underlying data presented in graphs or as means in this article.

## Author contributions

HL, QW, and LM conceived and designed experiments. HL, ZH, LC, JZ, ZZ, YC, FL, KW, JL, and NS conducted experiments and acquired data. HL, ZH, LC, QW, XZ, CZ, SH, and LM analyzed data. QW, HL, and LM wrote the manuscript. QW and LM provided reagents and reviewed and edited the manuscript. The order of the 3 co–first authors depended on their workload and contribution to this study.

## Supplementary Material

Supplemental data

Unedited blot and gel images

Supplemental table 1

Supplemental table 2

Supplemental table 3

Supporting data values

## Version 1 11/28/2023

In-Press Preview

## Version 2 01/09/2024

Electronic publication
