# Supplementary material for: ZNFX1 promotes AMPK-mediated autophagy against Mycobacterium tuberculosis by stabilizing Prkaa2 mRNA
Source: JCI Insight. 2024 Jan 9;9(1):e171850. doi: 10.1172/jci.insight.171850 (PMC10906457; doi:10.1172/jci.insight.171850)
Supplement: Supplemental data [file jciinsight-9-171850-s082.pdf]

Supplementary Figures for ZNFX1

Supplementary Figure 1

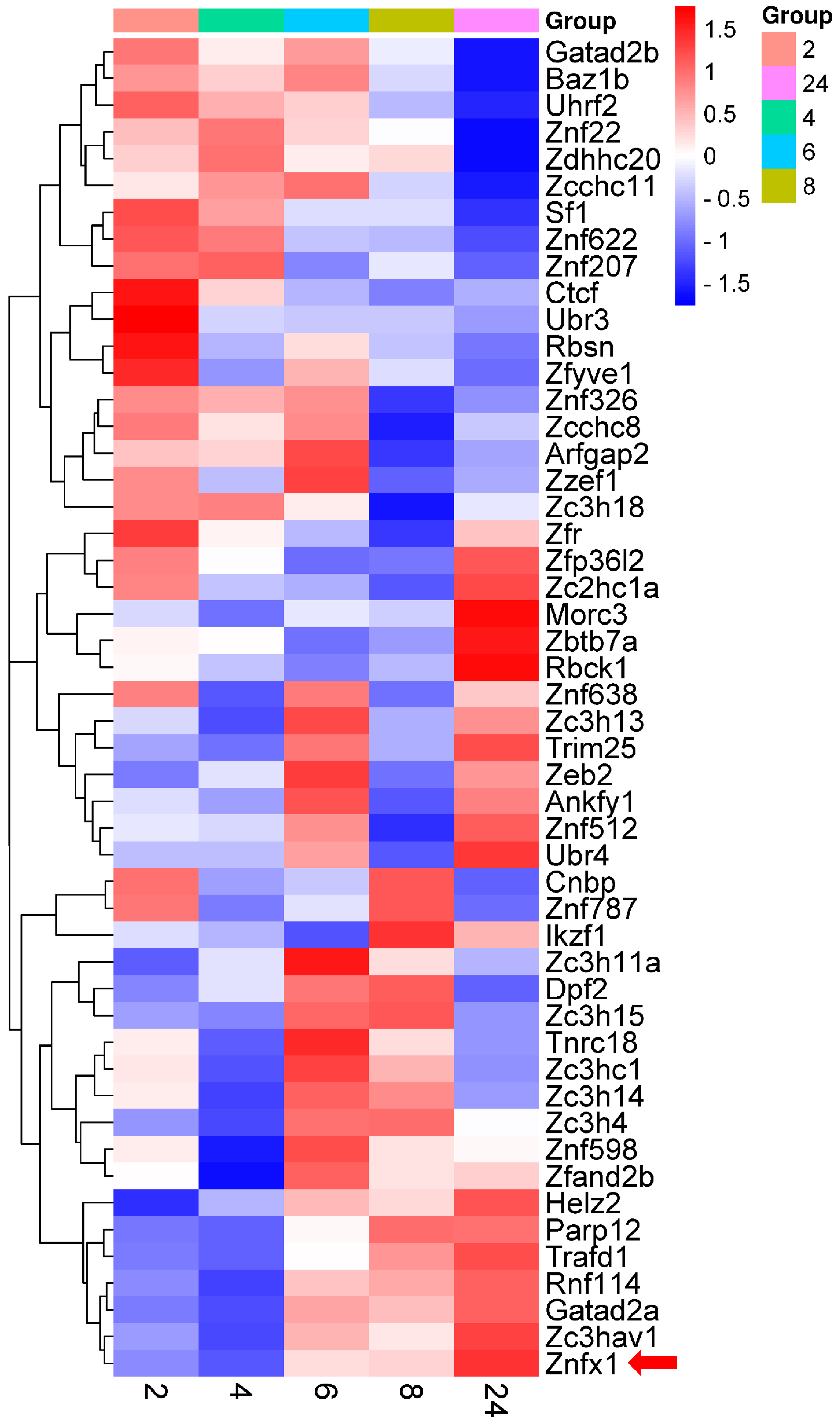

Supplementary Figure 1 for Figure 1. Heatmap of ZFP expression data from the study of Jonathan M. Budzik, et al. [18.]

Supplementary Figure 2

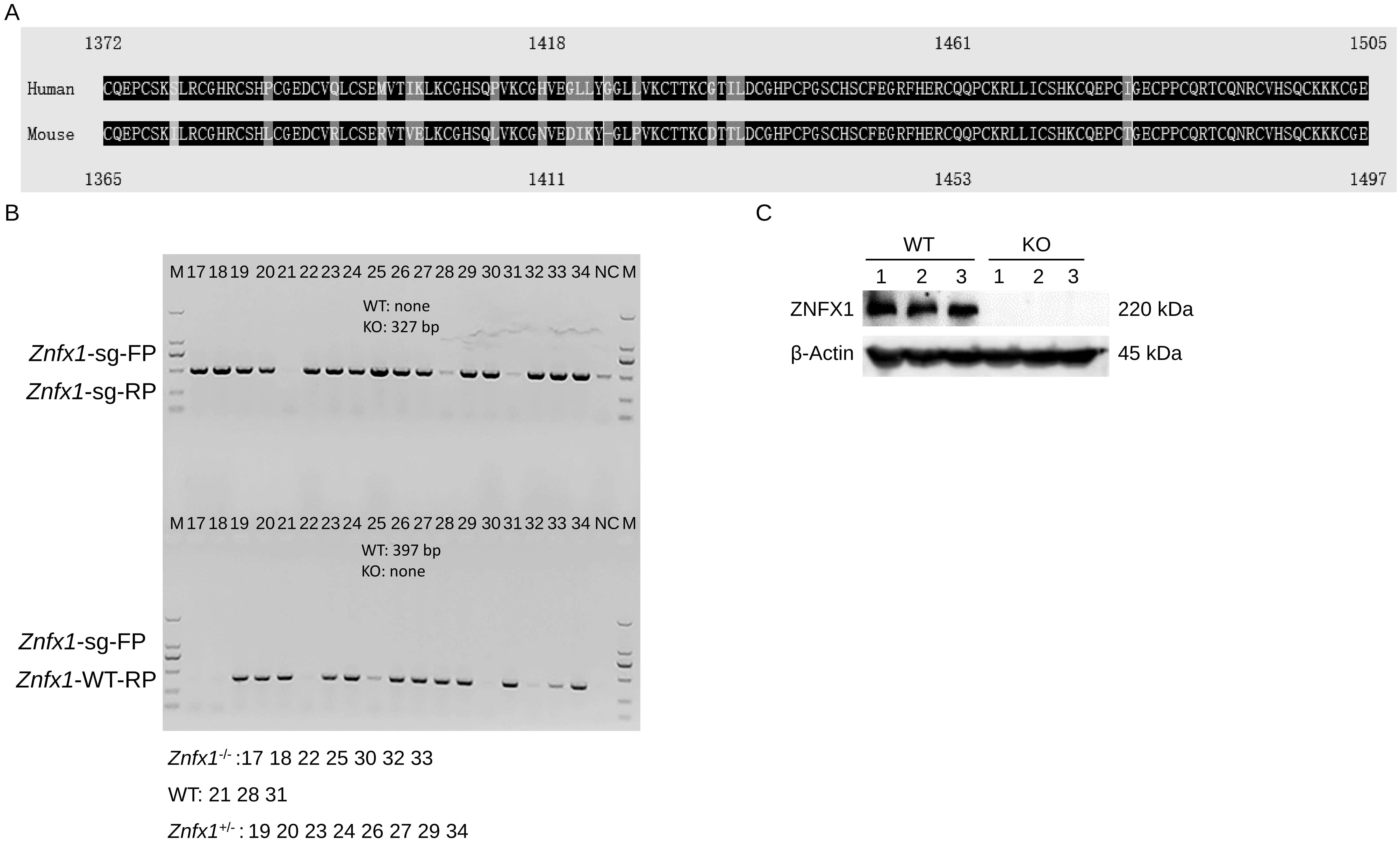

Supplementary Figure 2 for Figure 2. Sequence alignment of human and murine ZNFX1 proteins, and Identification of *Znfx1*$^{-/-}$ mice. (**A**) Sequence alignment of the zinc finger region of human and murine ZNFX1 proteins. (**B-C**) Identification of *Znfx1*$^{-/-}$ mice using PCR (**B**) and western blotting (**C**).

Supplementary Figure 3

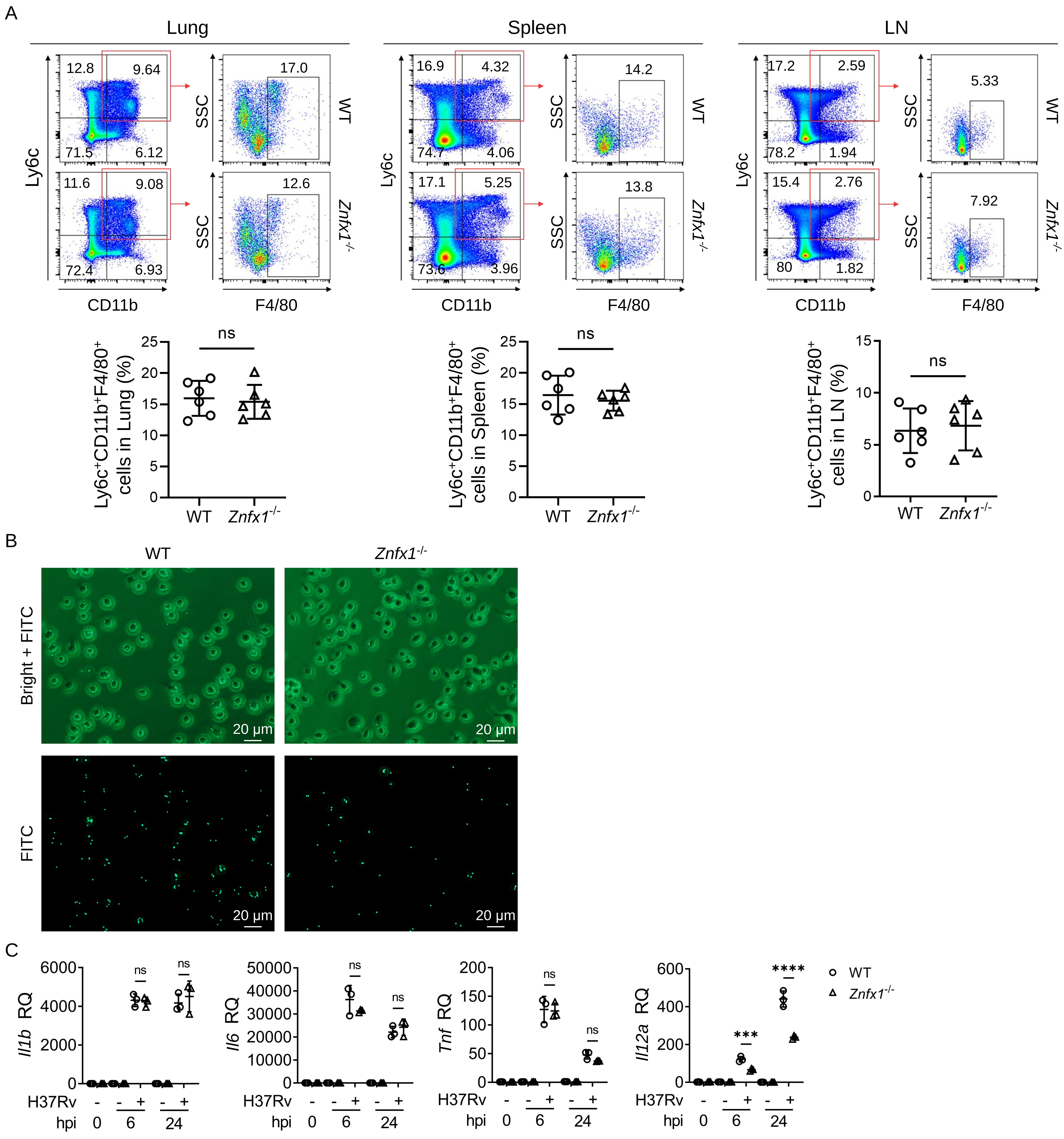

Supplementary Figure 3 for Figure 3. Amount and activity of *Znfx1*$^{-/-}$ Mφ. (**A**) Flow cytometry analysis of Ly6C$^+$CD11b$^+$F4/80$^+$ Mφ amounts in the lung, spleen and LN of WT and *Znfx1*$^{-/-}$ mice (n = 6). (**B**) Fluorescent photographs of green fluorescence positive WT and *Znfx1*$^{-/-}$ BMDMs incubated with FITC-conjugated Latex. (**C**) qPCR detection of cytokine expression in *Znfx1*$^{-/-}$ BMDMs infected with H37Rv (n = 3). An unpaired t test (A) and a two-way ANOVA with Šidák's post hoc test (C) was used for statistical analysis. Data are presented as mean ± SD and are representative of at least three experiments with similar observations. *P < 0.05; **P < 0.01; ***P < 0.001; ****P < 0.0001.

Supplementary Figure 4

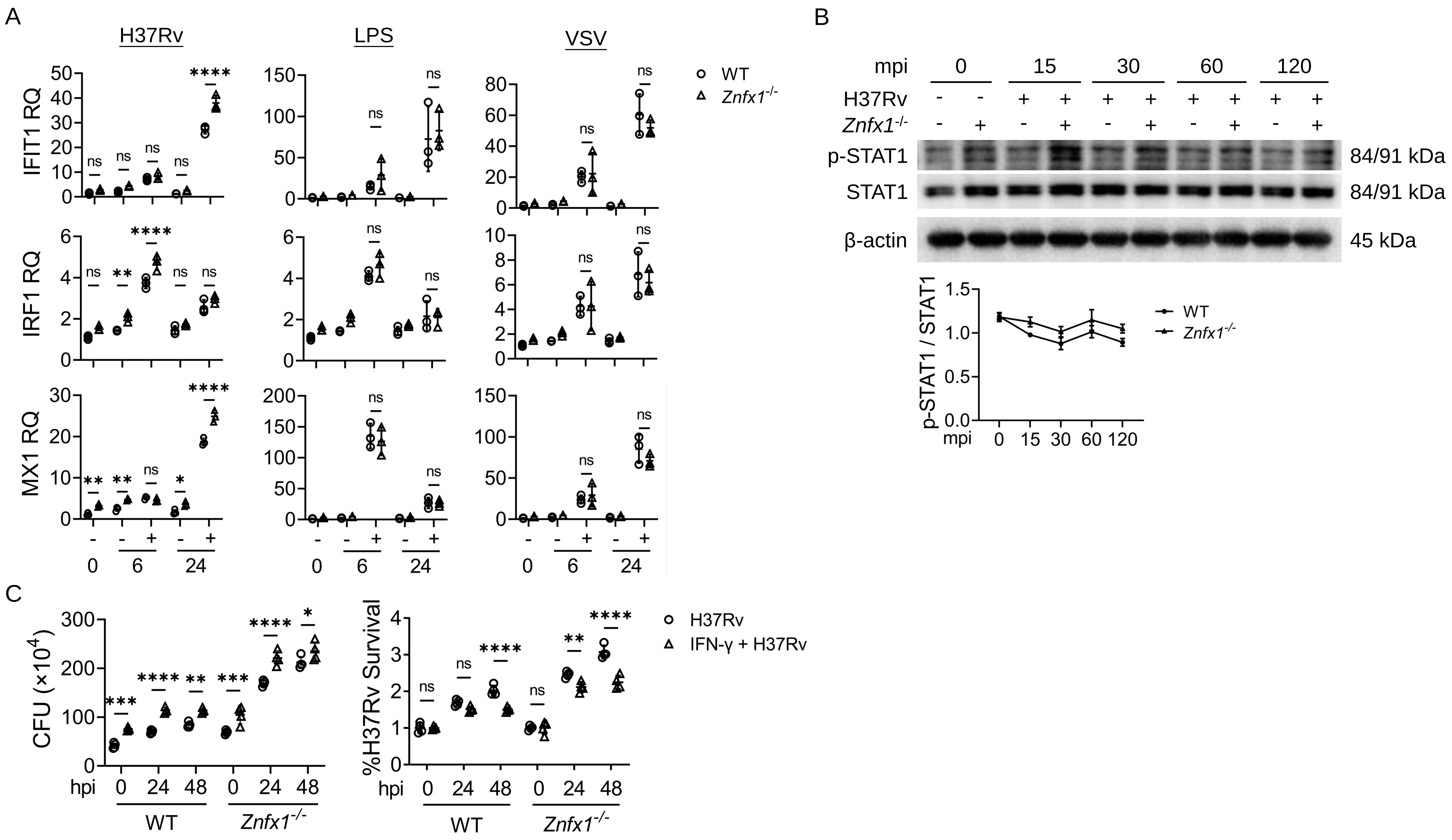

Supplementary Figure 4 for Figure 3. The ISG signaling pathway did not mediate the function of ZNFX1 in response to Mtb infection. (**A**) RT-PCR detection of ISG expression in *Znfx1*$^{-/-}$ BMDMs following stimulation with H37Rv, LPS and VSV, respectively (n = 3). (**B**) Western blot assay of activation of STAT1 in H37Rv-infected *Znfx1*$^{-/-}$ BMDMs. The relative levels of STAT1 phosphorylation was quantified (n = 3). (**C**) CFU assays of Mtb load in *Znfx1*$^{-/-}$ BMDMs treated with or without IFN-γ (n = 4). A two-way ANOVA with Šidák's post hoc test (A and C) was used for statistical analysis. Data are presented as mean ± SD and are representative of at least three experiments with similar observations. *P < 0.05; **P < 0.01; ***P < 0.001; ****P < 0.0001.

Supplementary Figure 5

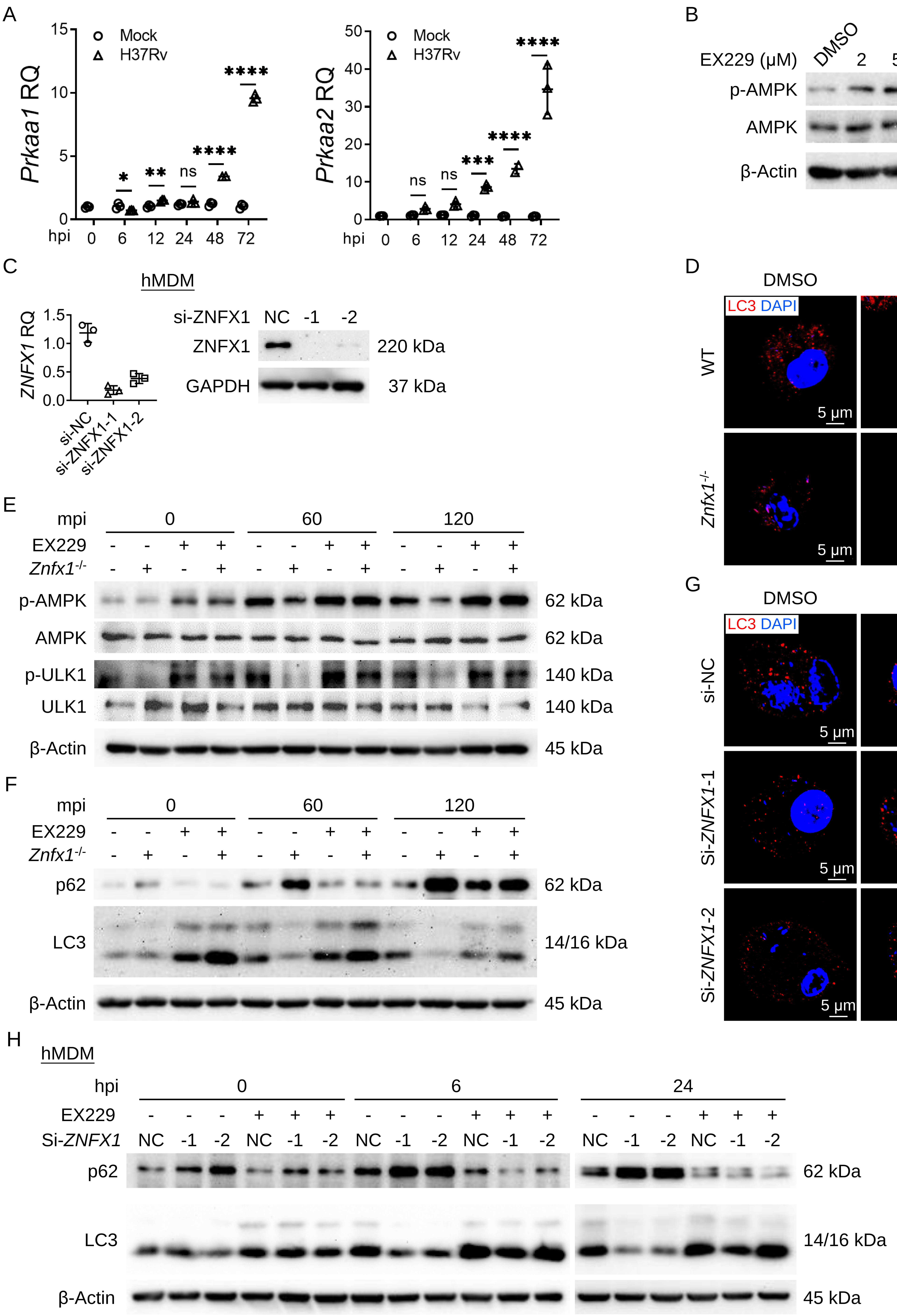

Supplementary Figure 5 for Figure 5. Recovery of AMPKα activation or expression promoted autophagy and bactericidal activity of Mφ resulted from absence of ZNFX1. (**A**) RT-PCR detection of *Prkaa1* and *Prkaa2* expression in H37Rv-infected *Znfx1*$^{-/-}$ BMDMs. (**B**) Western blot assay of AMPK activation following EX229 treatment of BMDMs. (**C**) RT-PCR and Western blot assay of ZNFX1 silencing in hMDMs. (**D**) Immunofluorescence staining of LC3 in H37Rv-infected *Znfx1*$^{-/-}$ BMDMs following EX229 treatment (30 randomly selected cells for statistics). (**E-F**) Western blot assay of activation of AMPK and downstream ULK1 (**E**), p62 level and LC3 conversion (**F**) in EX229-treated *Znfx1*$^{-/-}$ BMDMs followed by H37Rv infection (n = 3). (**G**) Immunofluorescence staining of LC3 in H37Rv-infected *ZNFX1*-silencing hMDMs following EX229 treatment (30 randomly selected cells for statistics). (**H**) Western blot assay of p62 level and LC3 conversion in EX229-treated ZNFX1-silencing hMDMs followed by H37Rv infection (n = 3). A two-way ANOVA with Šidák's post hoc test (A, D and G) was used for statistical analysis. Data are presented as mean ± SD and are representative of at least three experiments with similar observations. *P < 0.05; **P < 0.01; ***P < 0.001; ****P < 0.0001.

Supplementary Figure 6

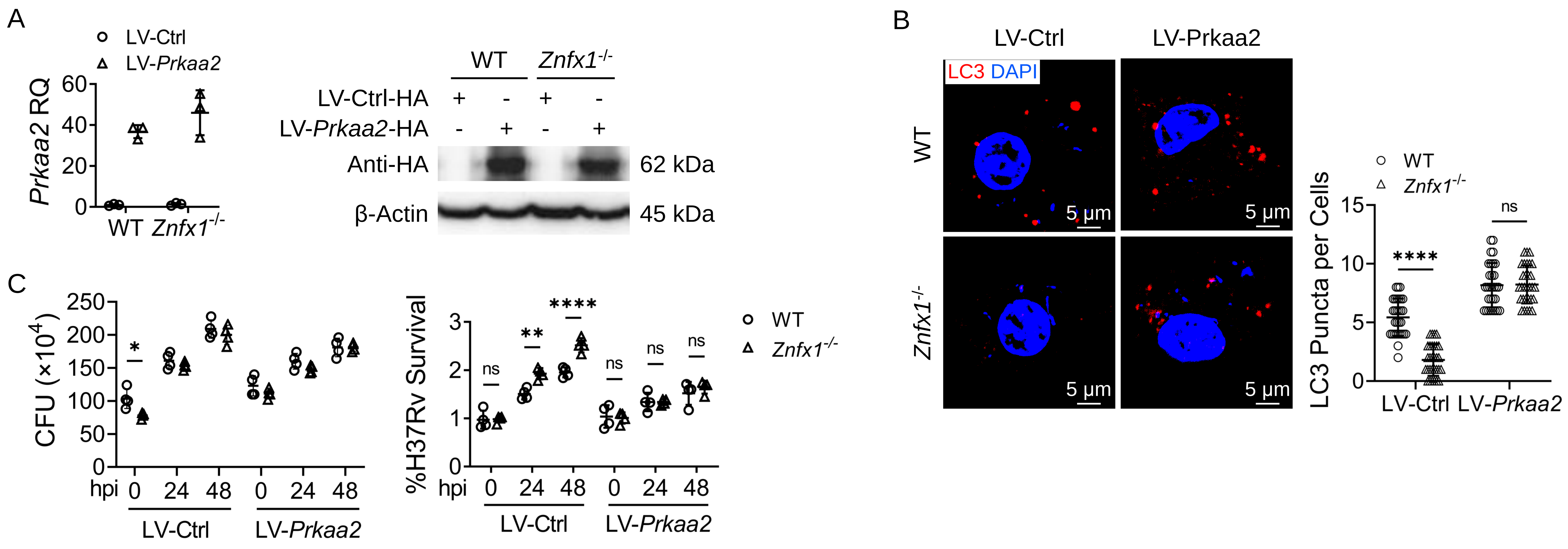

Supplementary Figure 6 for Figure 5. Recovery of AMPKα expression promoted autophagy and bactericidal activity of Mφ resulted from absence of ZNFX1. (**A**) RT-PCR and western blot assay of overexpression of *Prkaa2* in *Znfx1*$^{-/-}$ BMDMs (n = 3). (**B**) Immunofluorescence staining of LC3 in H37Rv-infected *Znfx1*$^{-/-}$ BMDMs overexpressing *Prkaa2* (30 randomly selected cells for statistics). (**C**) CFU assay of Mtb load in *Znfx1*$^{-/-}$ BMDMs overexpressing *Prkaa2* (n = 4). A two-way ANOVA with Šidák's post hoc test (B and C) was used for statistical analysis. Data are presented as mean ± SD and are representative of at least three experiments with similar observations. *P < 0.05; **P < 0.01; ***P < 0.001; ****P < 0.0001.

Supplementary Figure 7

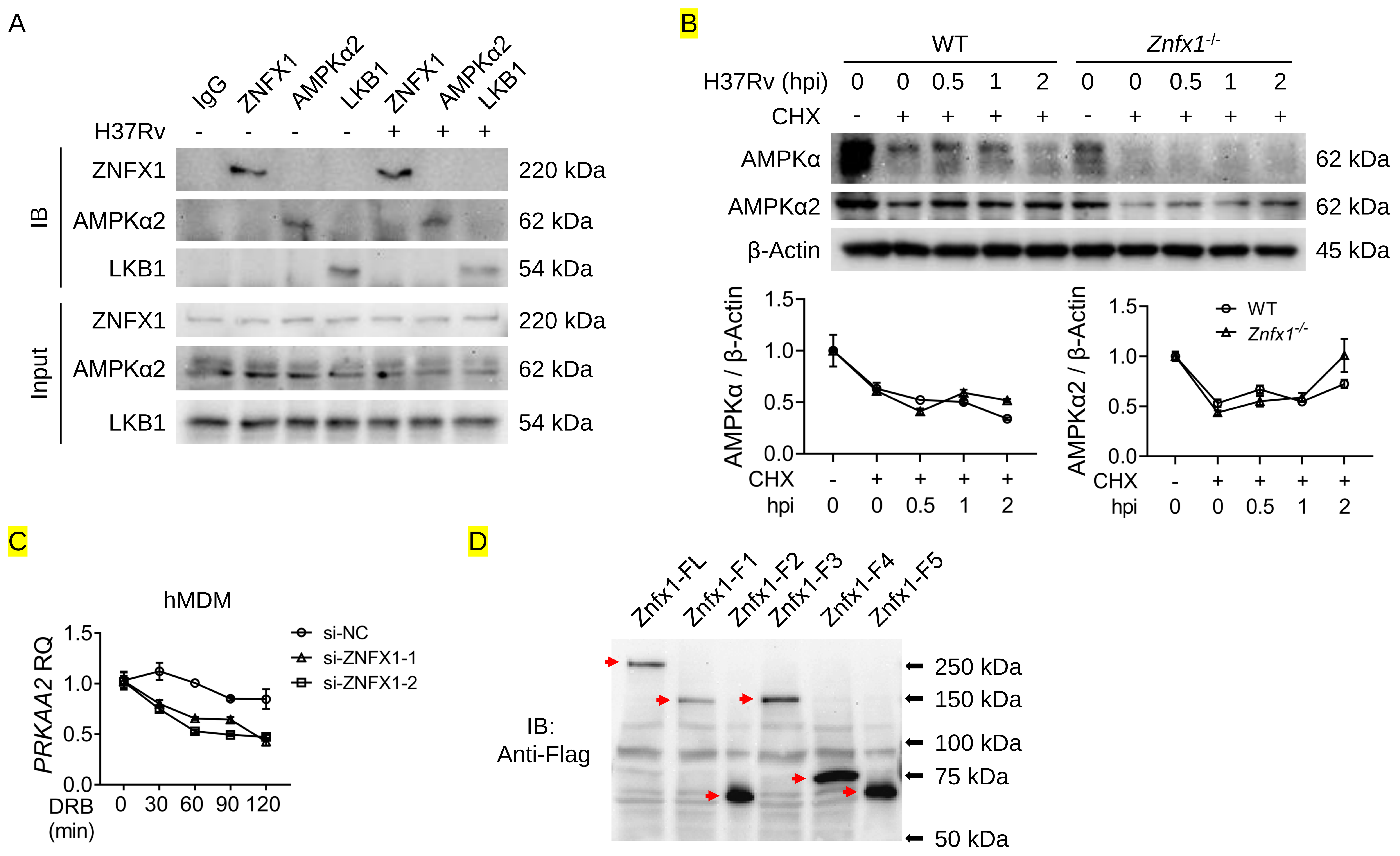

Supplementary Figure 7 for Figure 7. *In vitro* and *in vivo* experiments confirming that *Prkaa2* mediated the function of ZNFX1. (**A**) IP experiment and western blot assay of combination among ZNFX1, AMPKα2 and LKB1. (**B**) Western blotting of AMPKα and AMPKα2 in *Znfx1*$^{-/-}$ BMDMs treated with CHX, with analysis of protein degradation rates (n = 3). (**C**) qPCR analysis of *PRKAA2* in *ZNFX1*-silenced hMDMs treated with DRB (n = 3). (**D**) Western blot assay of full length or various truncated forms of ZNFX1 expression in 293T cells. A two-way ANOVA with Šidák's post hoc test (B and C) was used for statistical analysis. Data are presented as mean ± SD and are representative of at least three experiments with similar observations. *P < 0.05; **P < 0.01; ***P < 0.001; ****P < 0.0001.
