# Supplementary material for: ZNFX1 promotes AMPK-mediated autophagy against Mycobacterium tuberculosis by stabilizing Prkaa2 mRNA
Source: JCI Insight. 2024 Jan 9;9(1):e171850. doi: 10.1172/jci.insight.171850 (PMC10906457; doi:10.1172/jci.insight.171850)

Original data of western blotting

Full unedited gel for Figure 1C

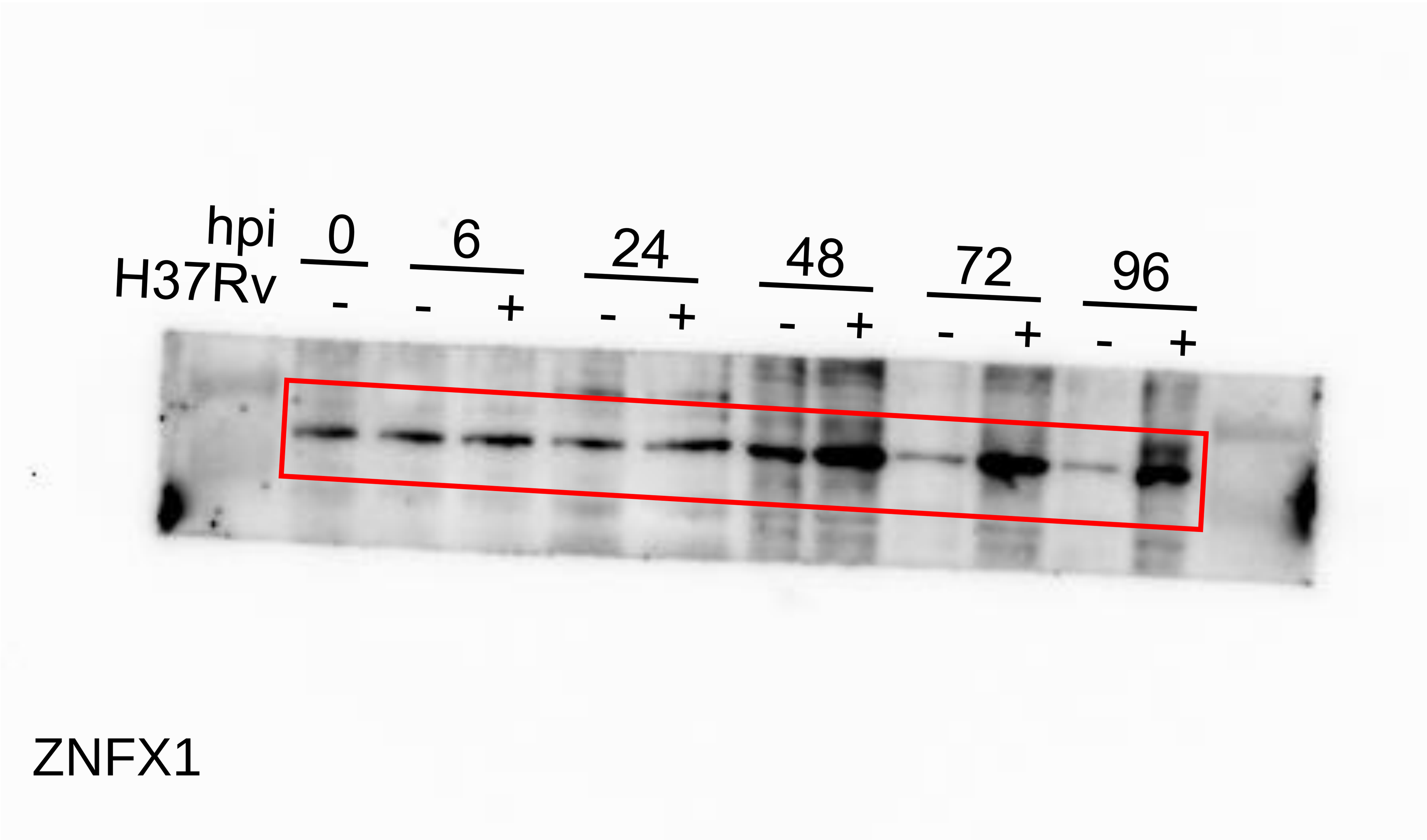

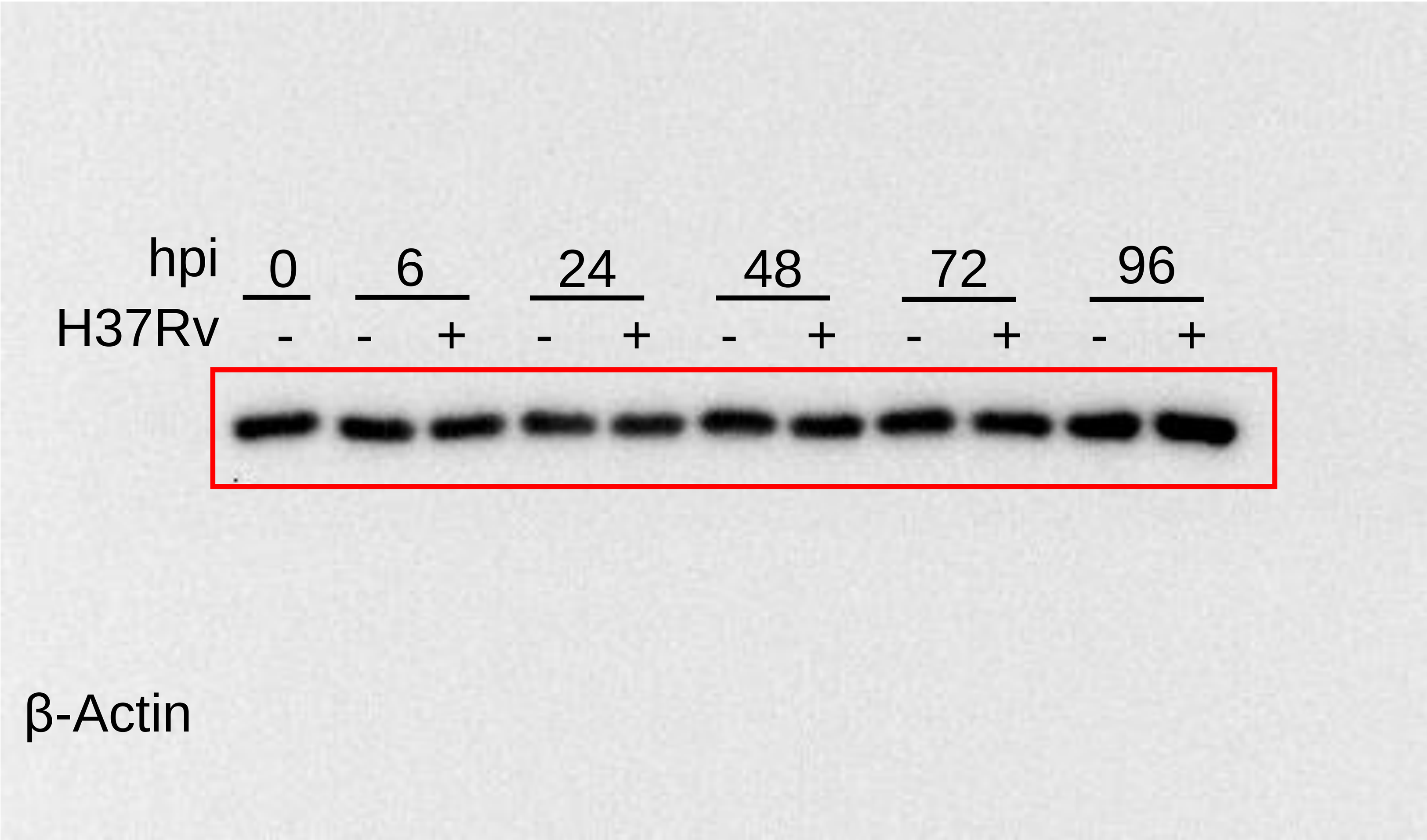

Full unedited gel for Figure 3E

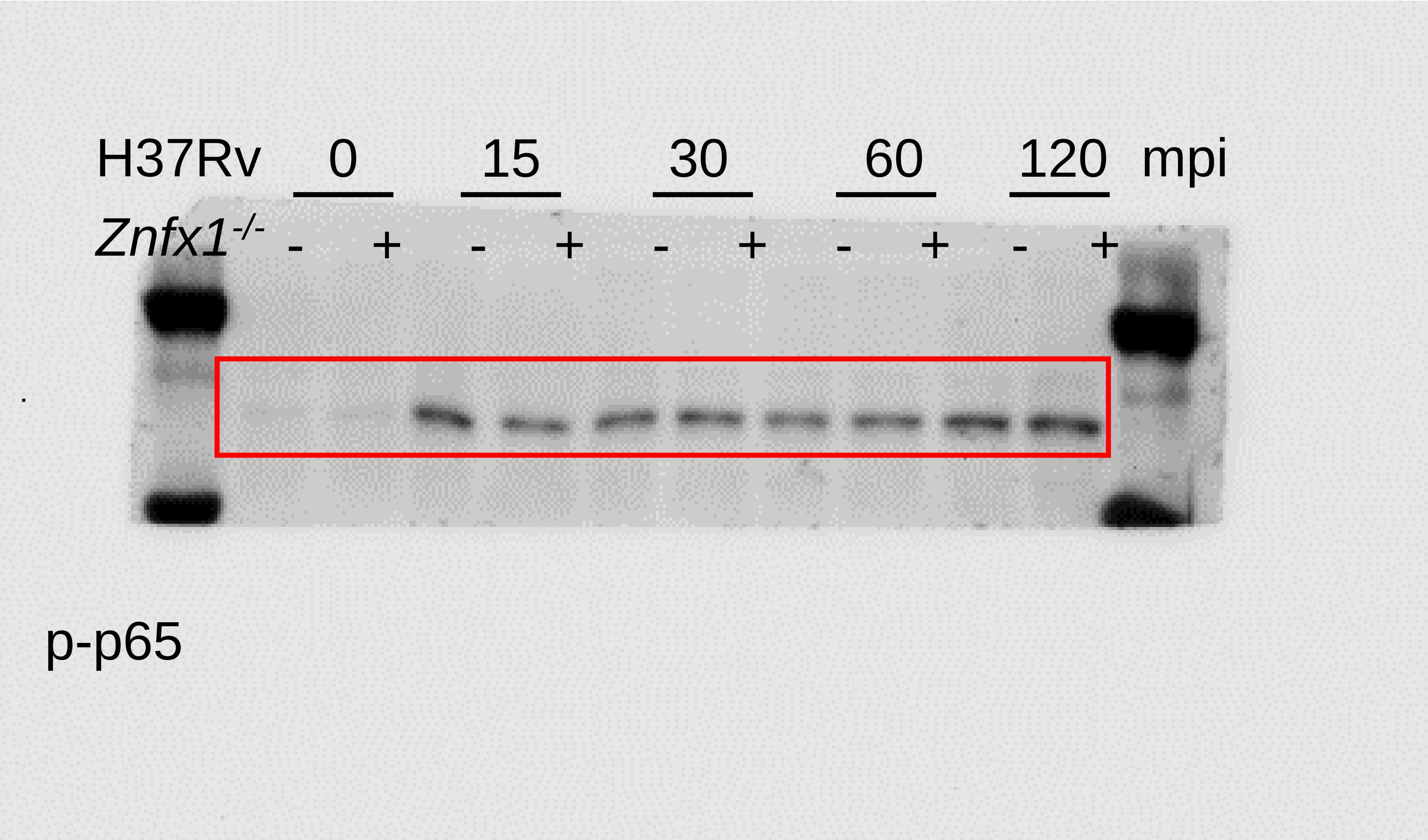

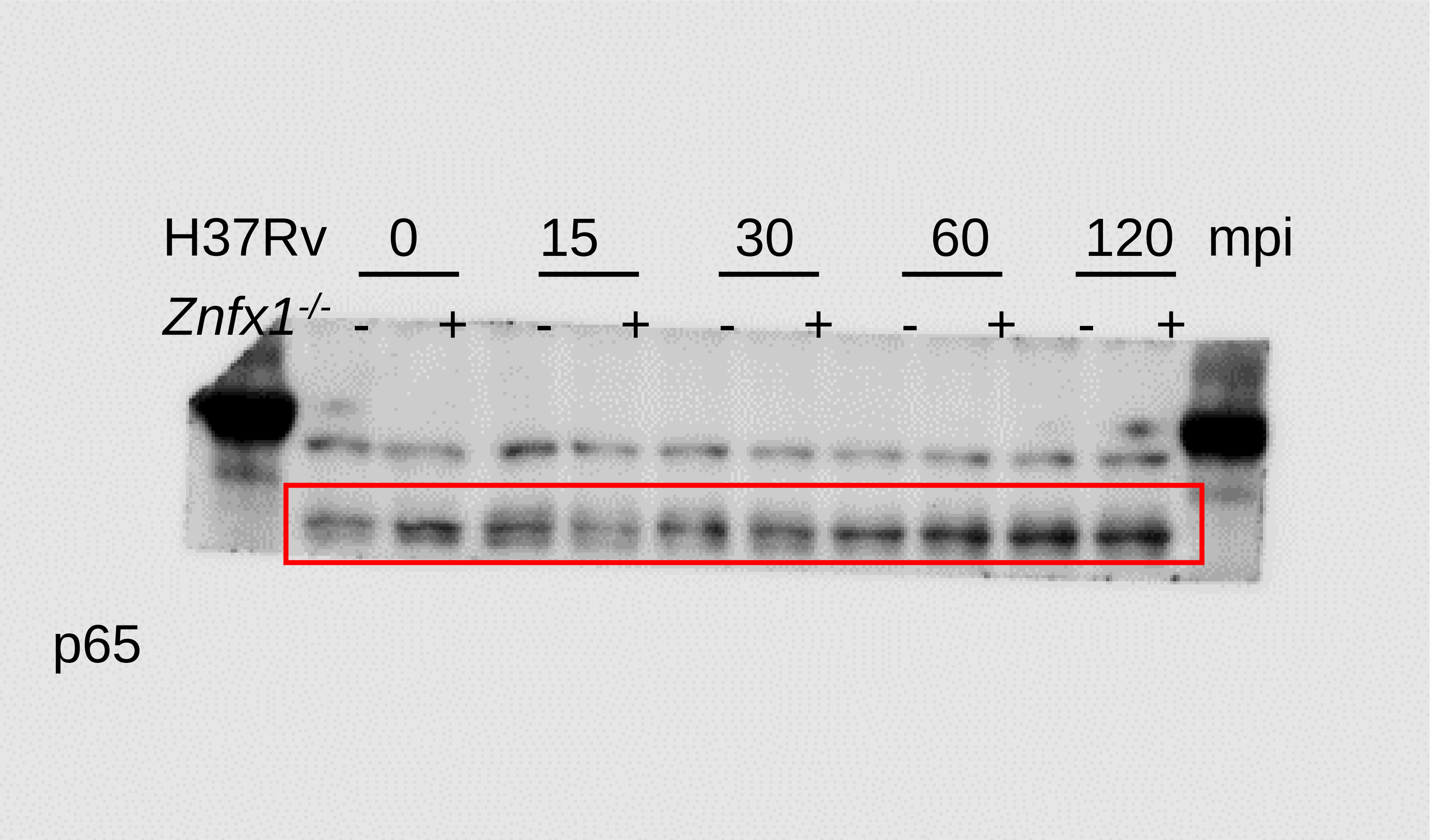

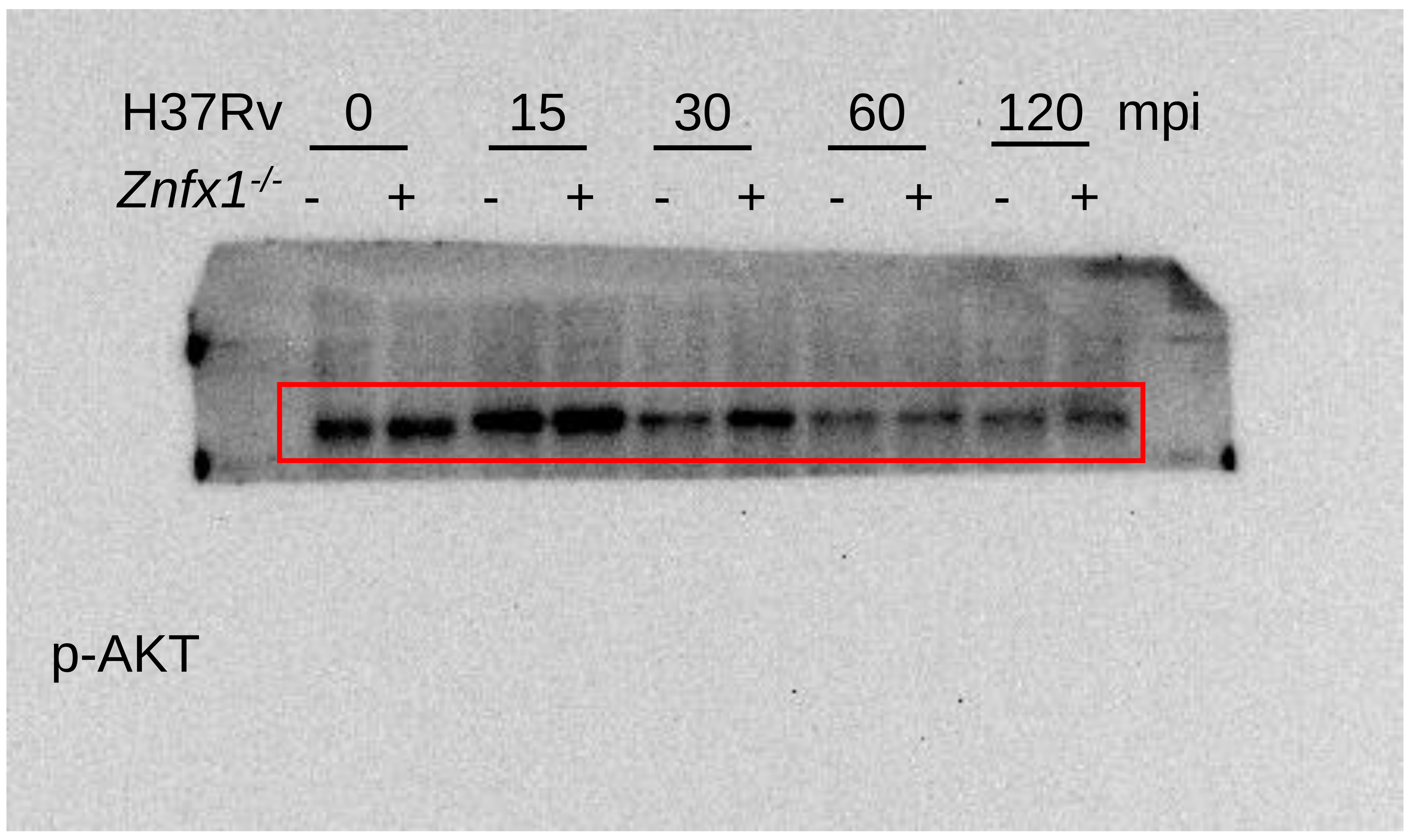

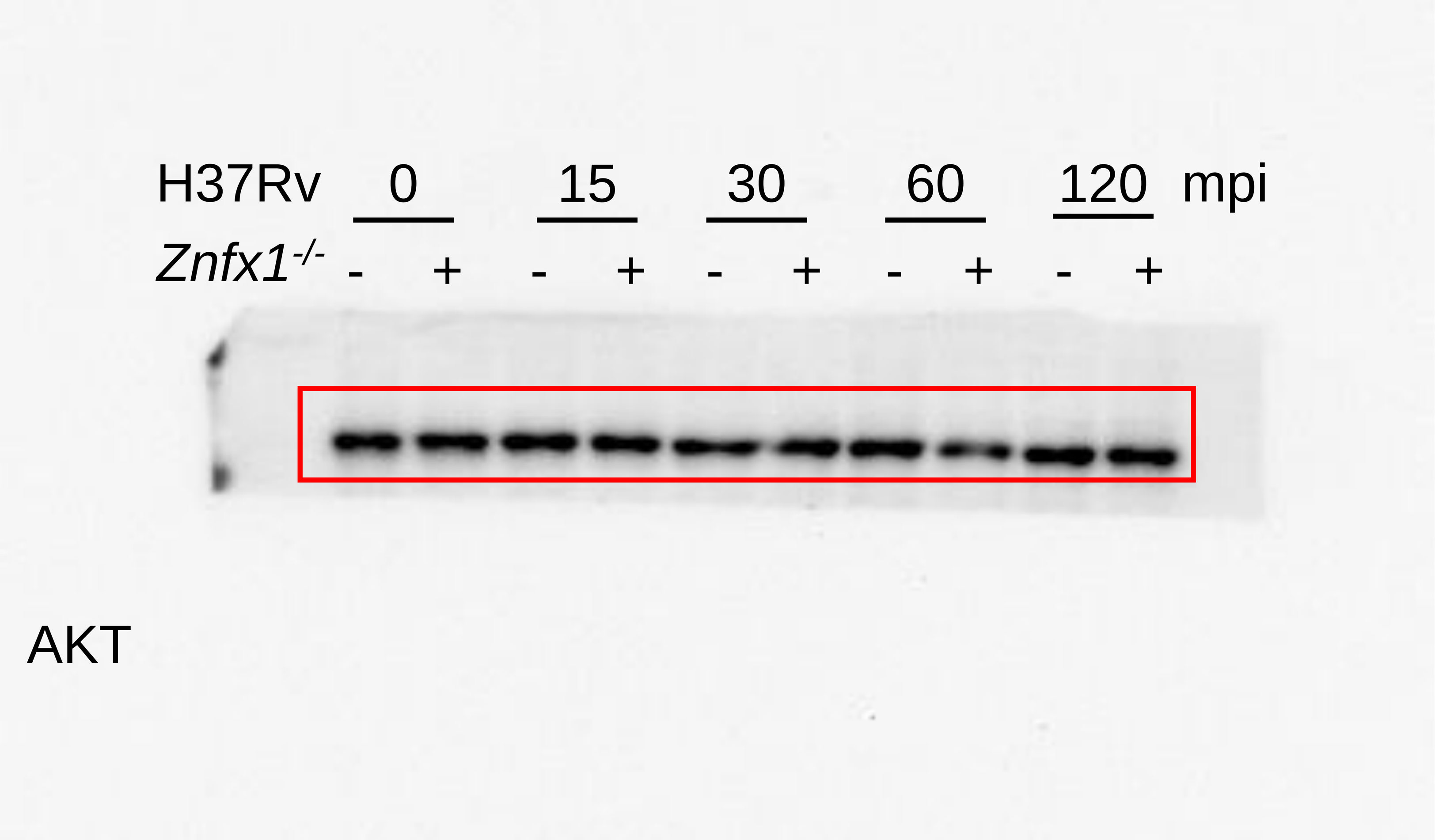

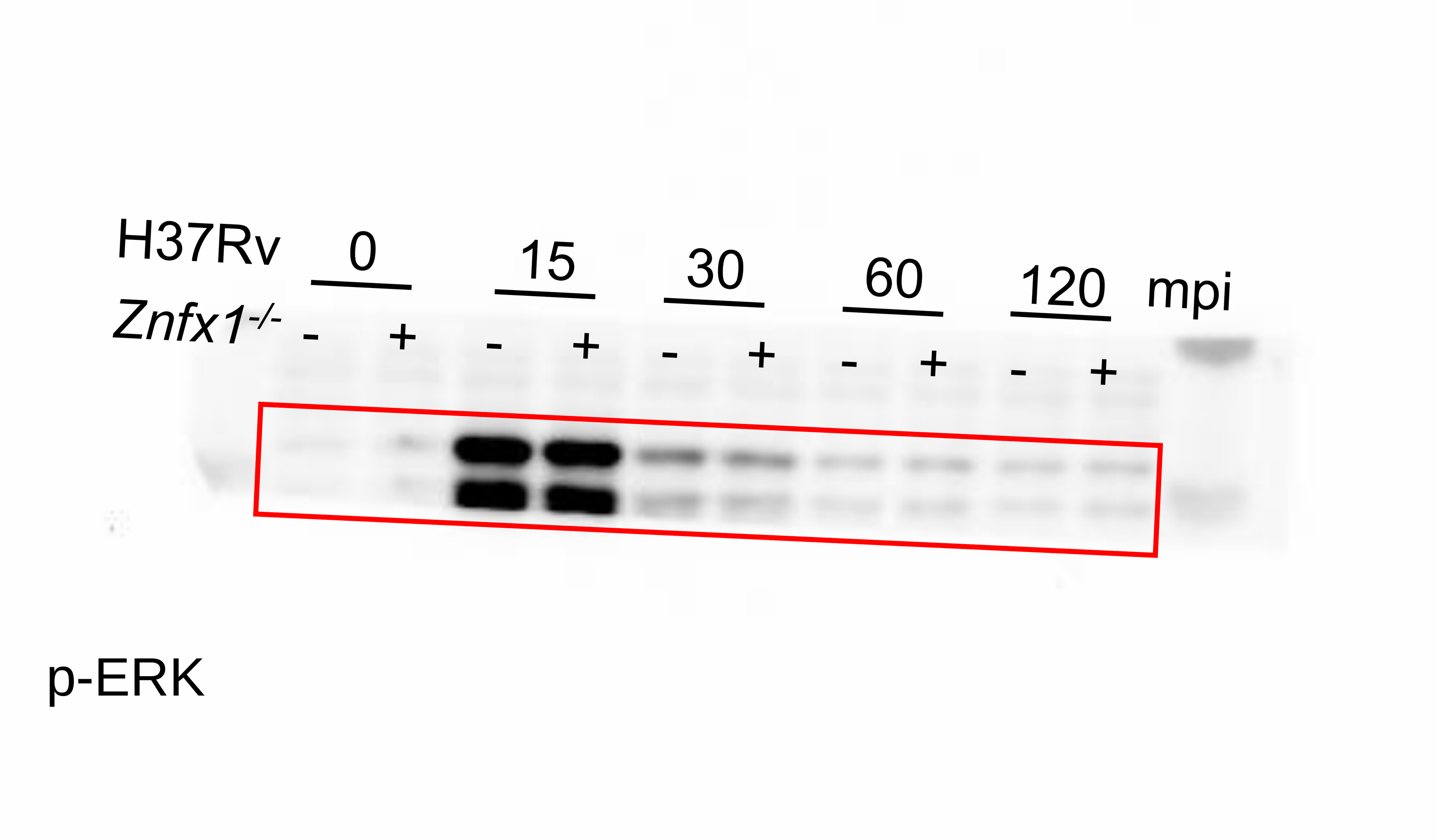

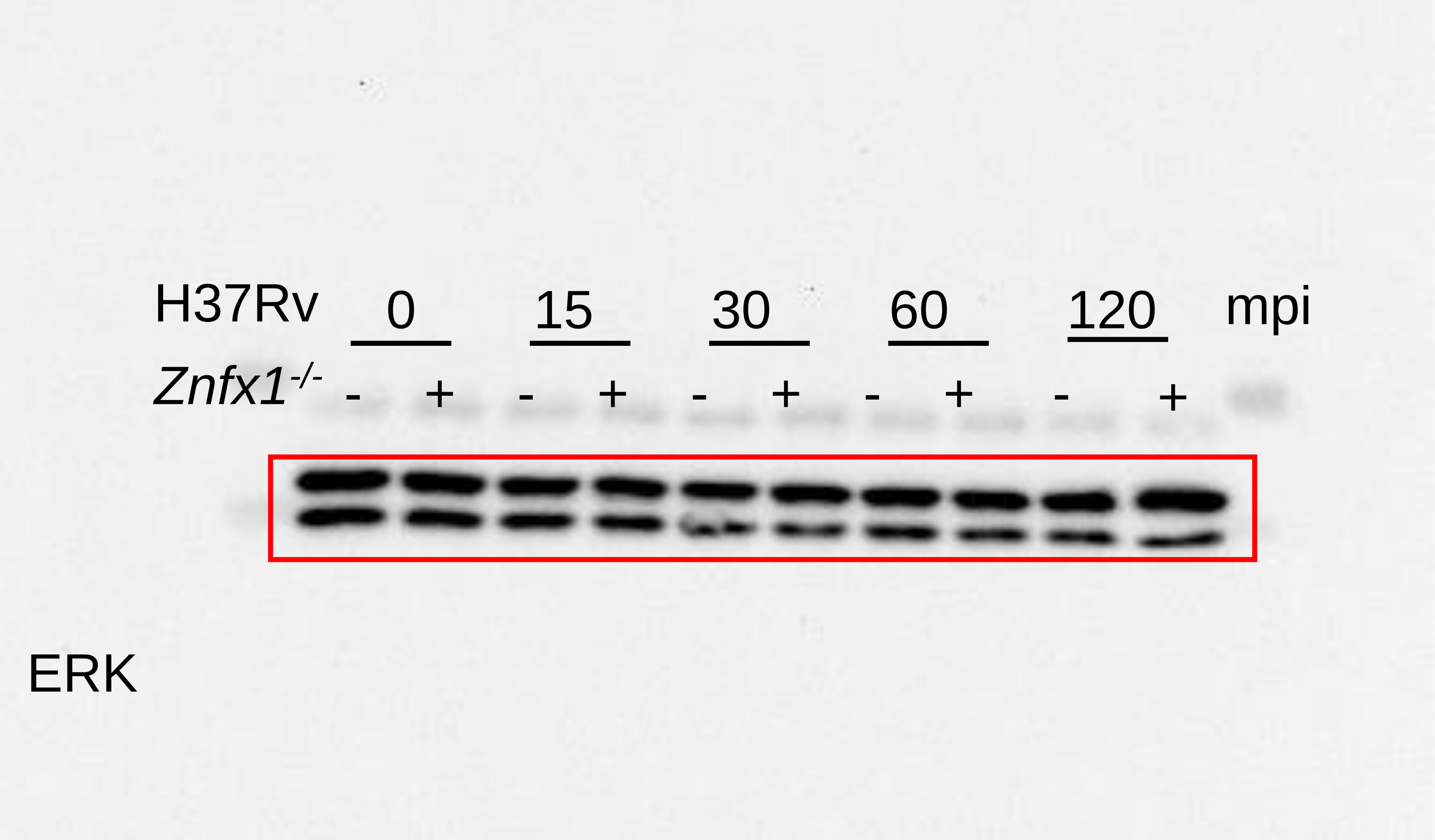

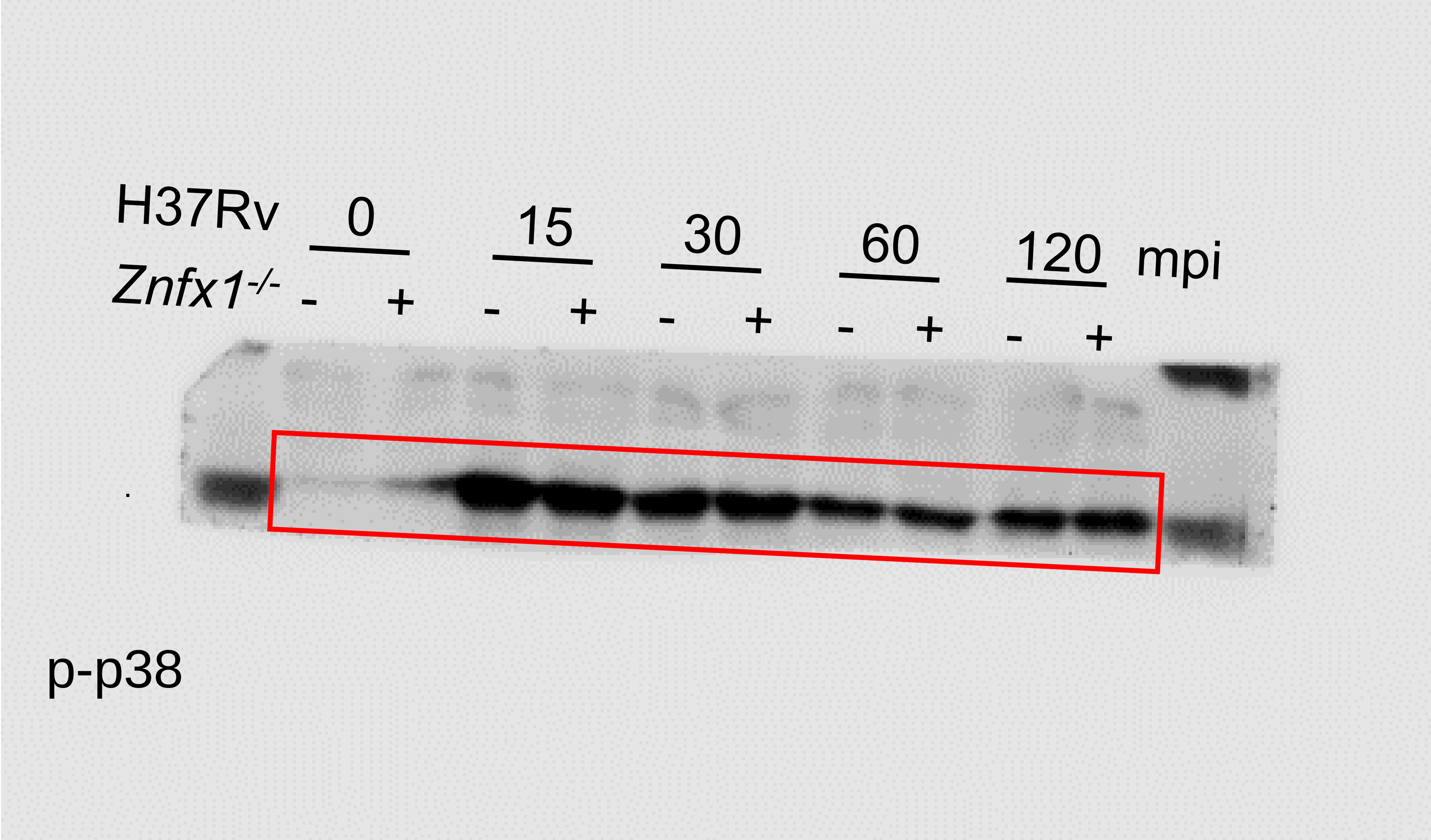

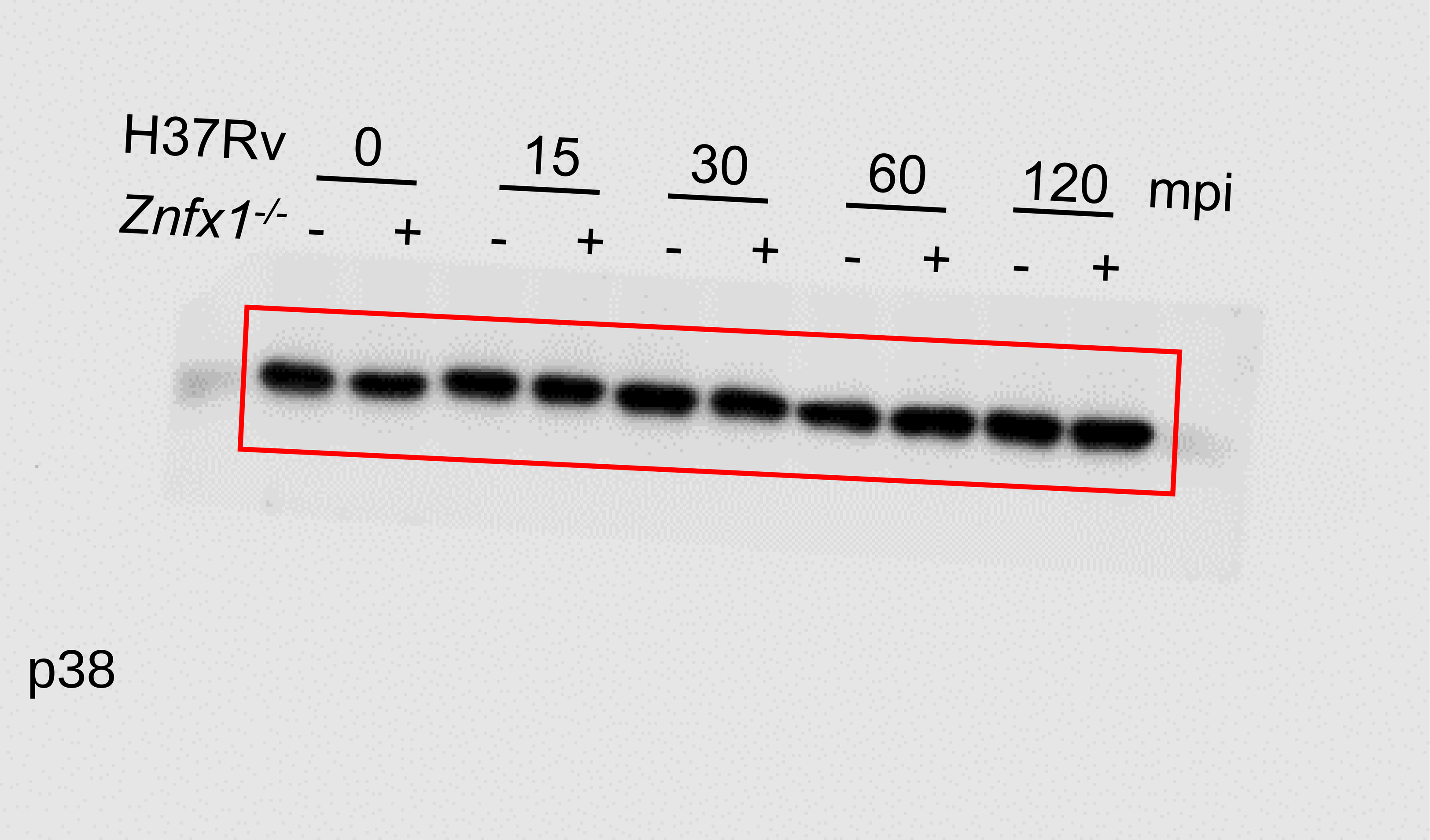

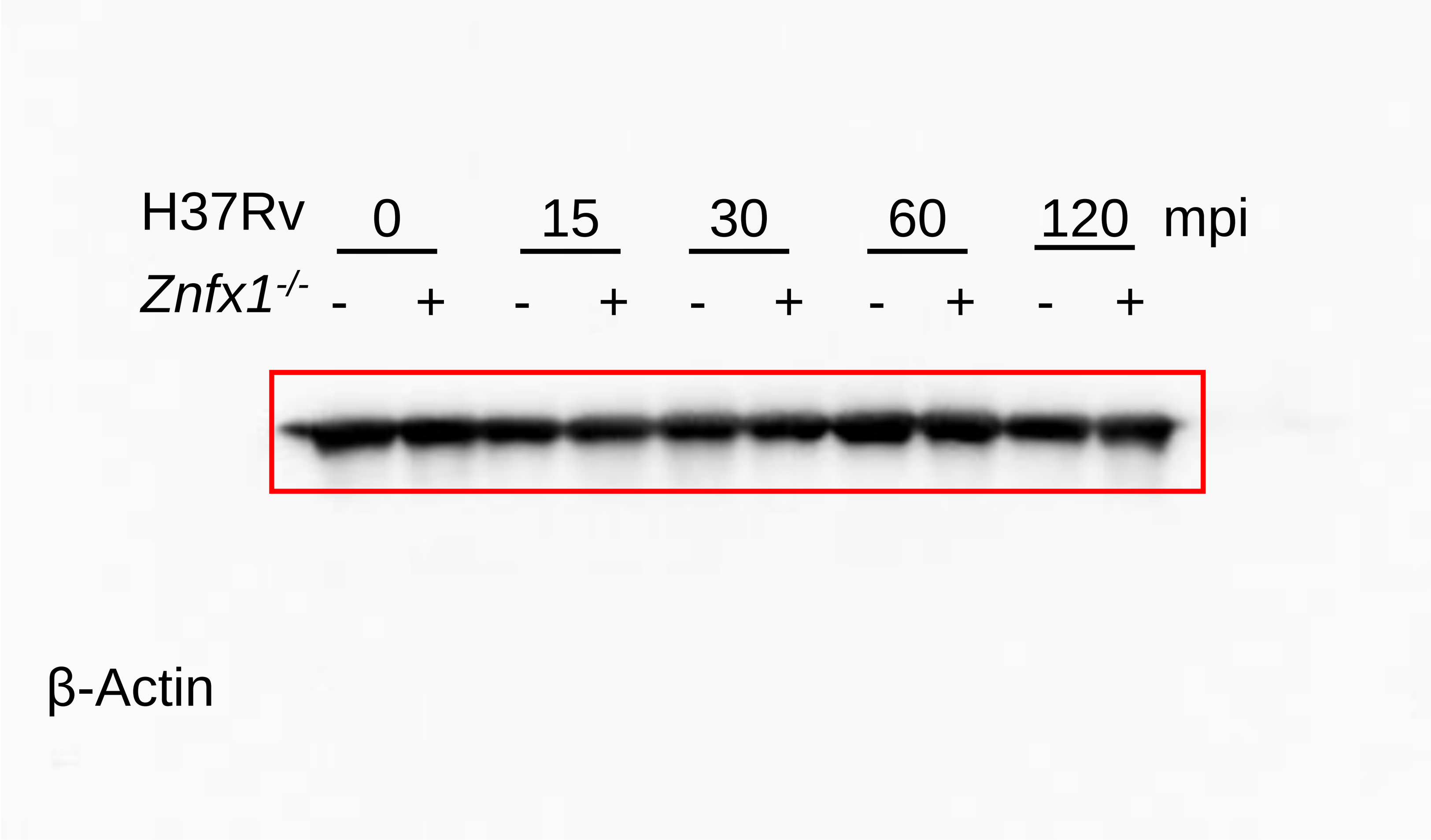

Full unedited gel for Figure 3F

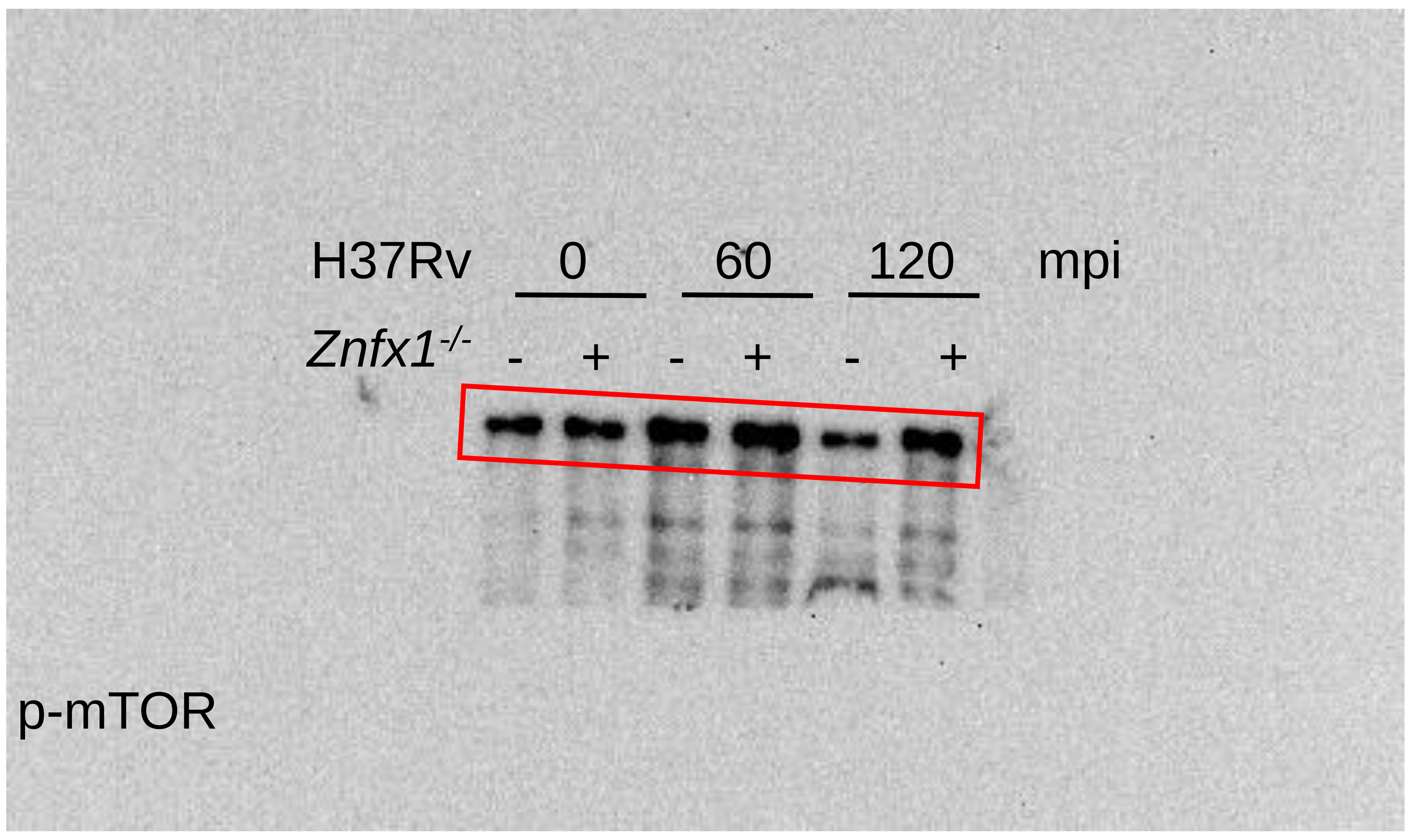

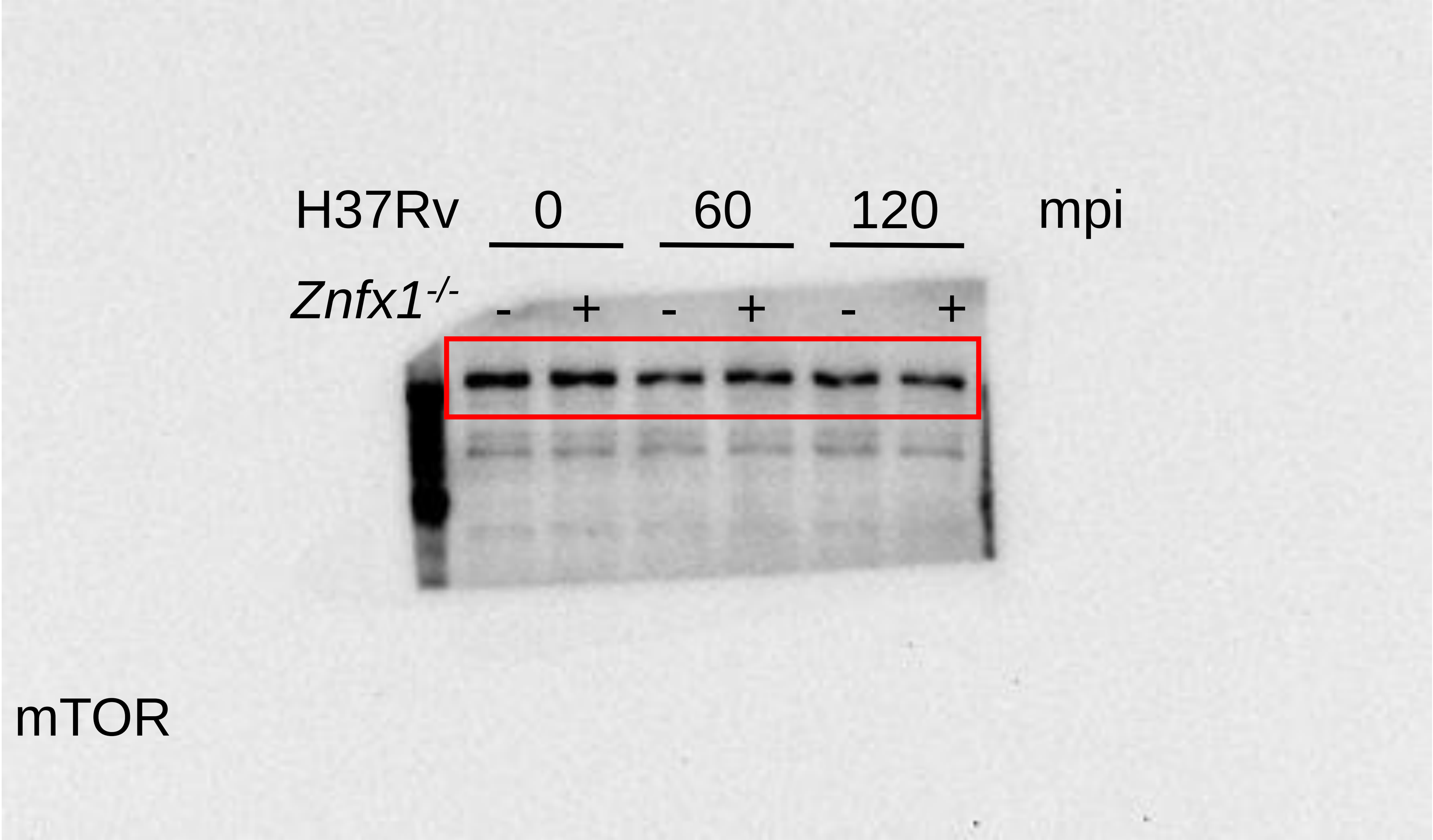

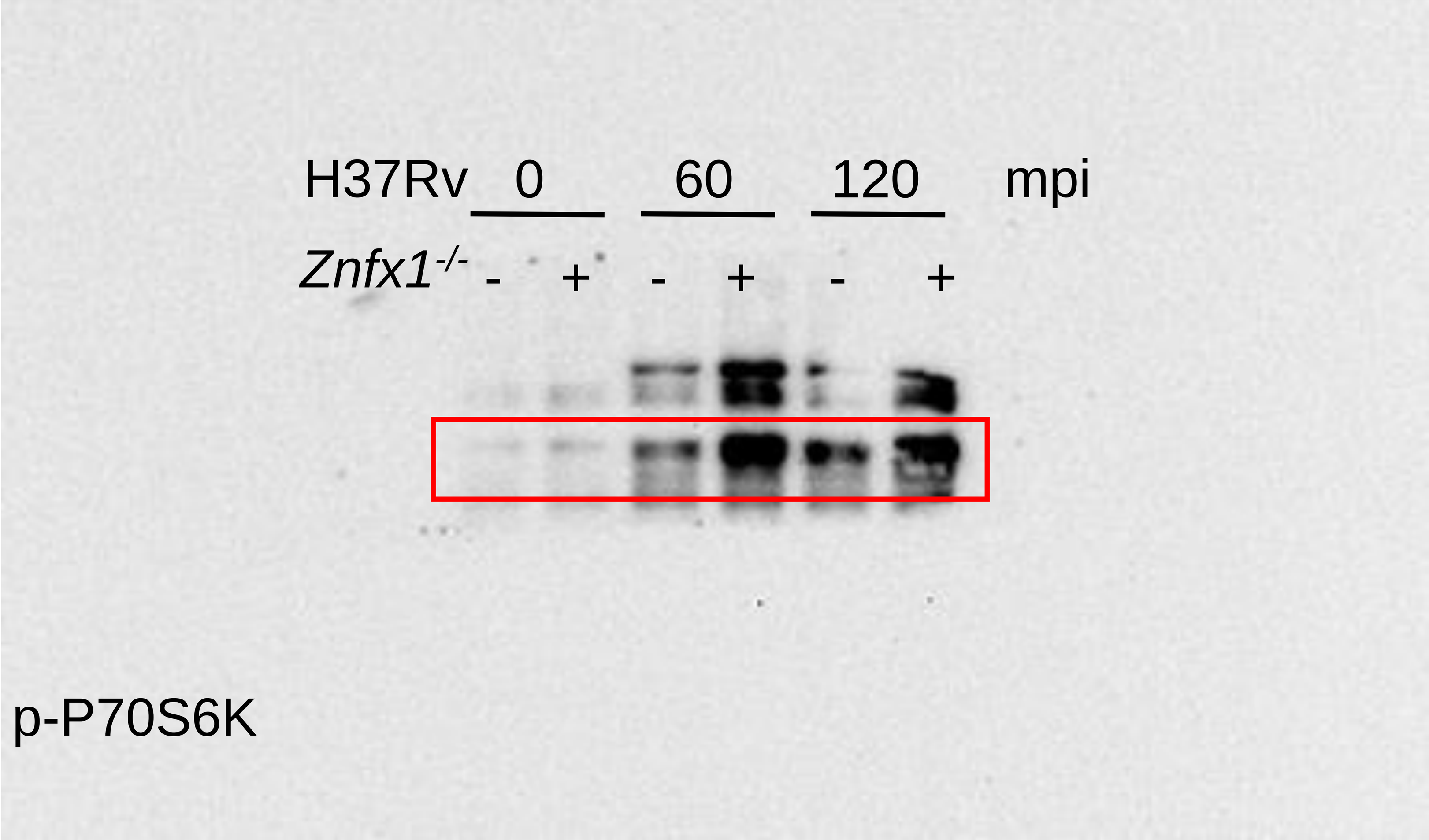

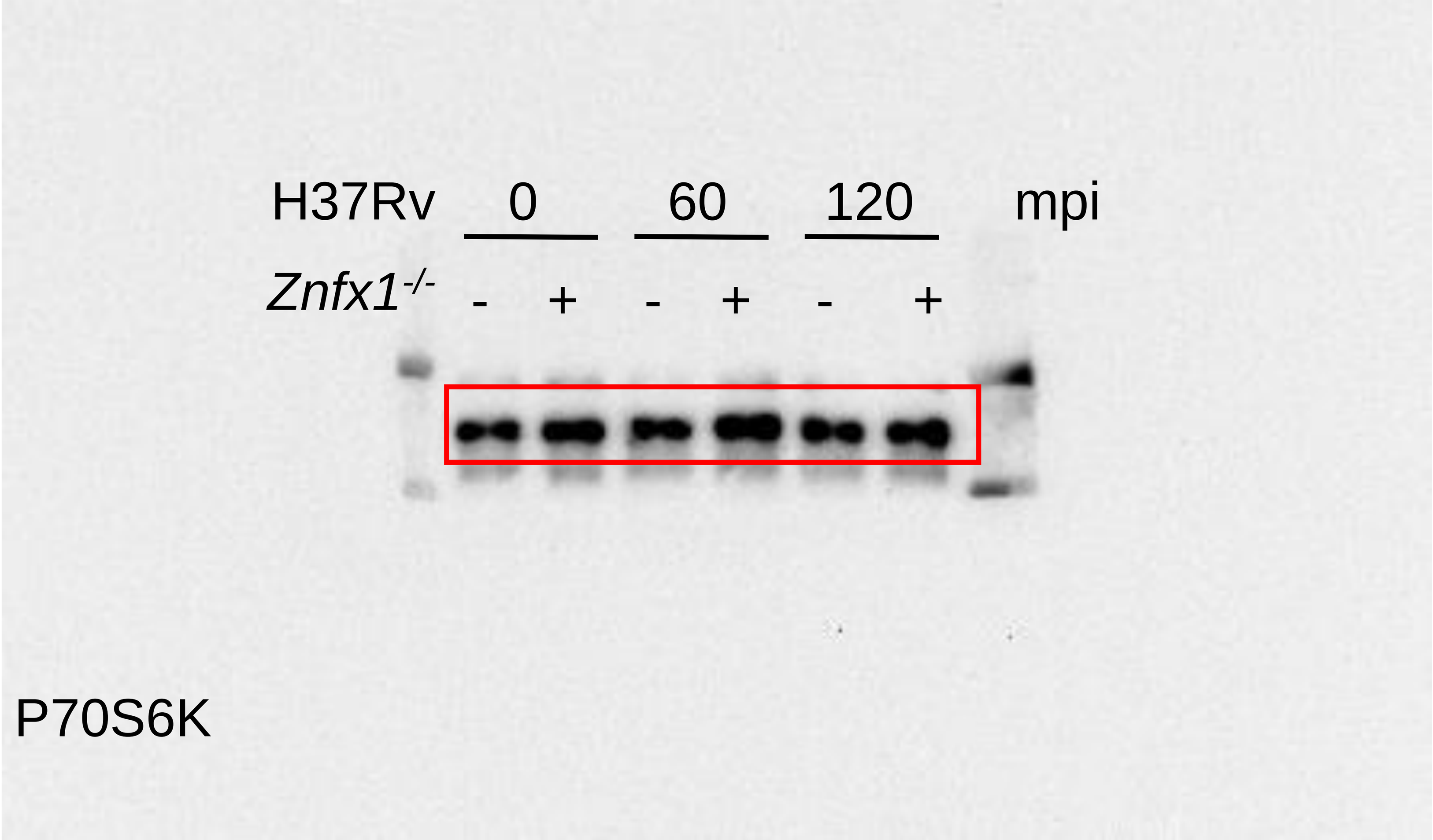

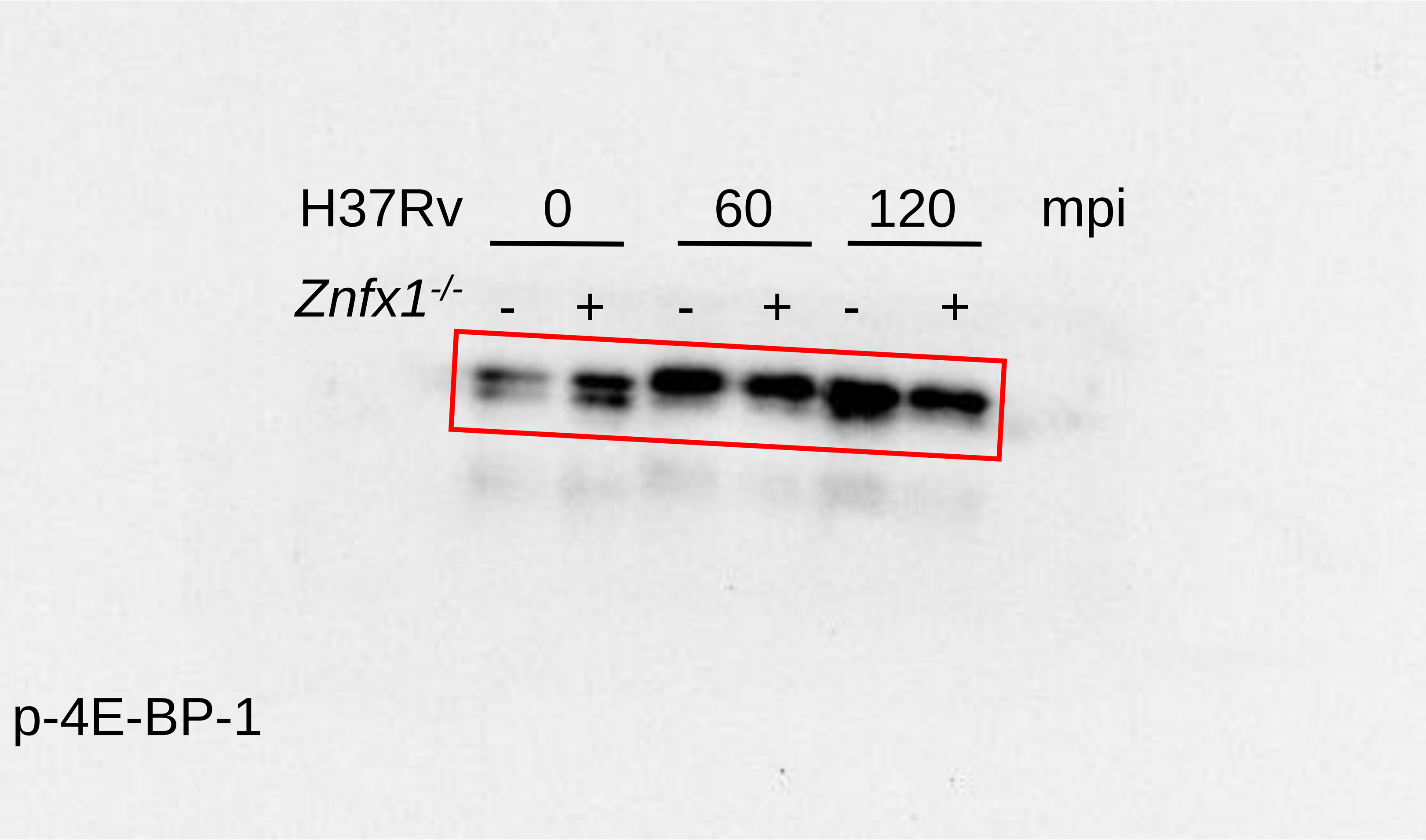

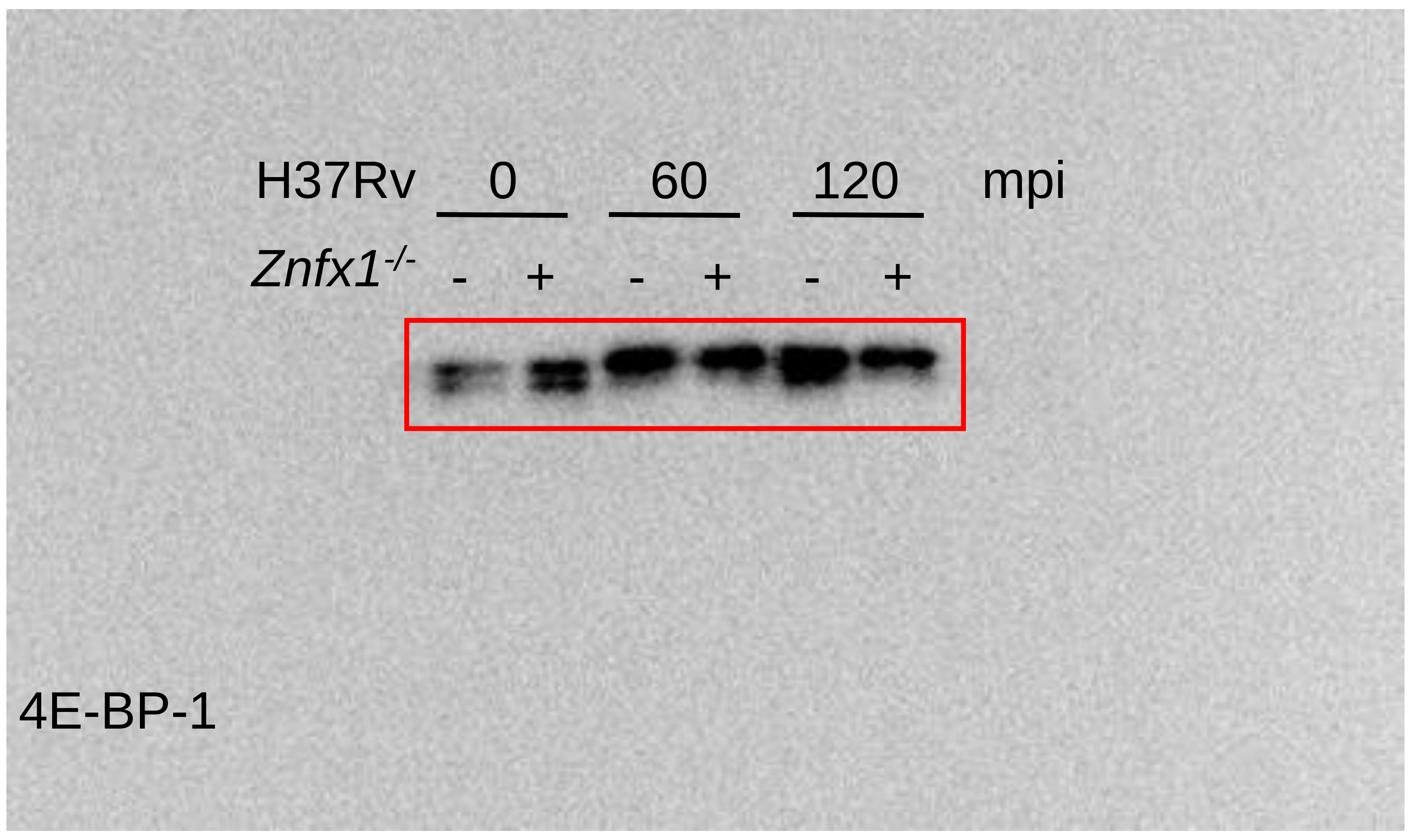

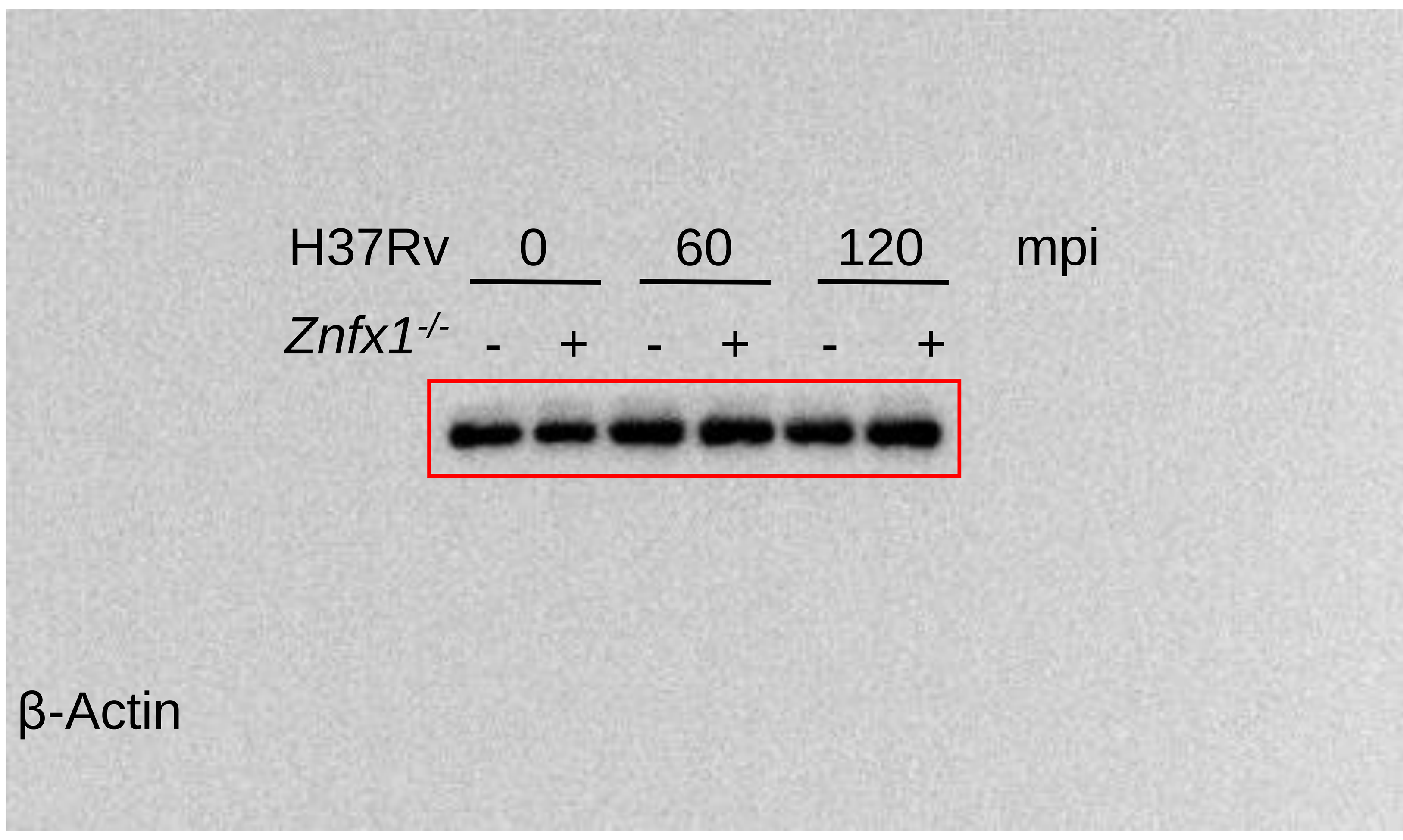

Full unedited gel for Figure 4A

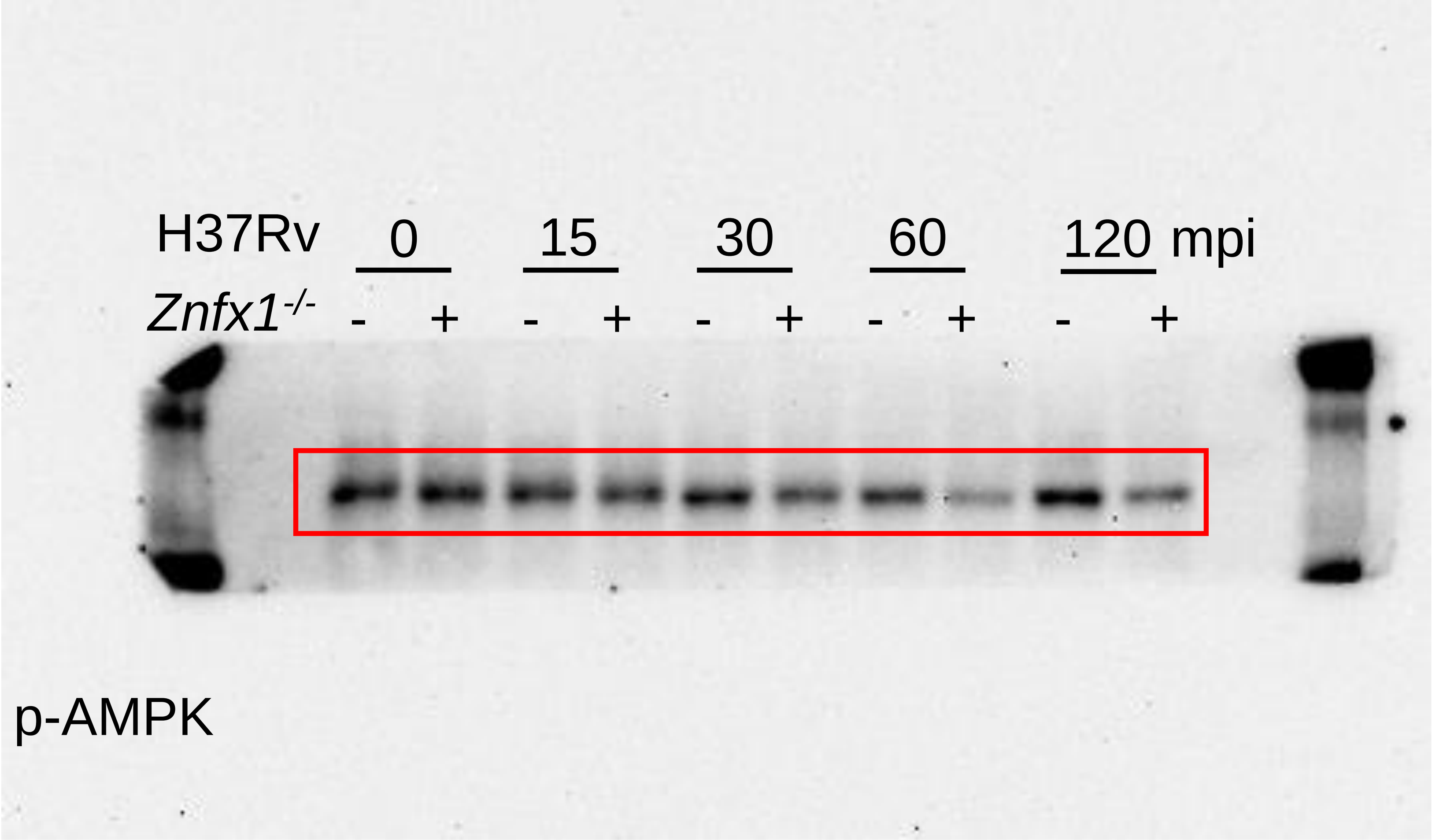

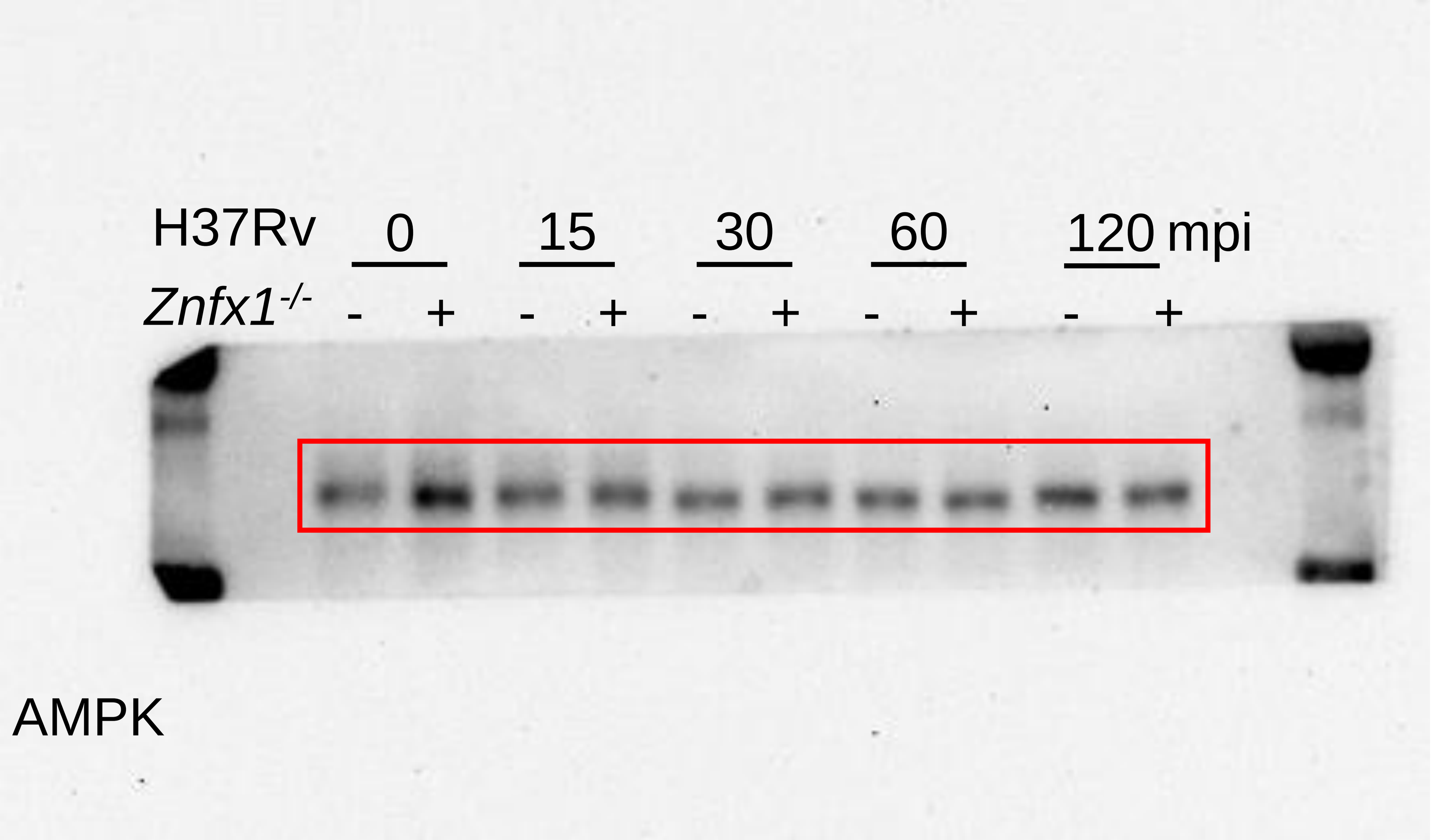

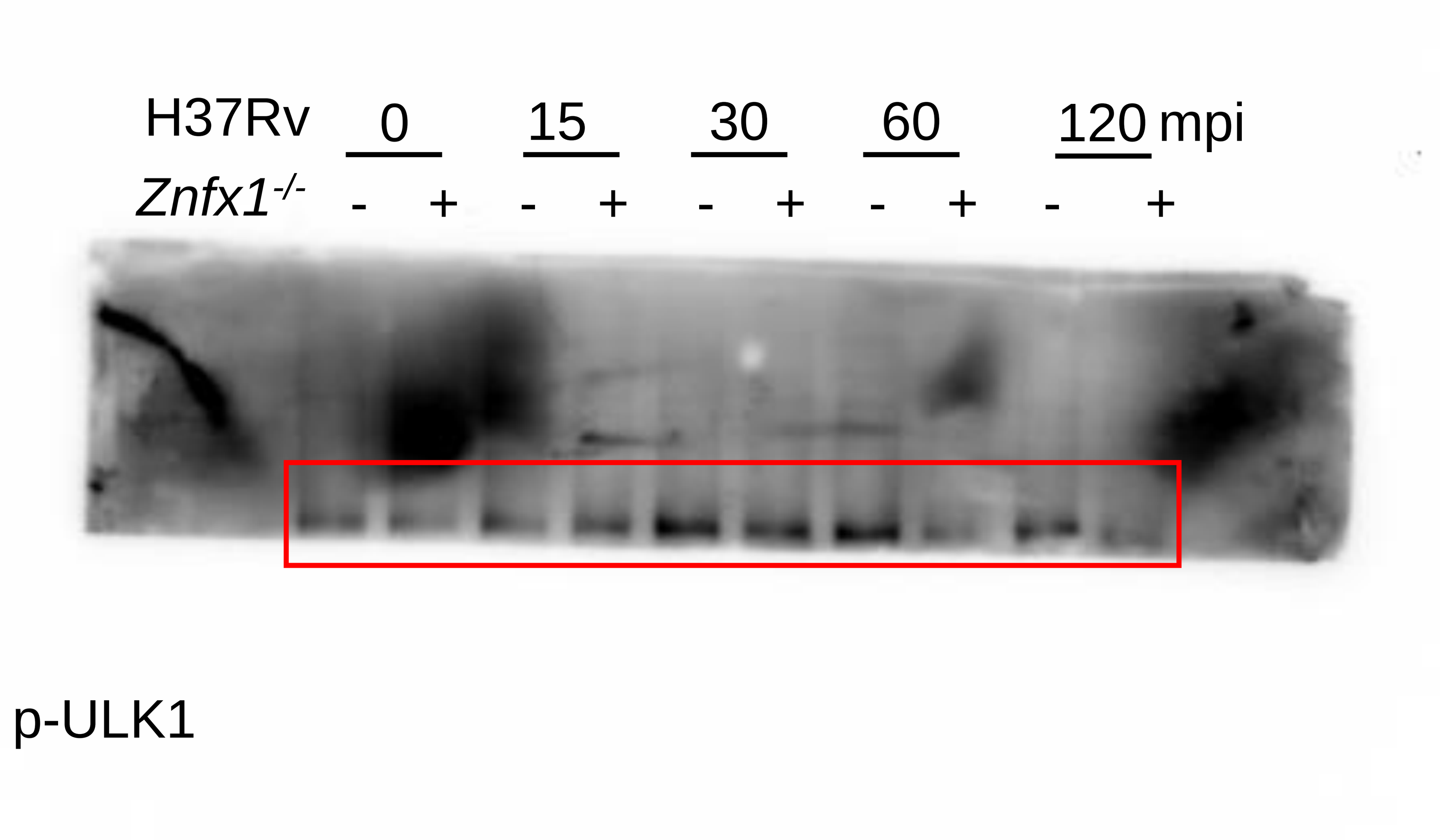

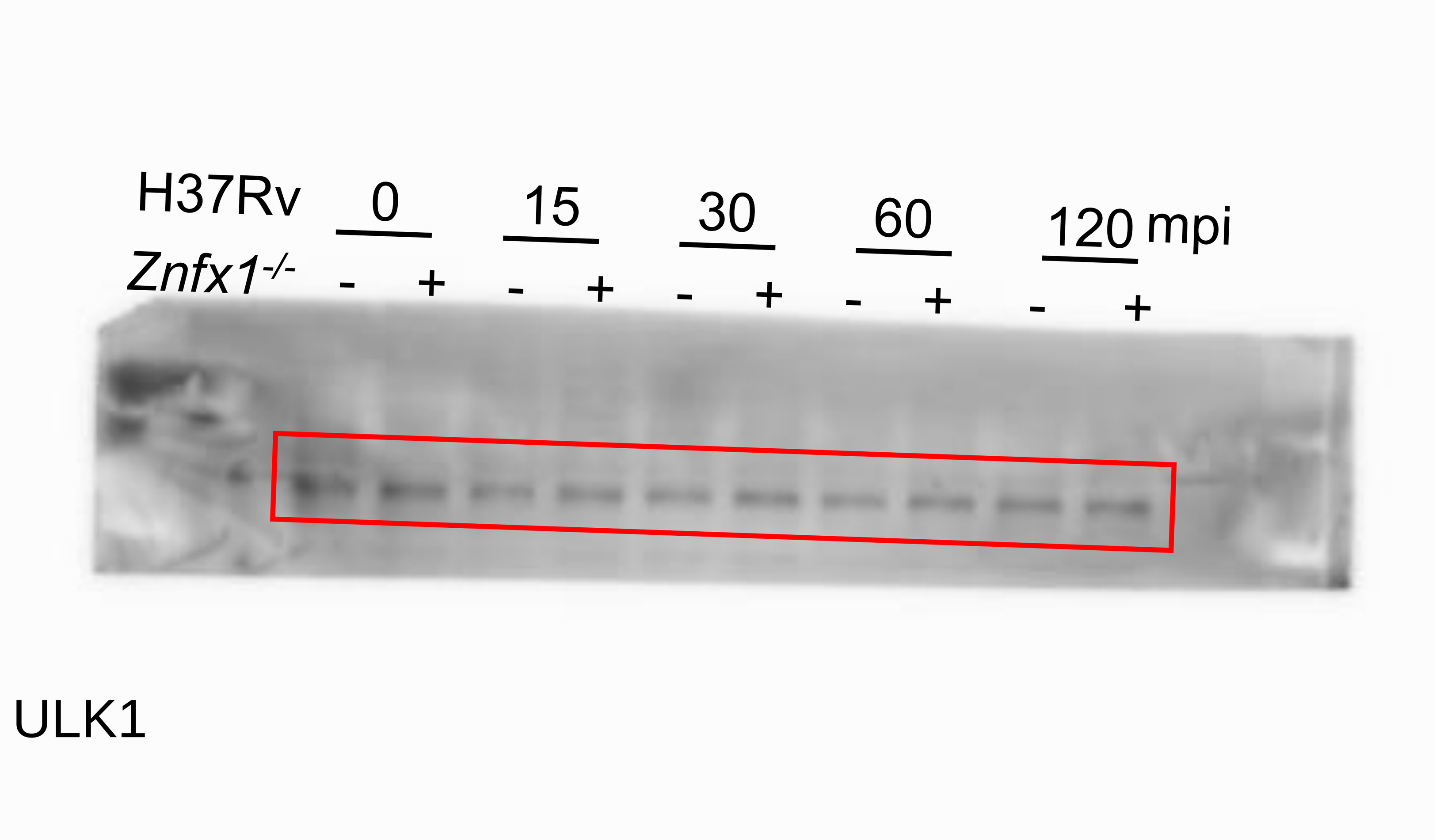

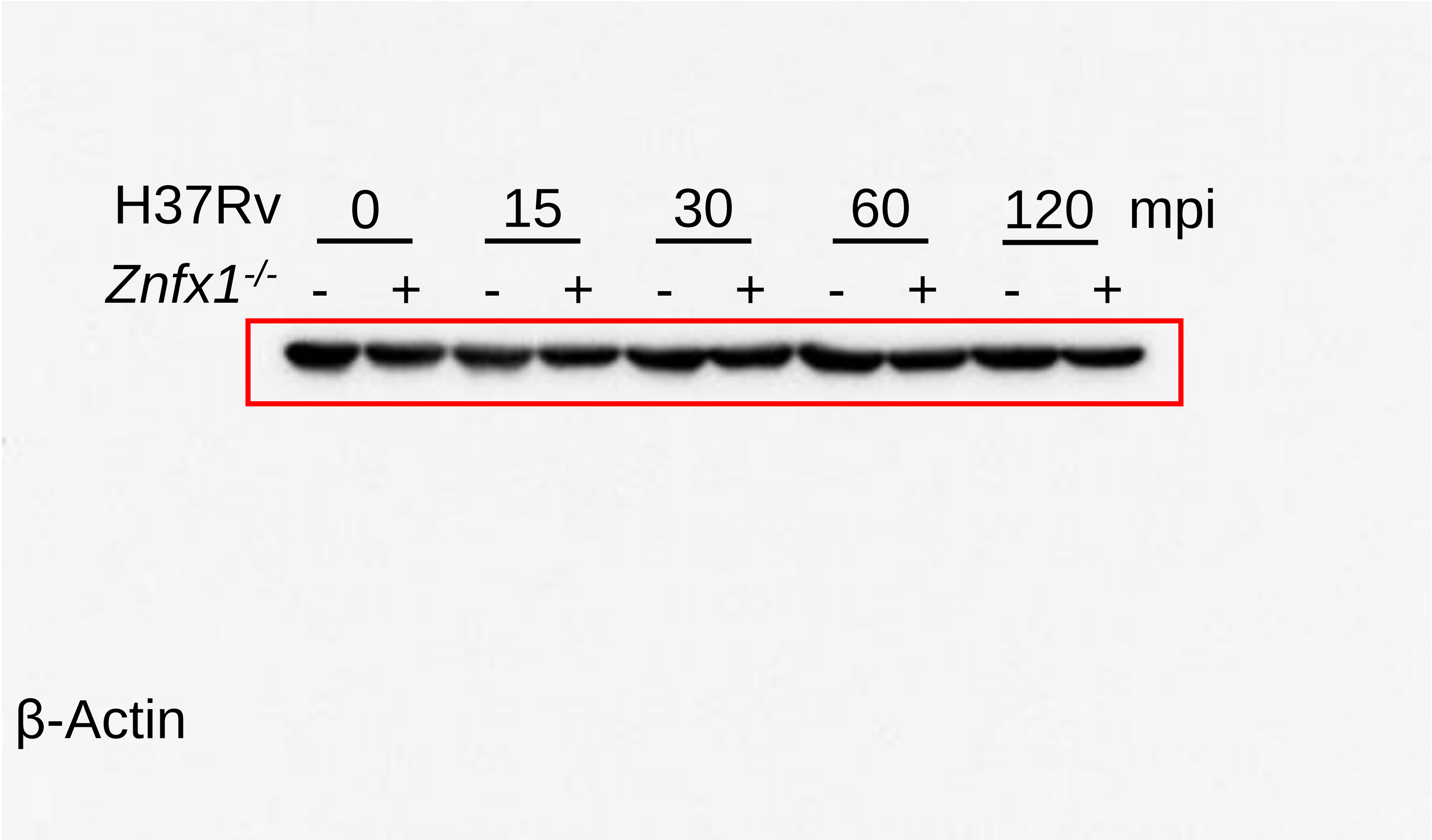

Full unedited gel for Figure 4B

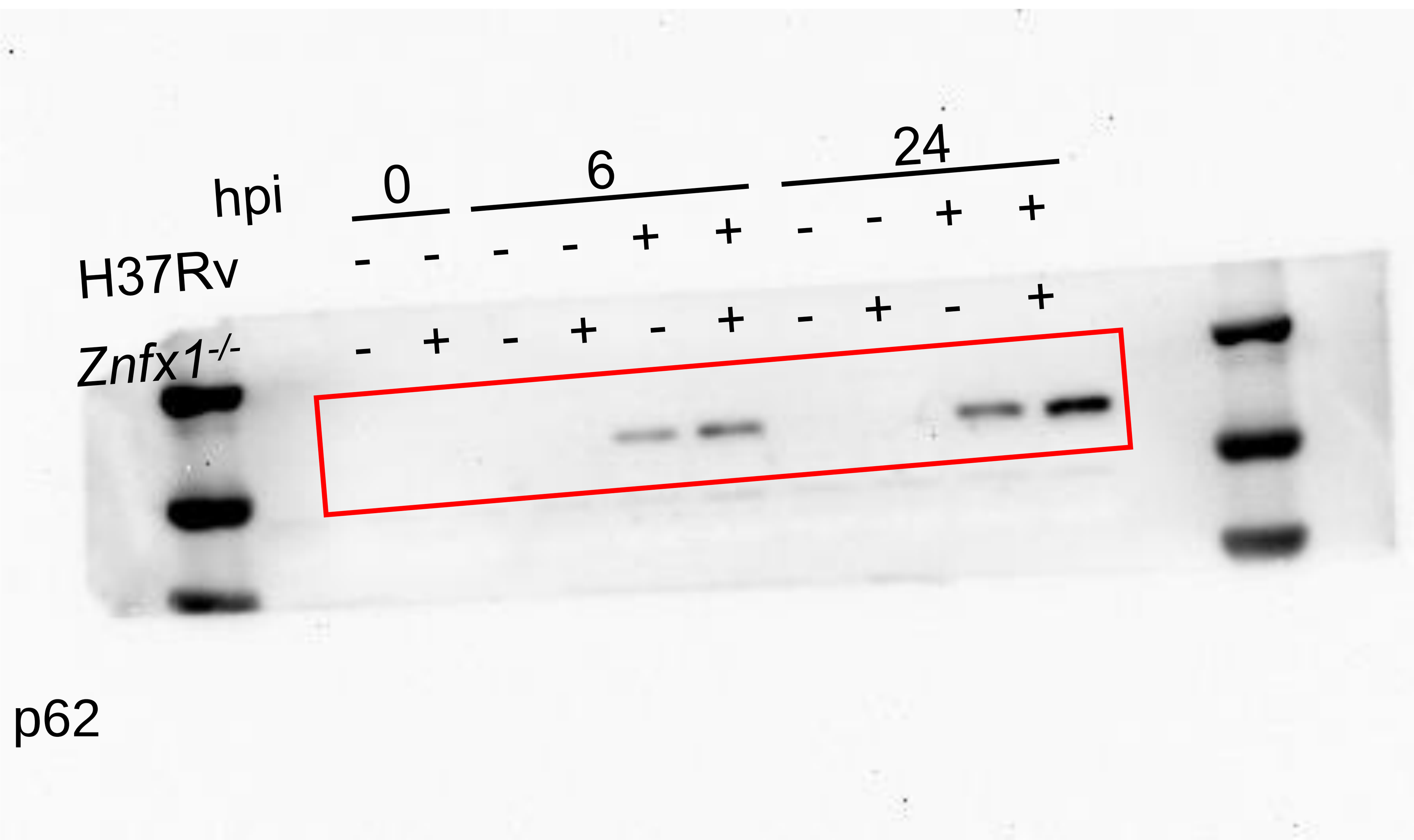

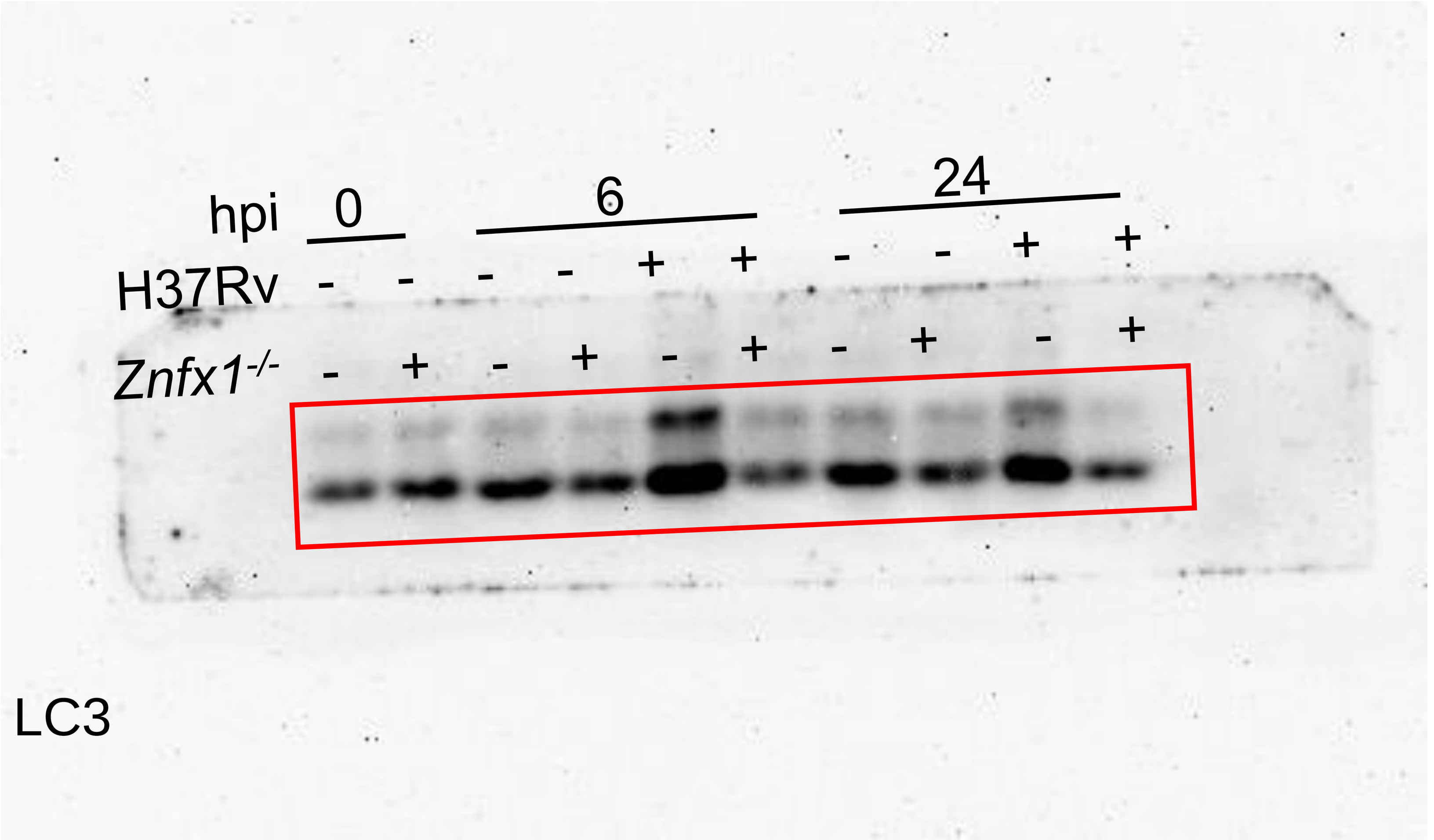

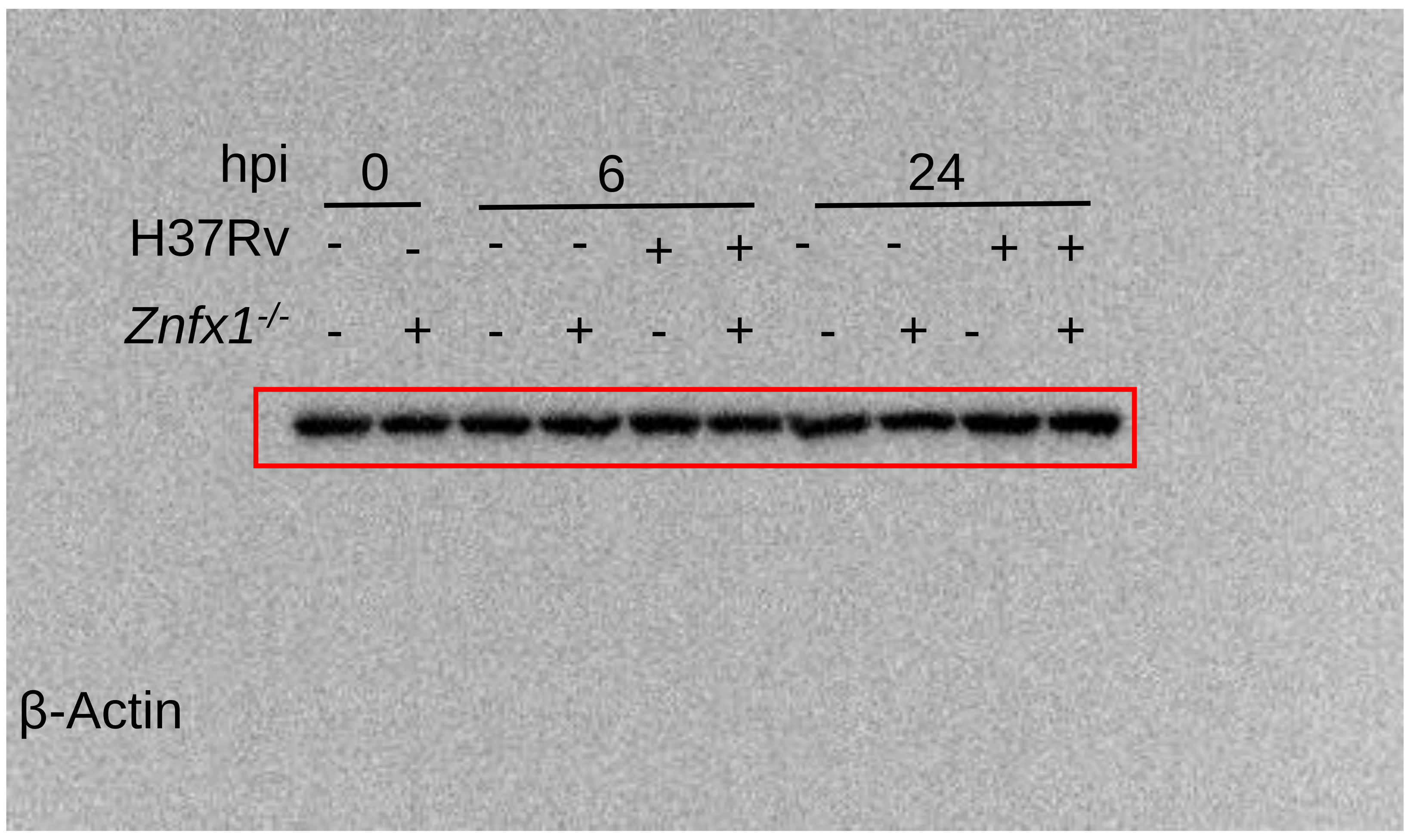

Full unedited gel for Figure 5C

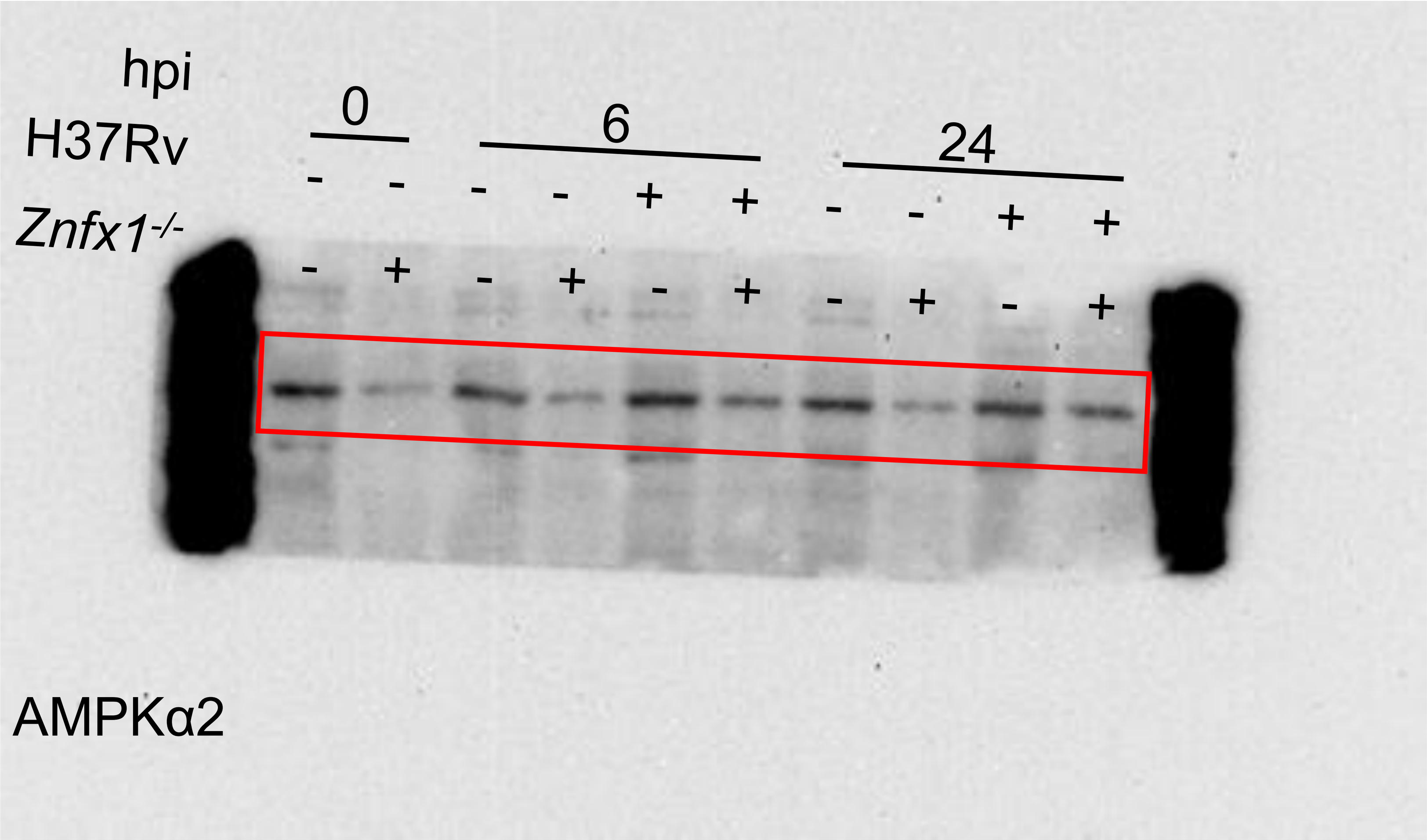

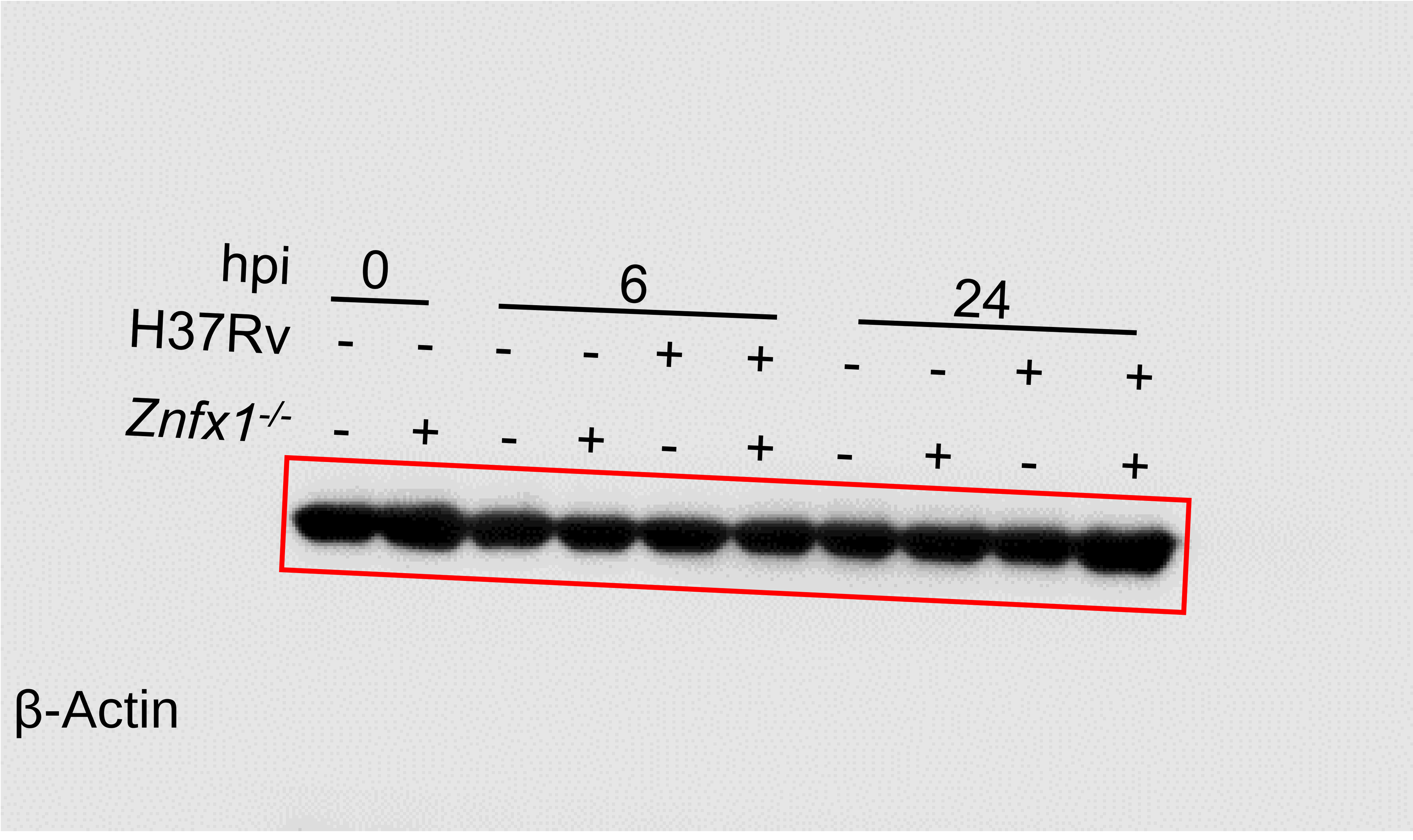

Full unedited gel for Figure 7A

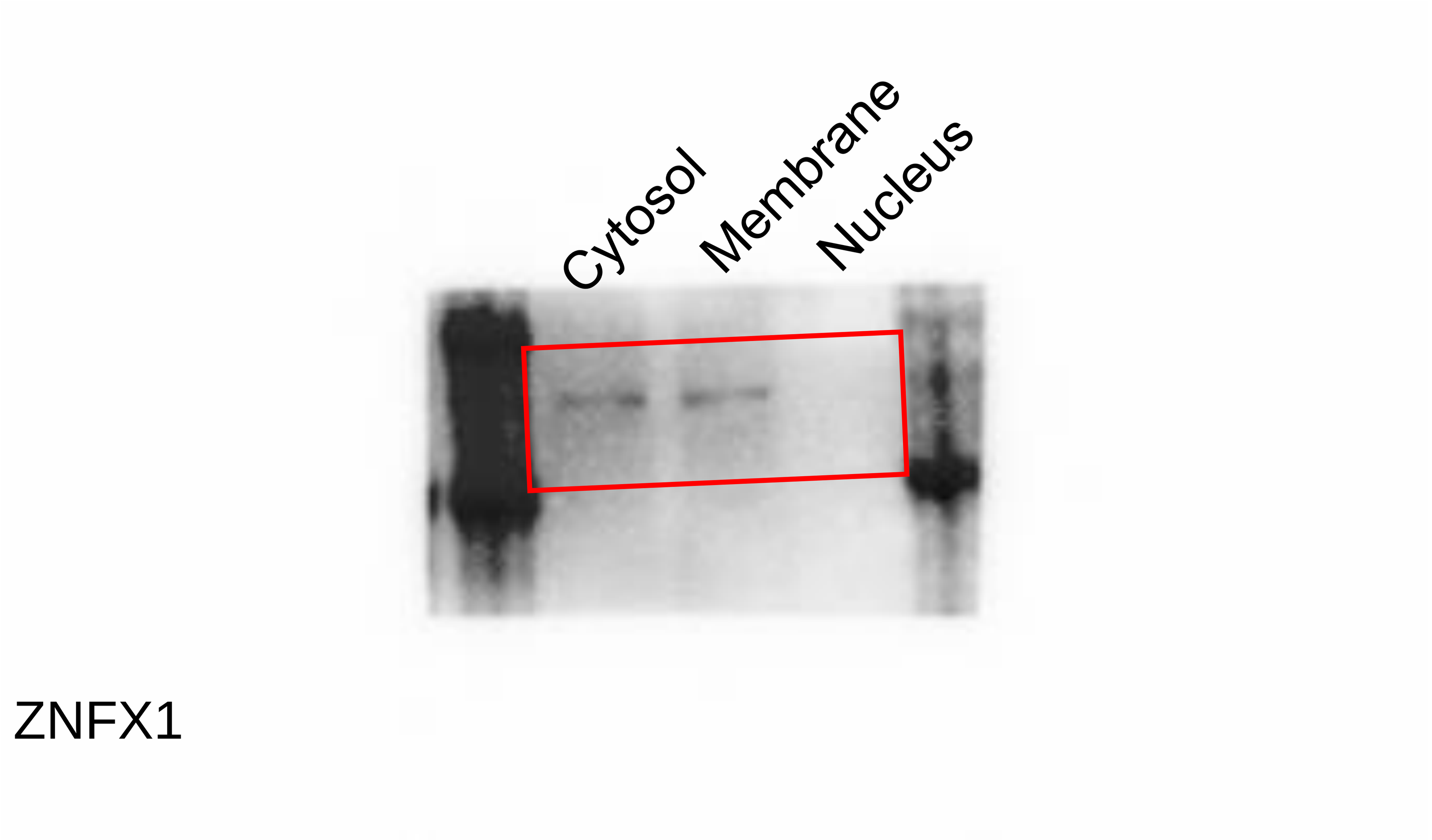

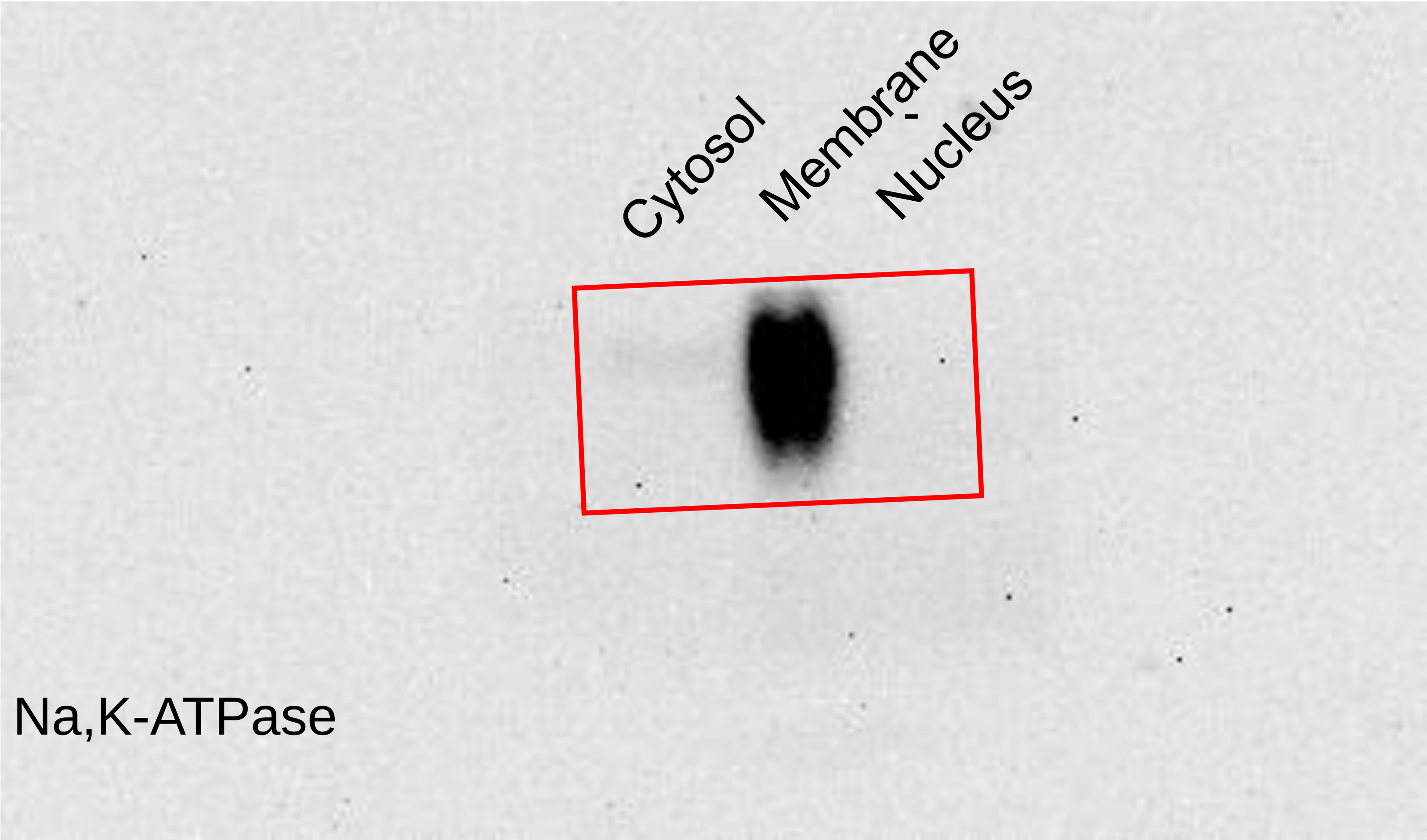

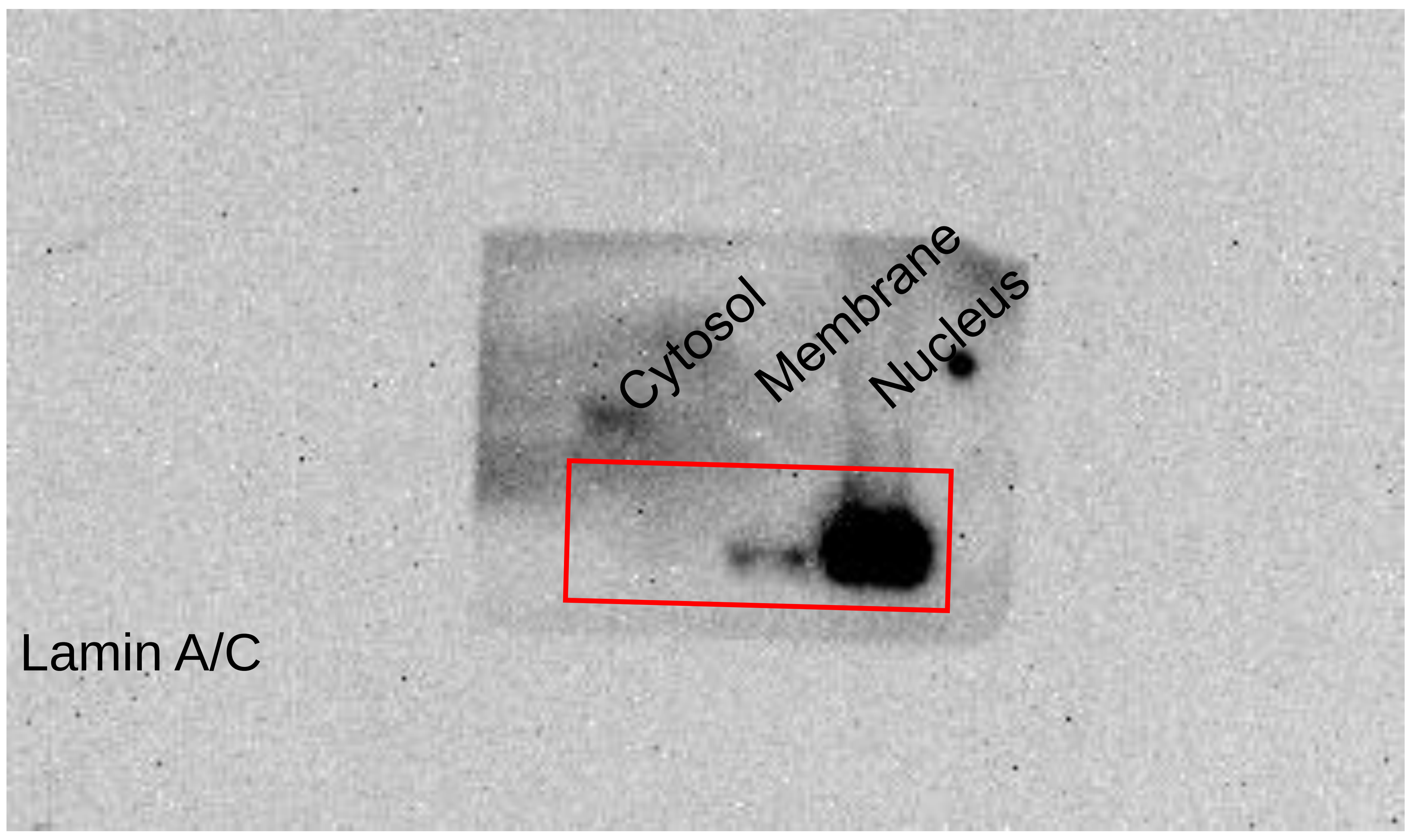

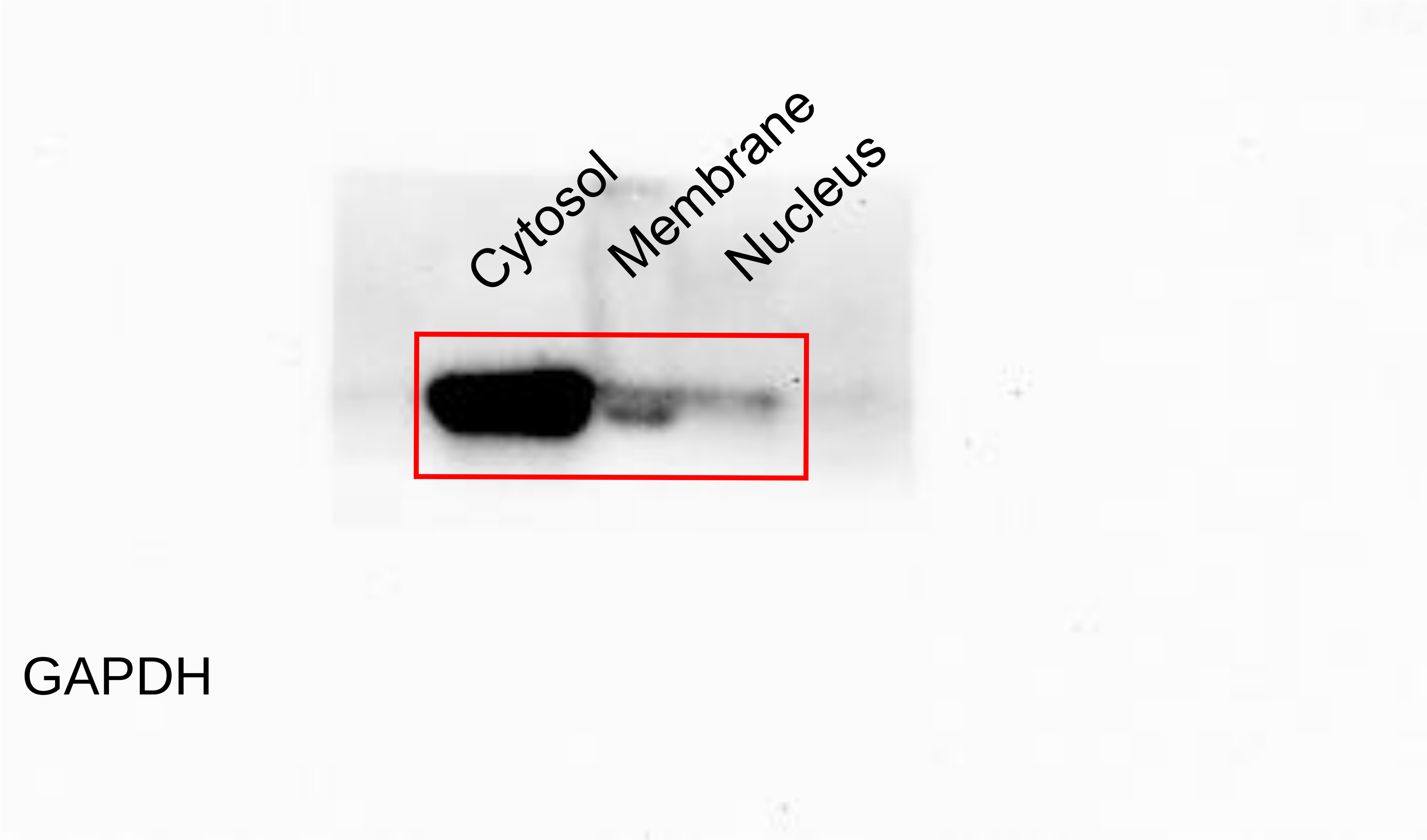

Full unedited gel for Figure 7E

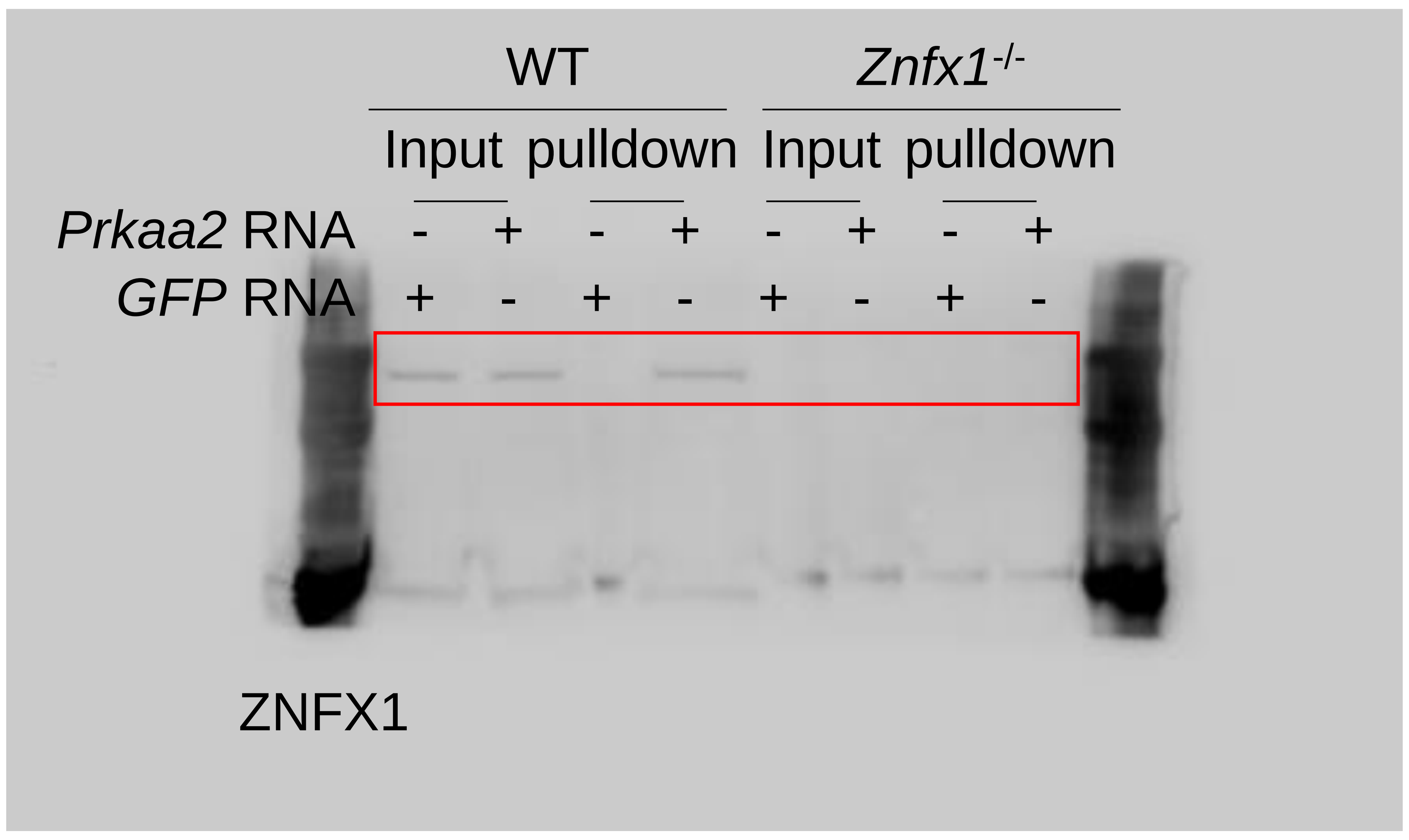

Full unedited gel for Supplementary Figure 2C

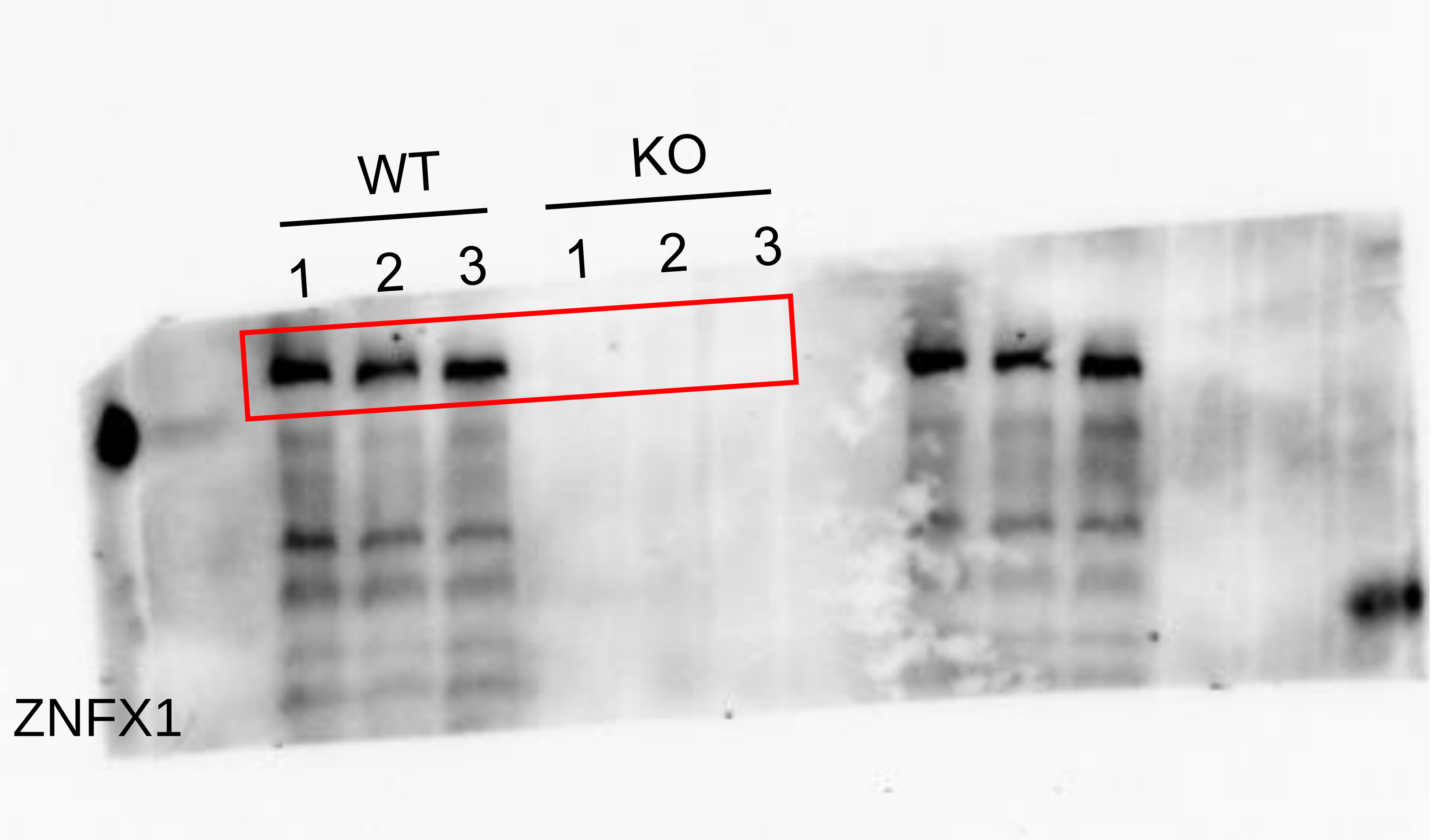

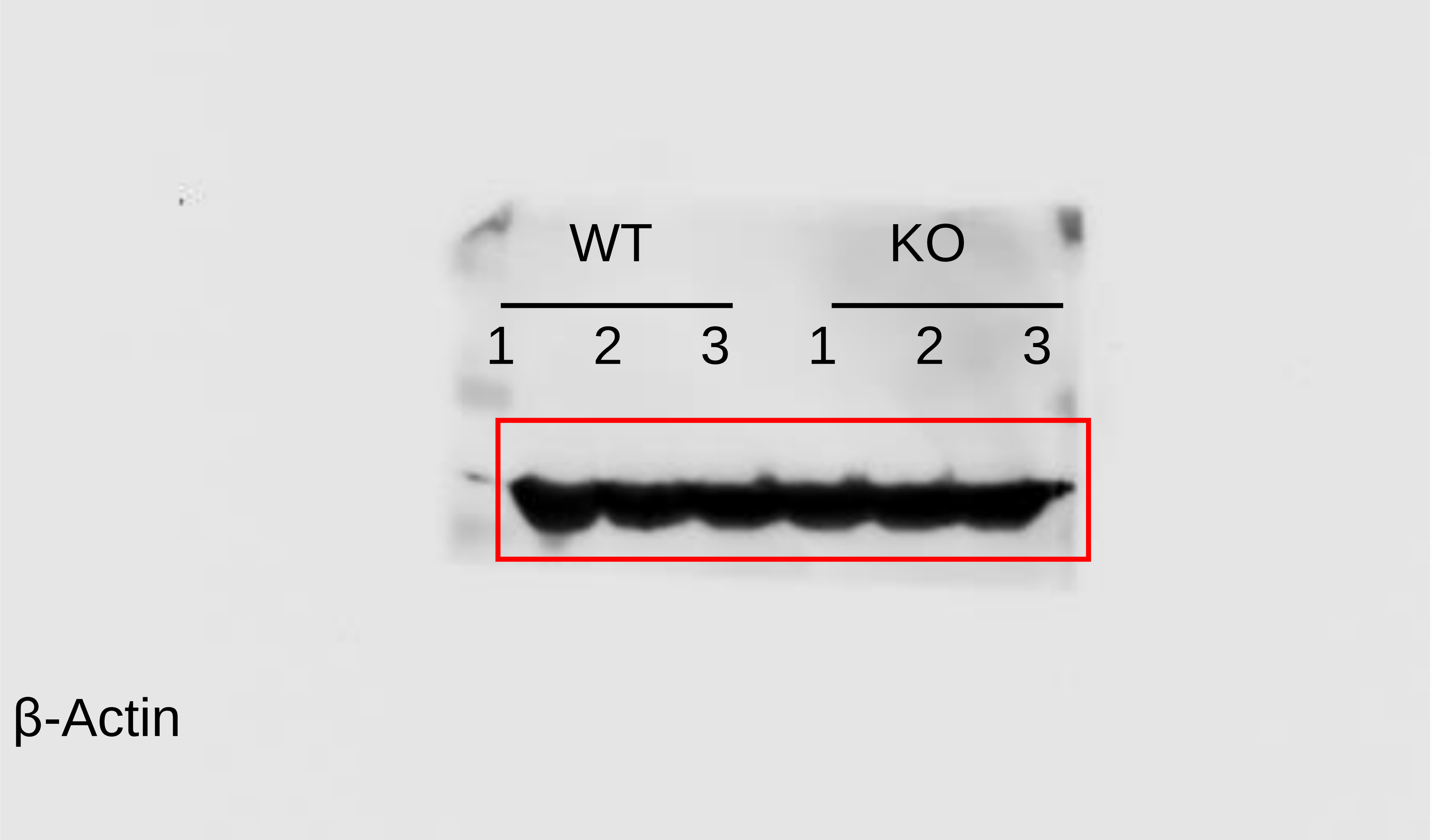

Full unedited gel for Supplementary Figure 4B

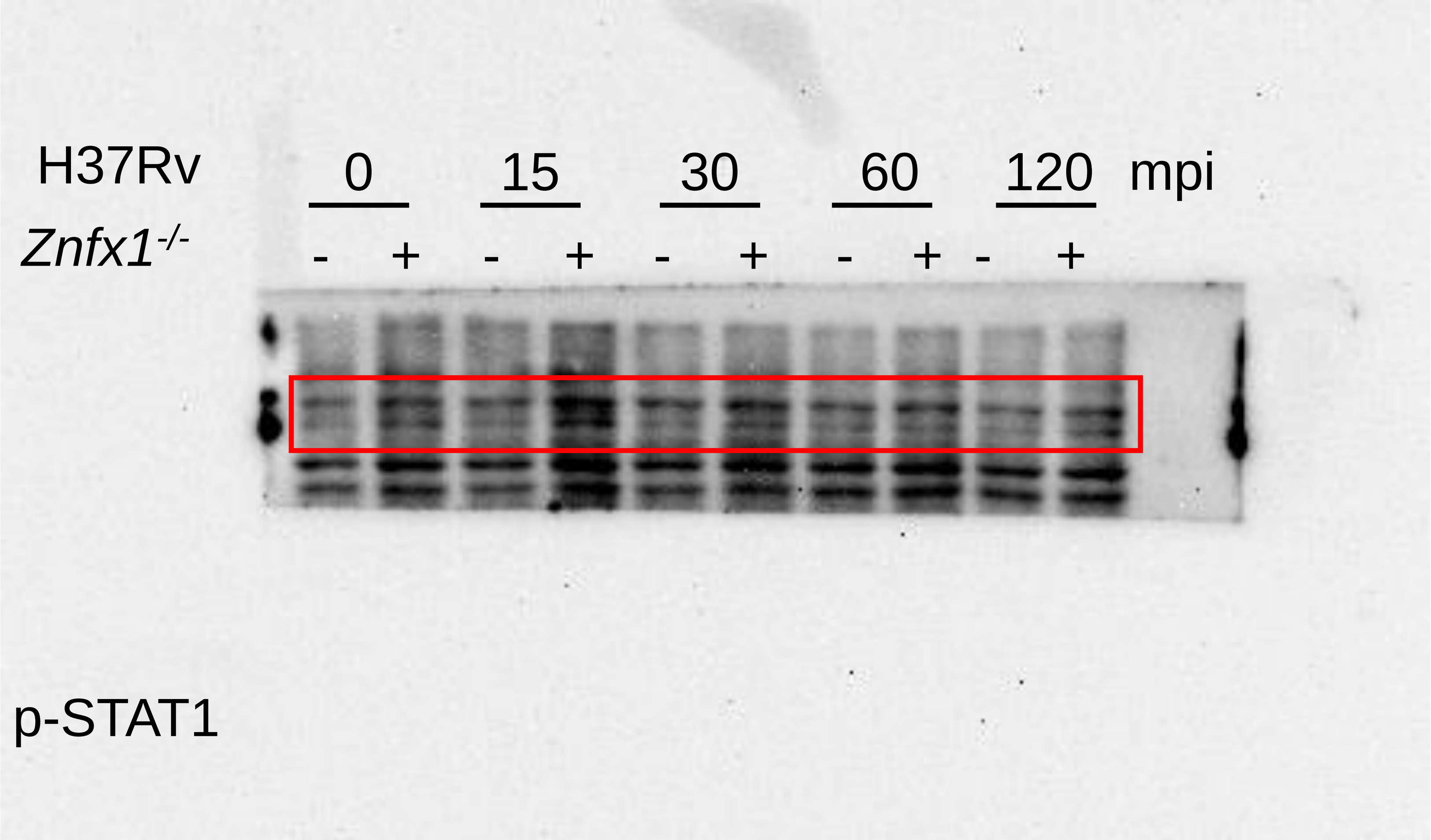

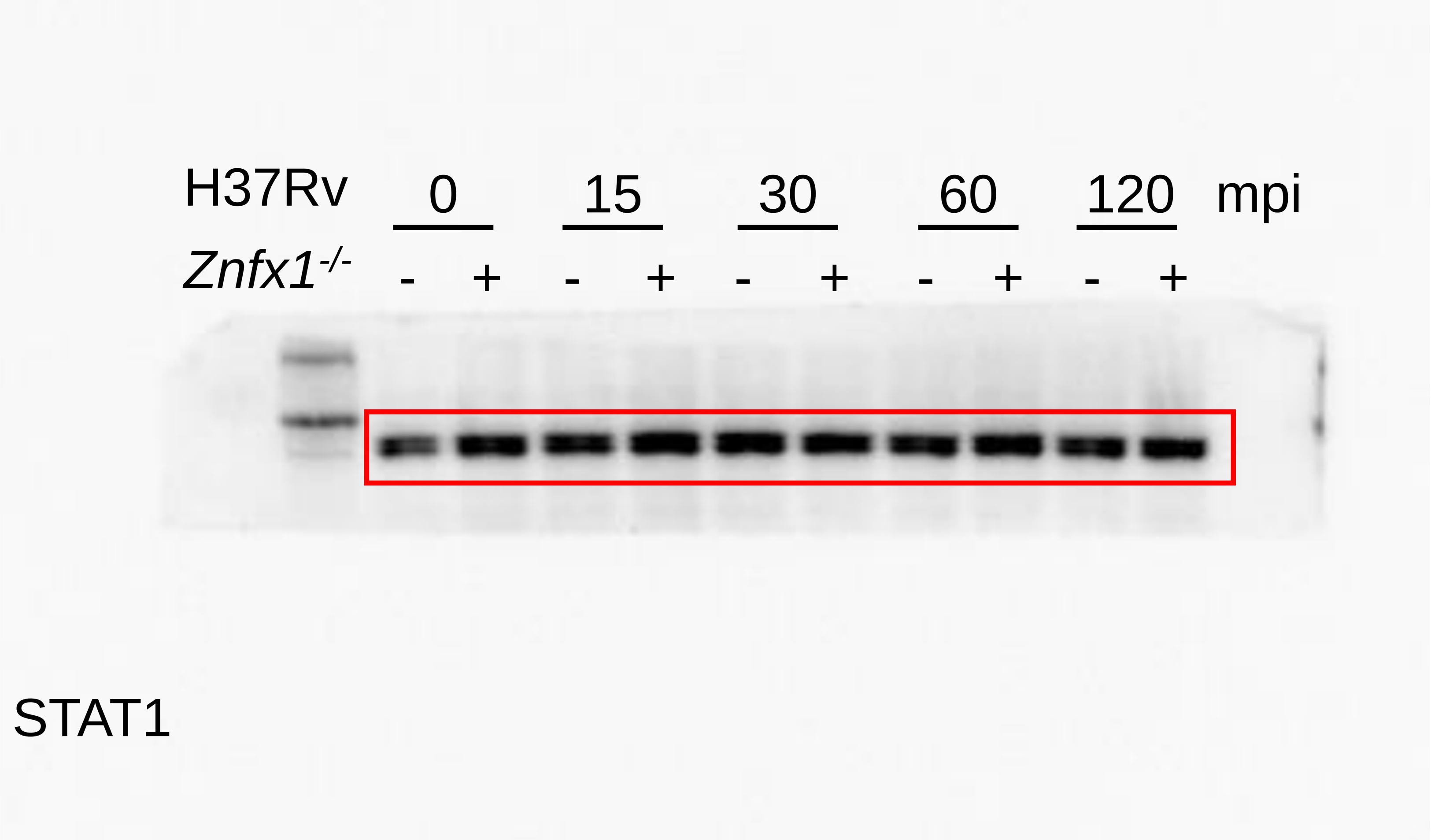

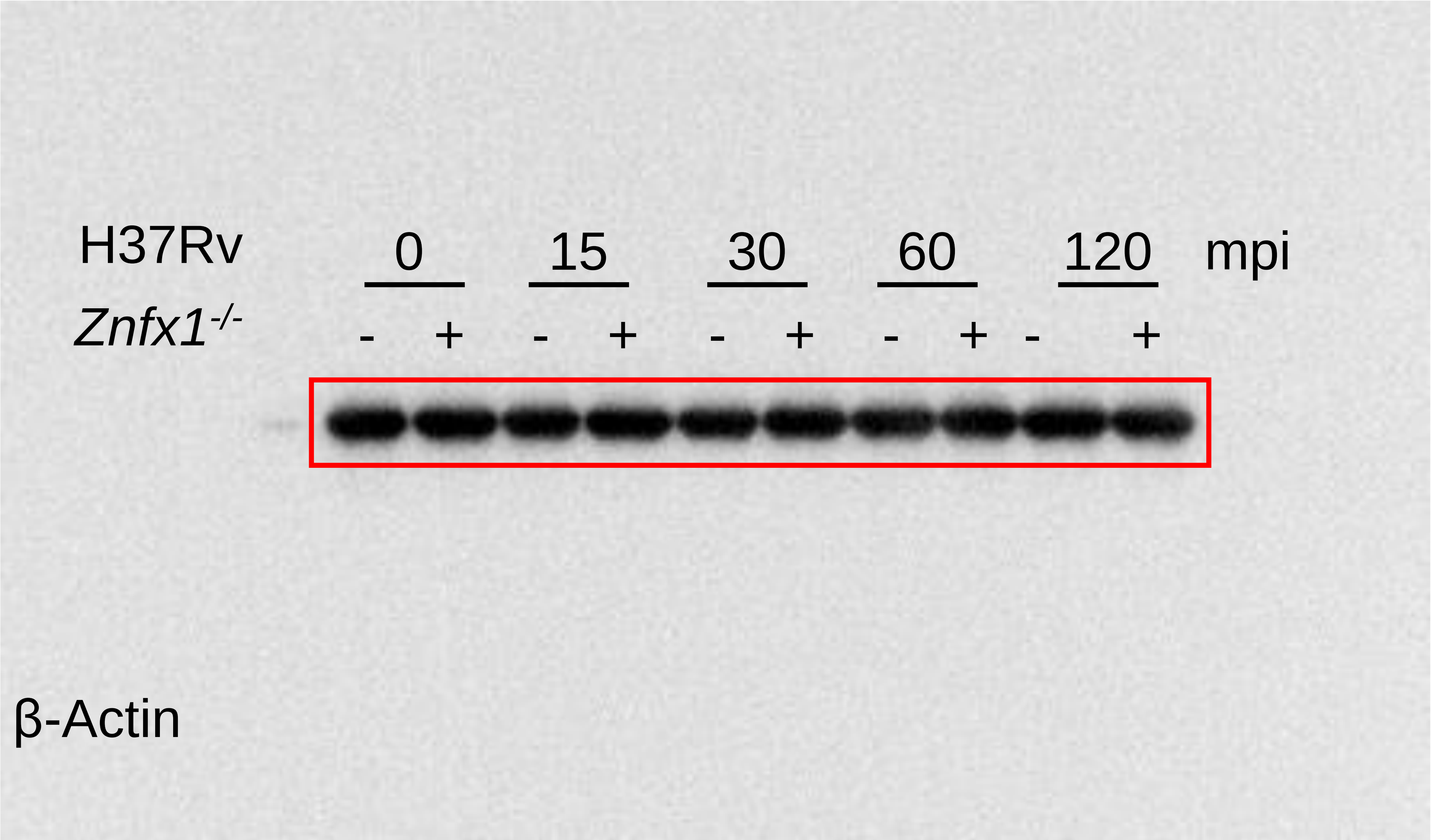

Full unedited gel for Supplementary Figure 5B

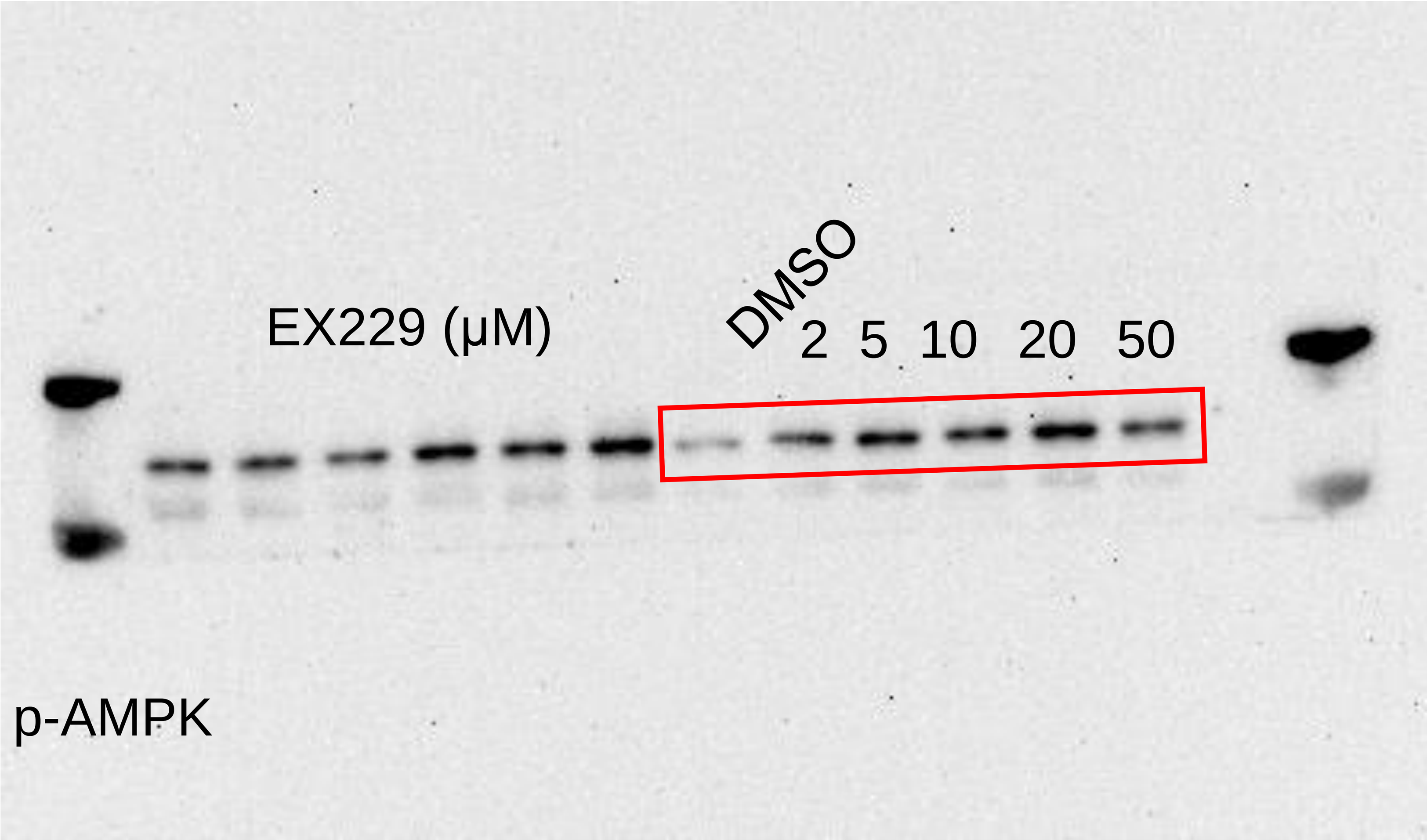

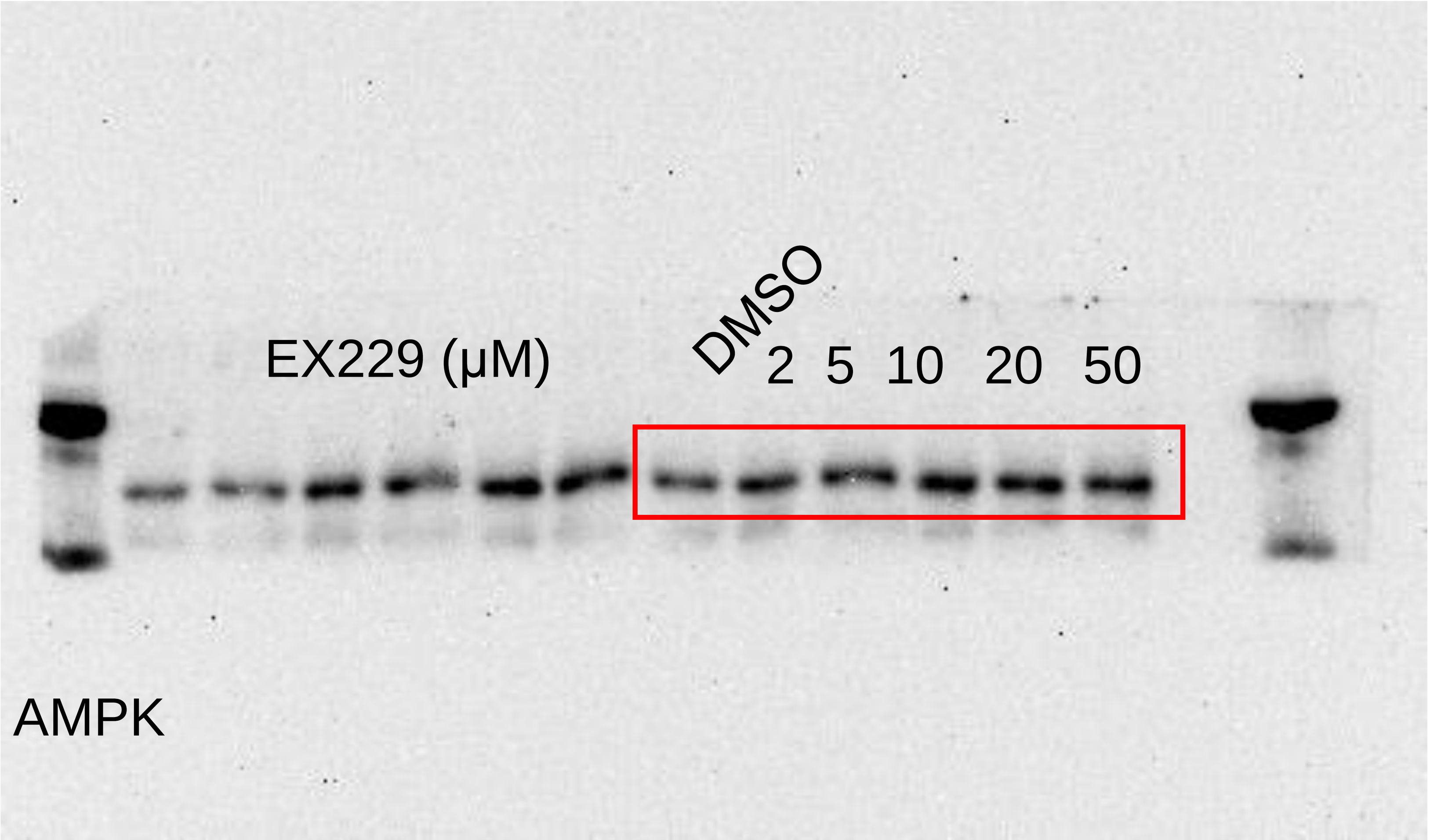

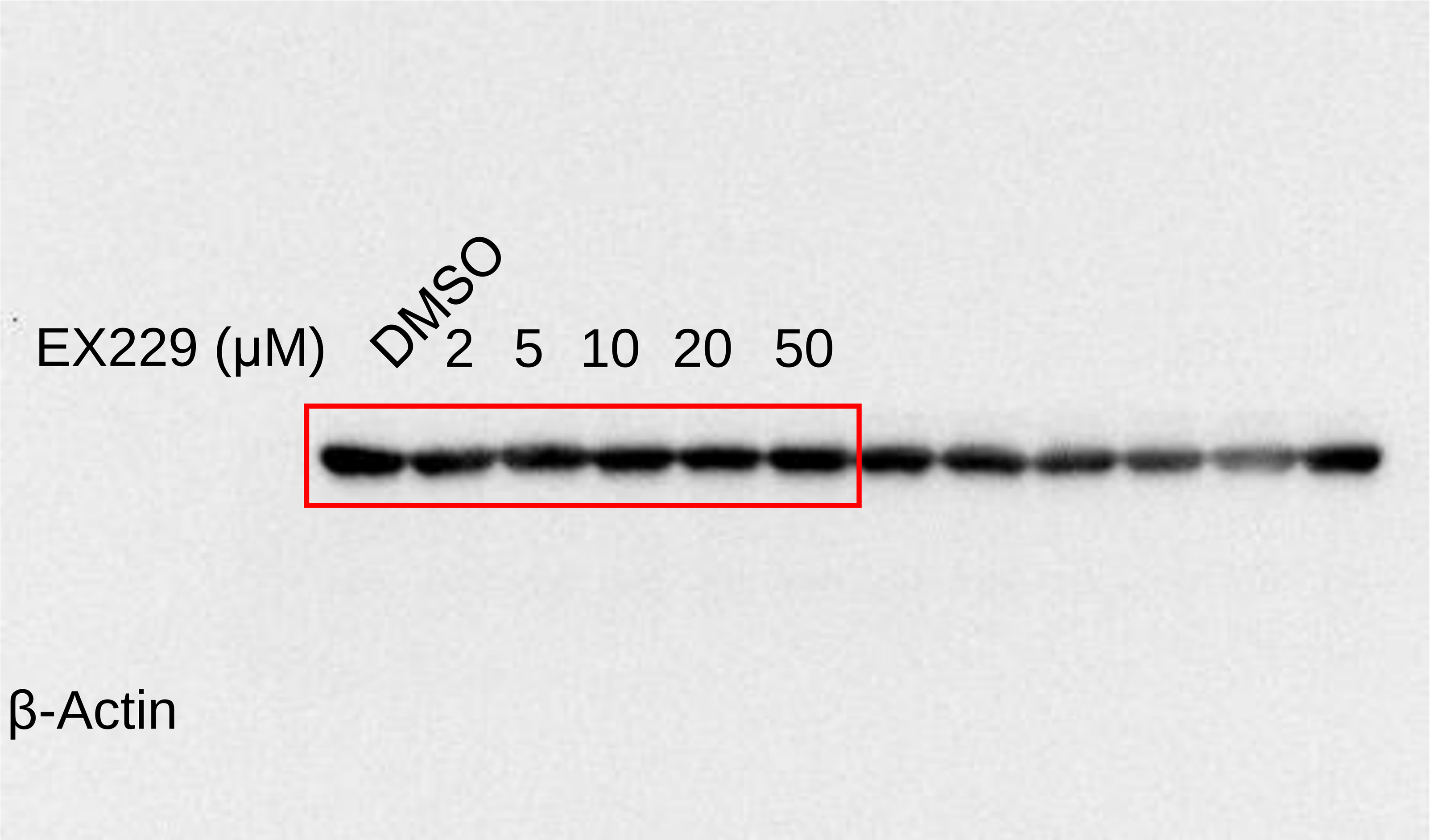

Full unedited gel for Supplementary Figure 5C

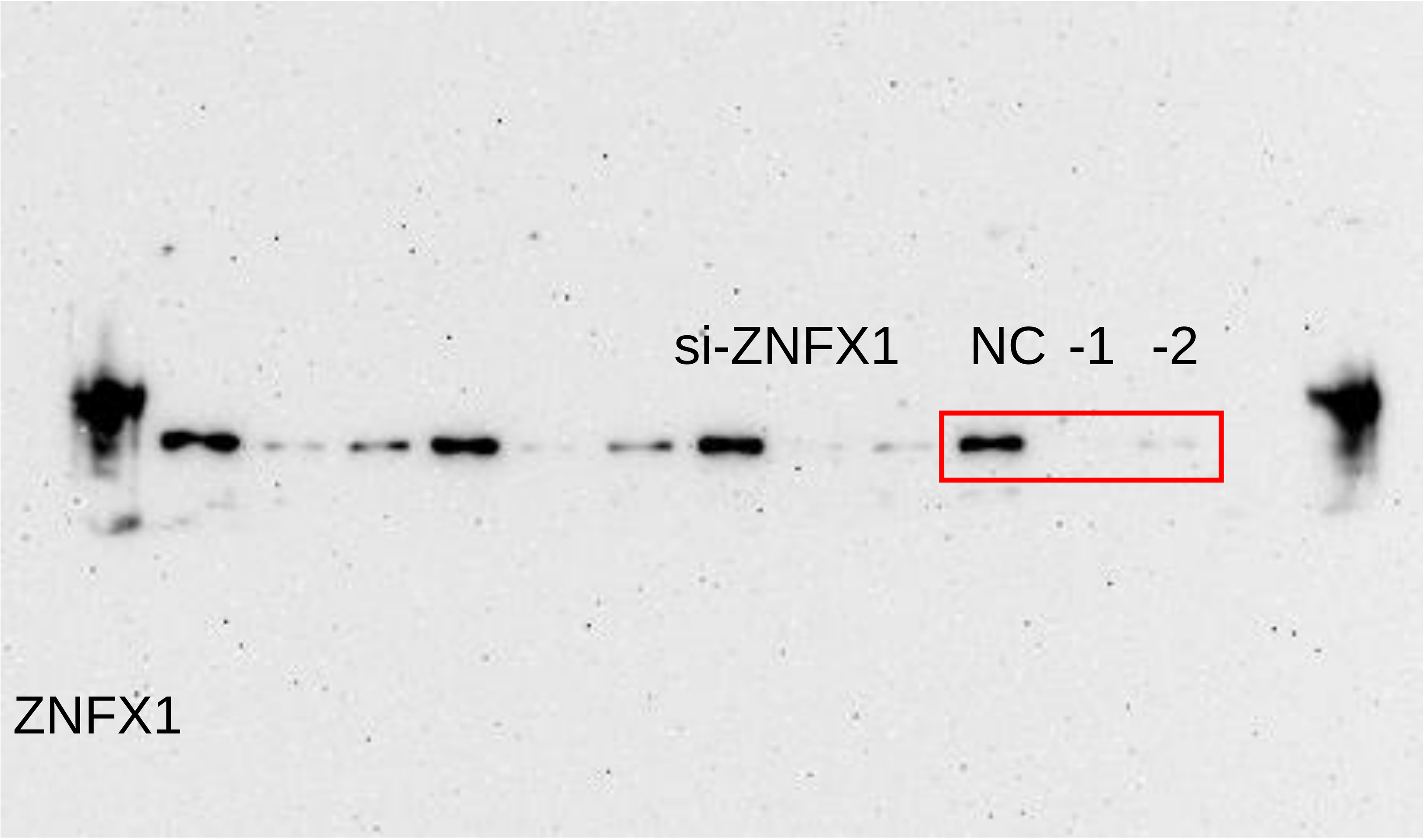

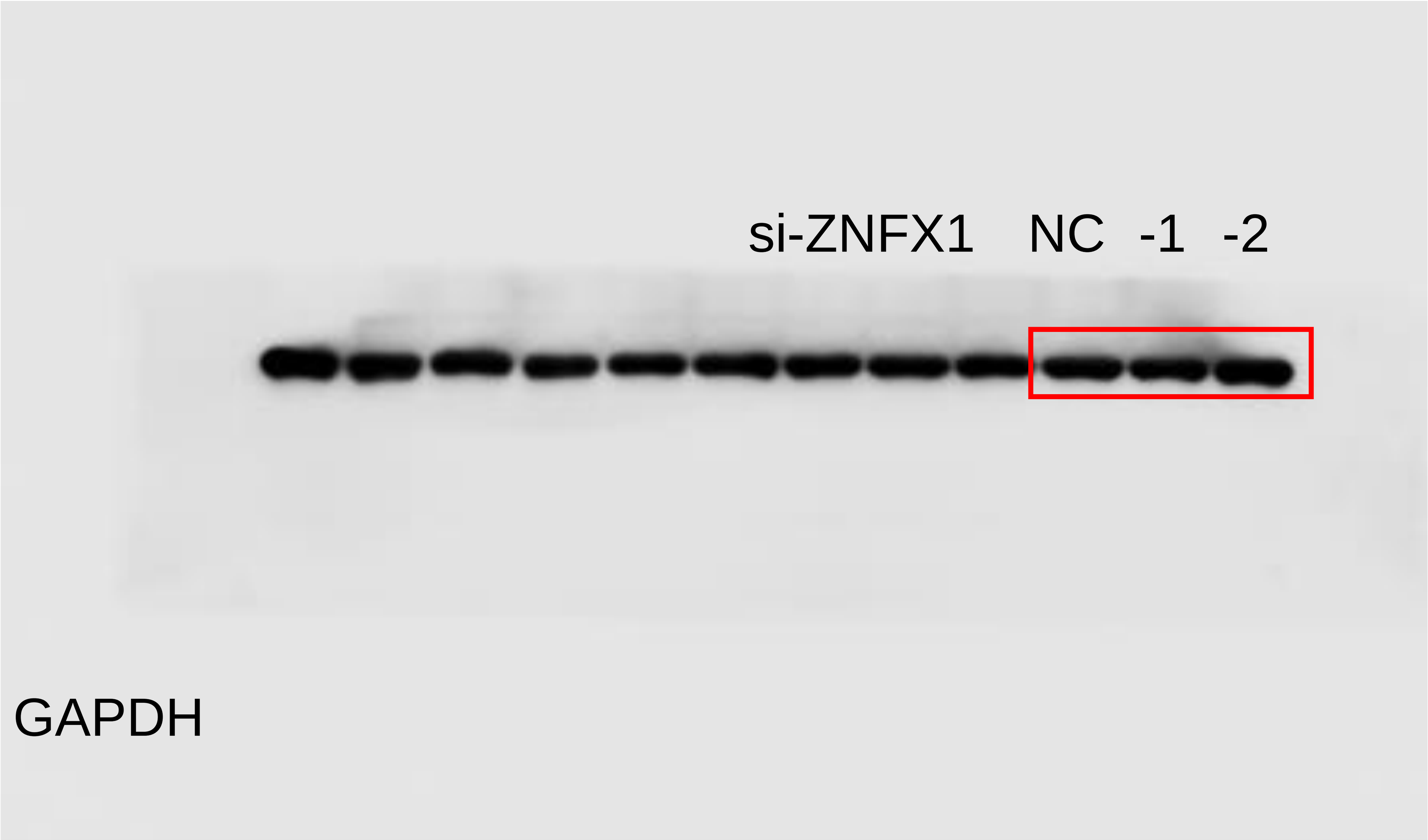

Full unedited gel for Supplementary Figure 5E

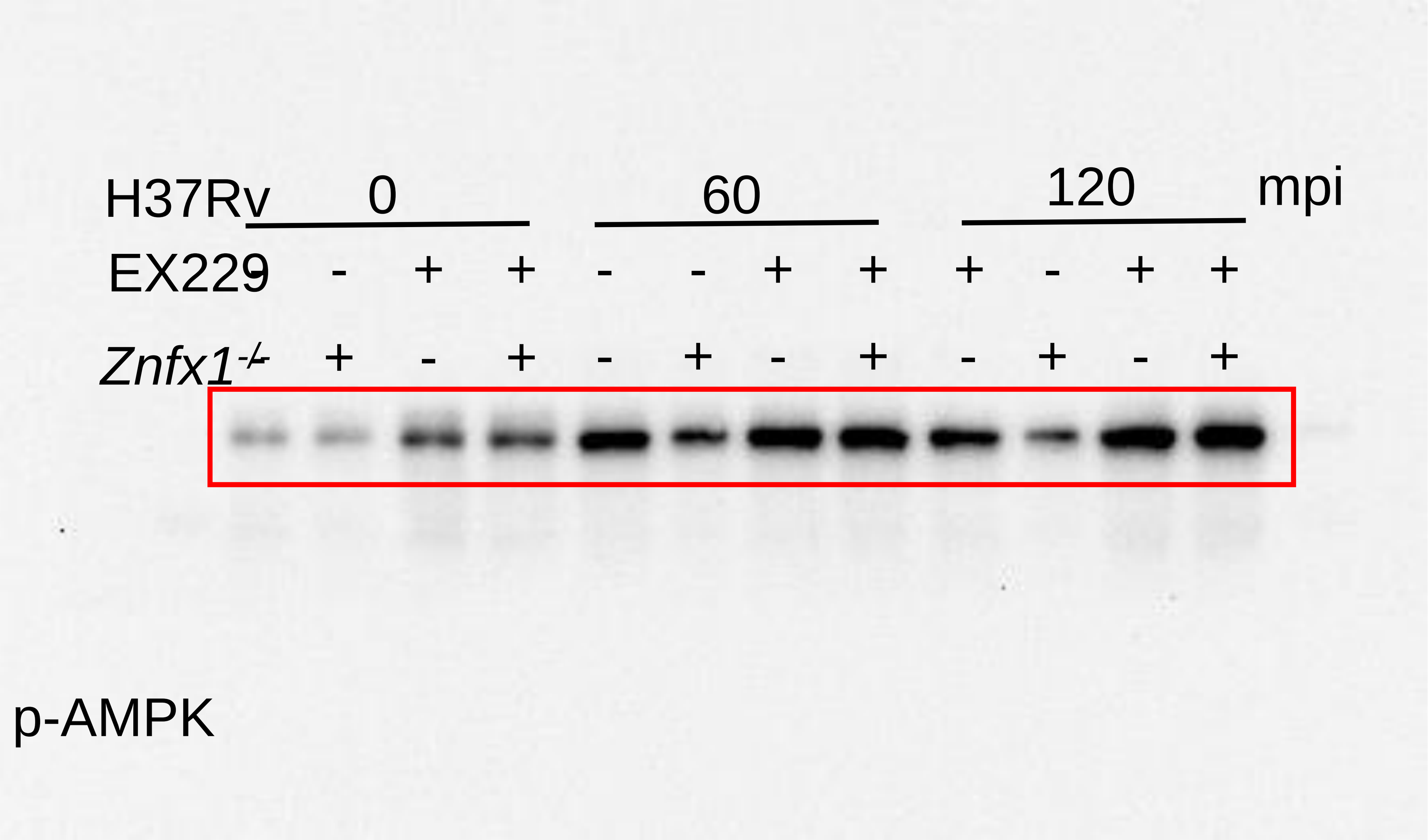

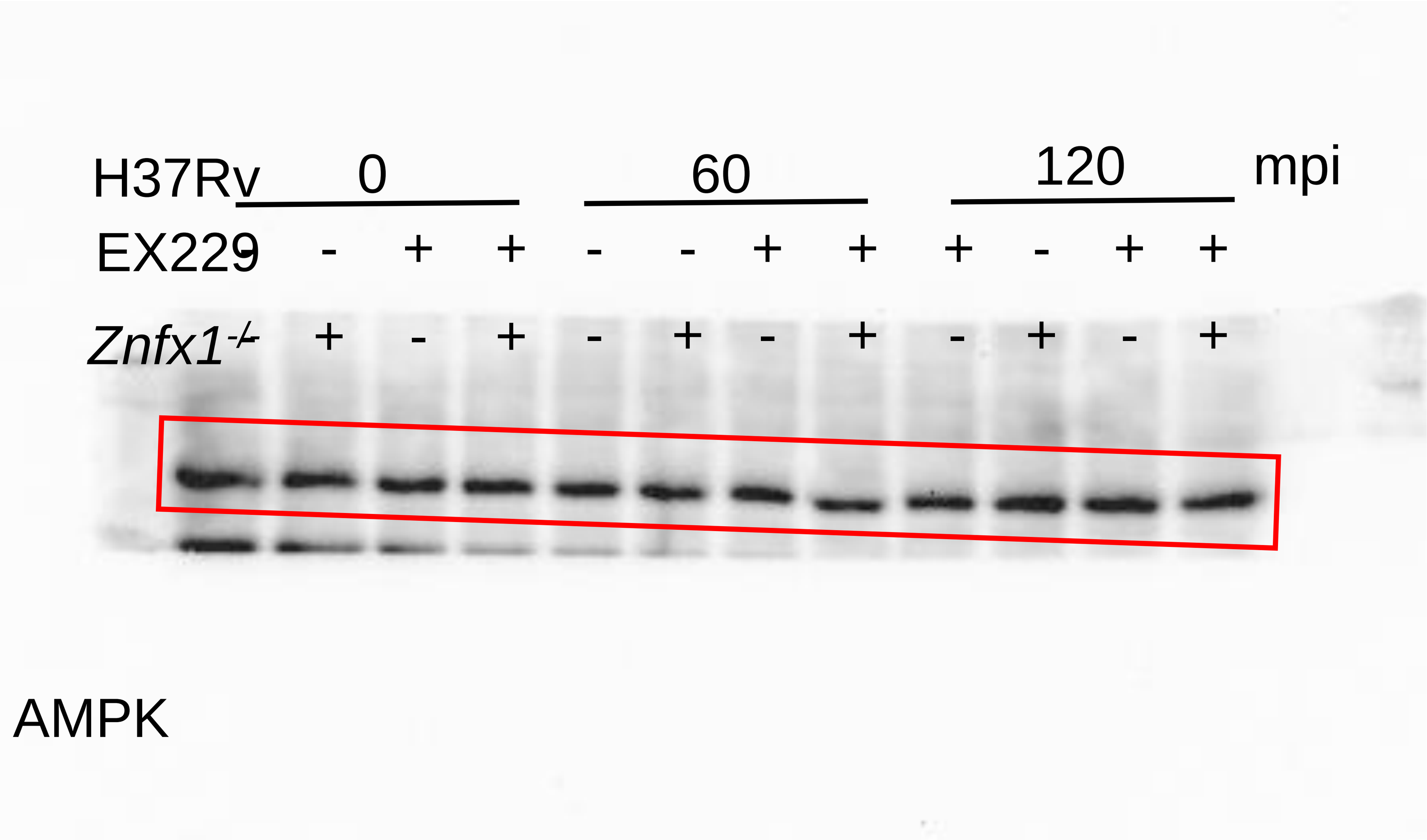

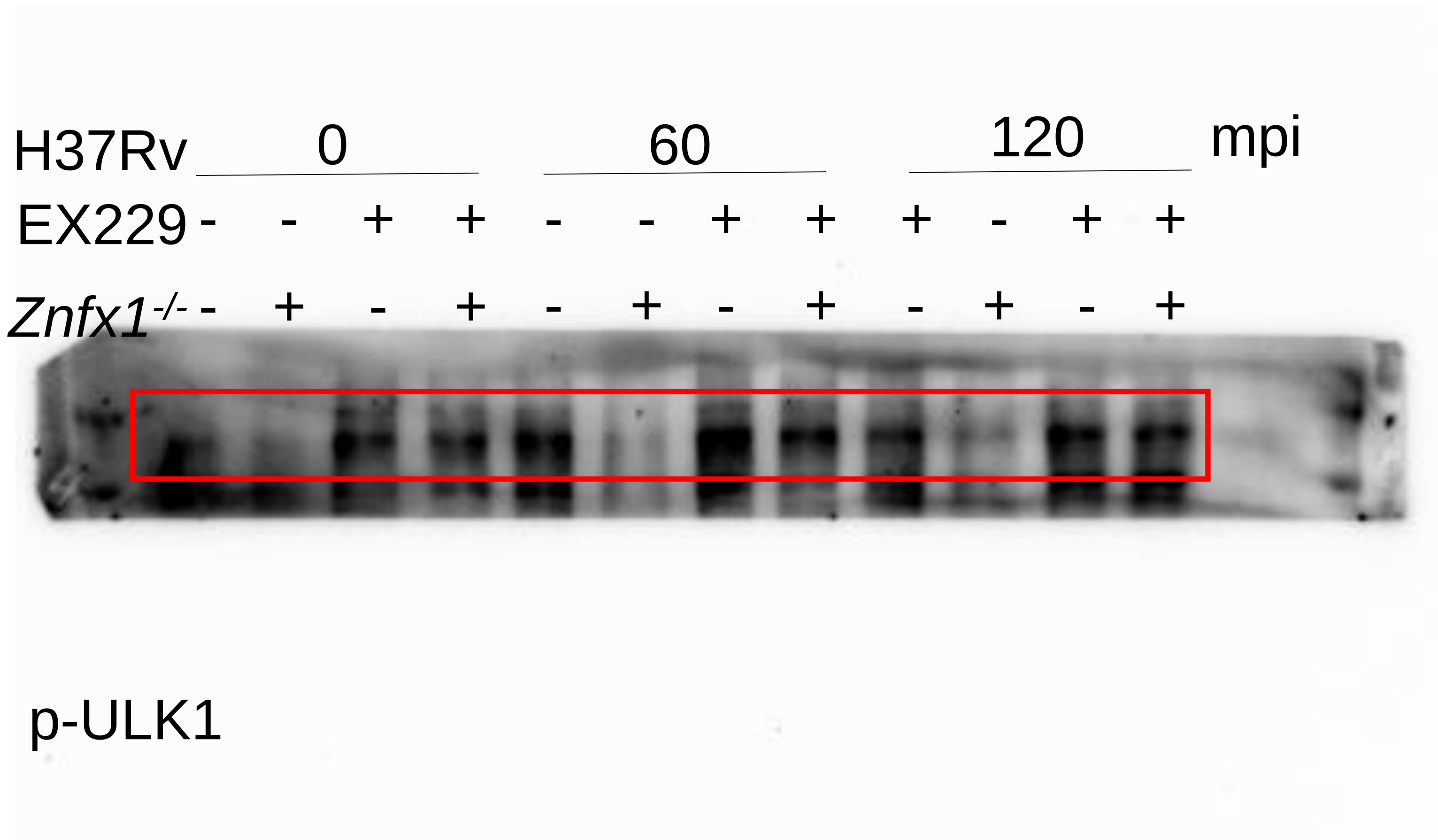

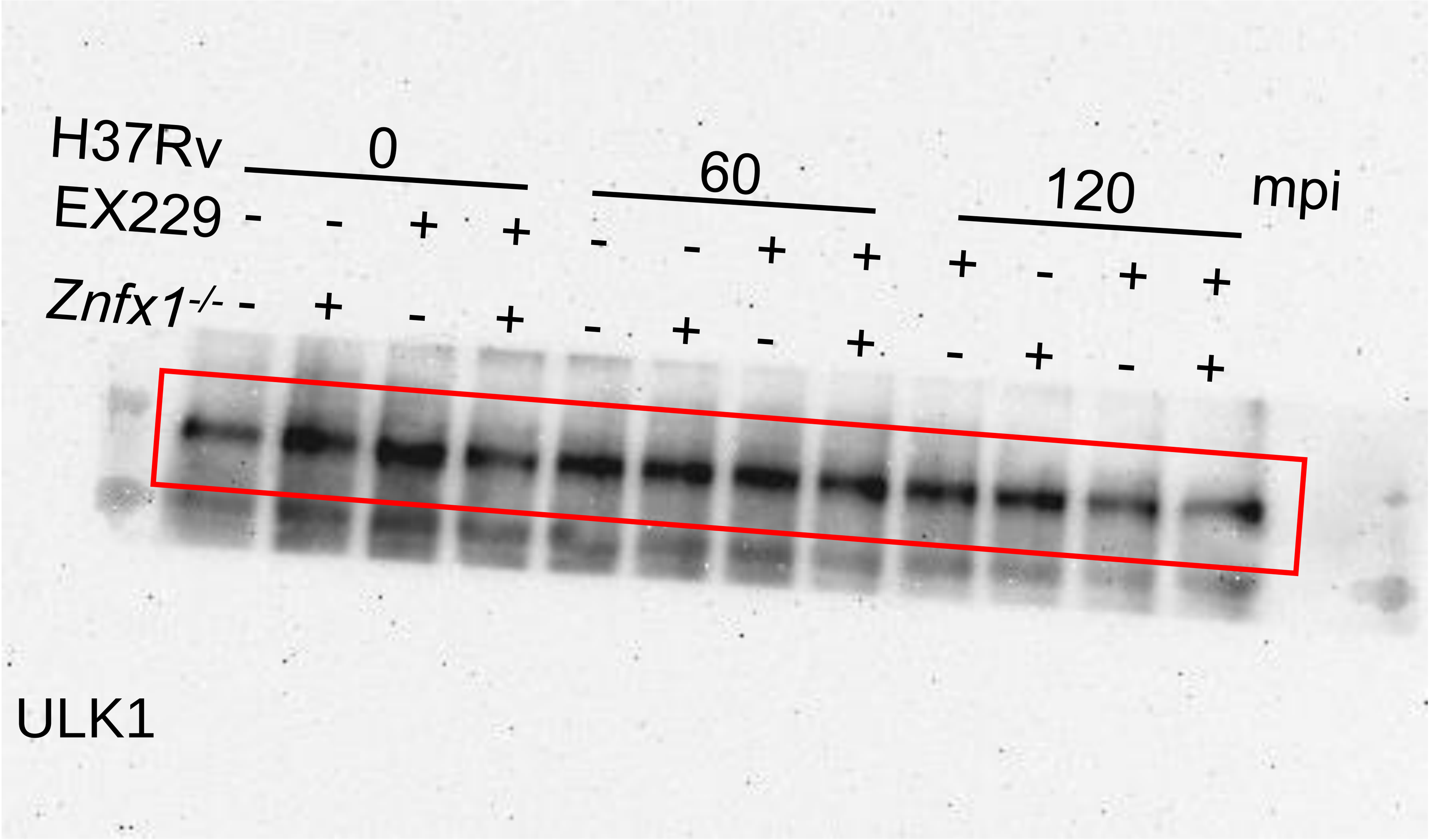

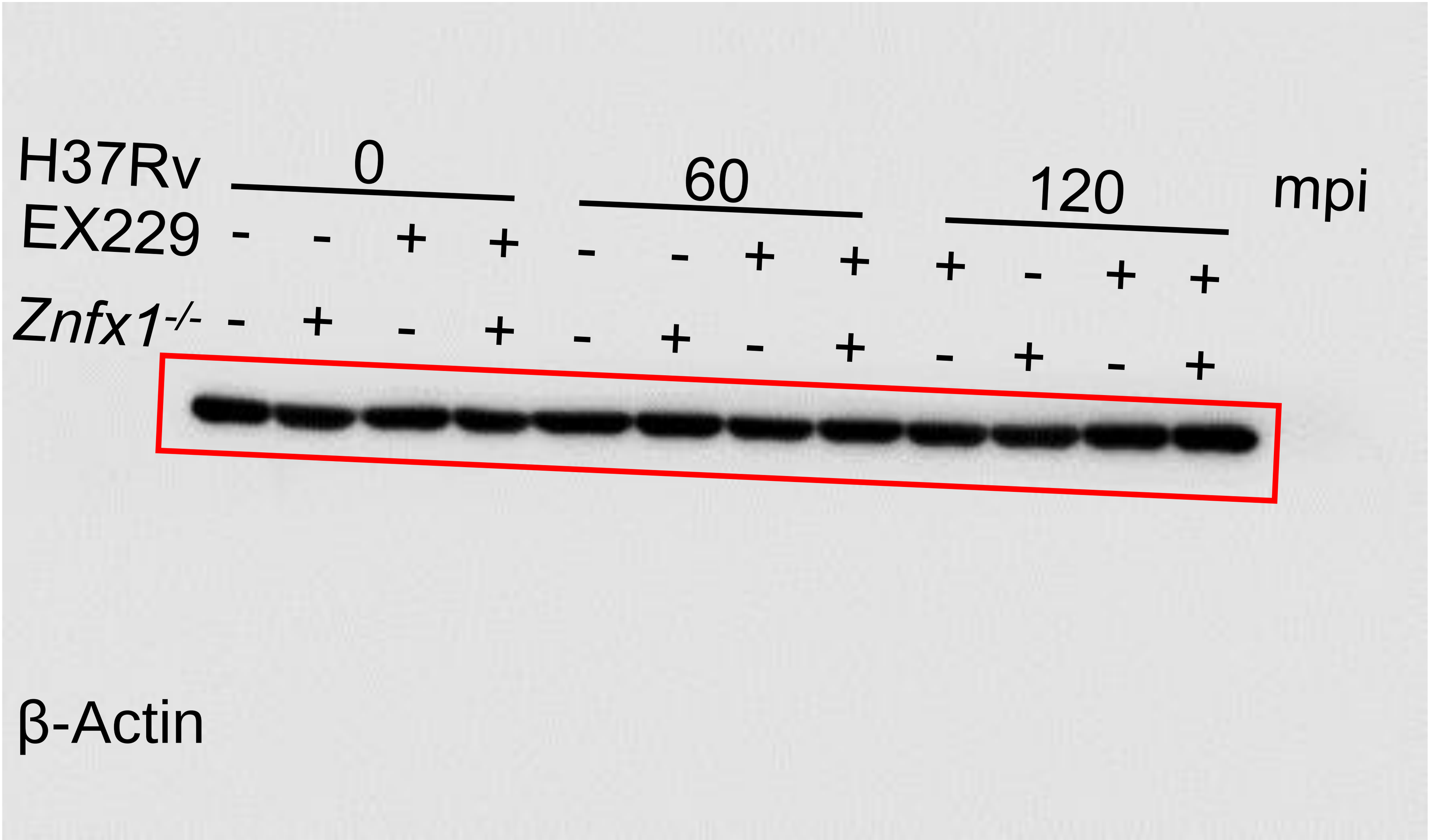

Full unedited gel for Supplementary Figure 5F

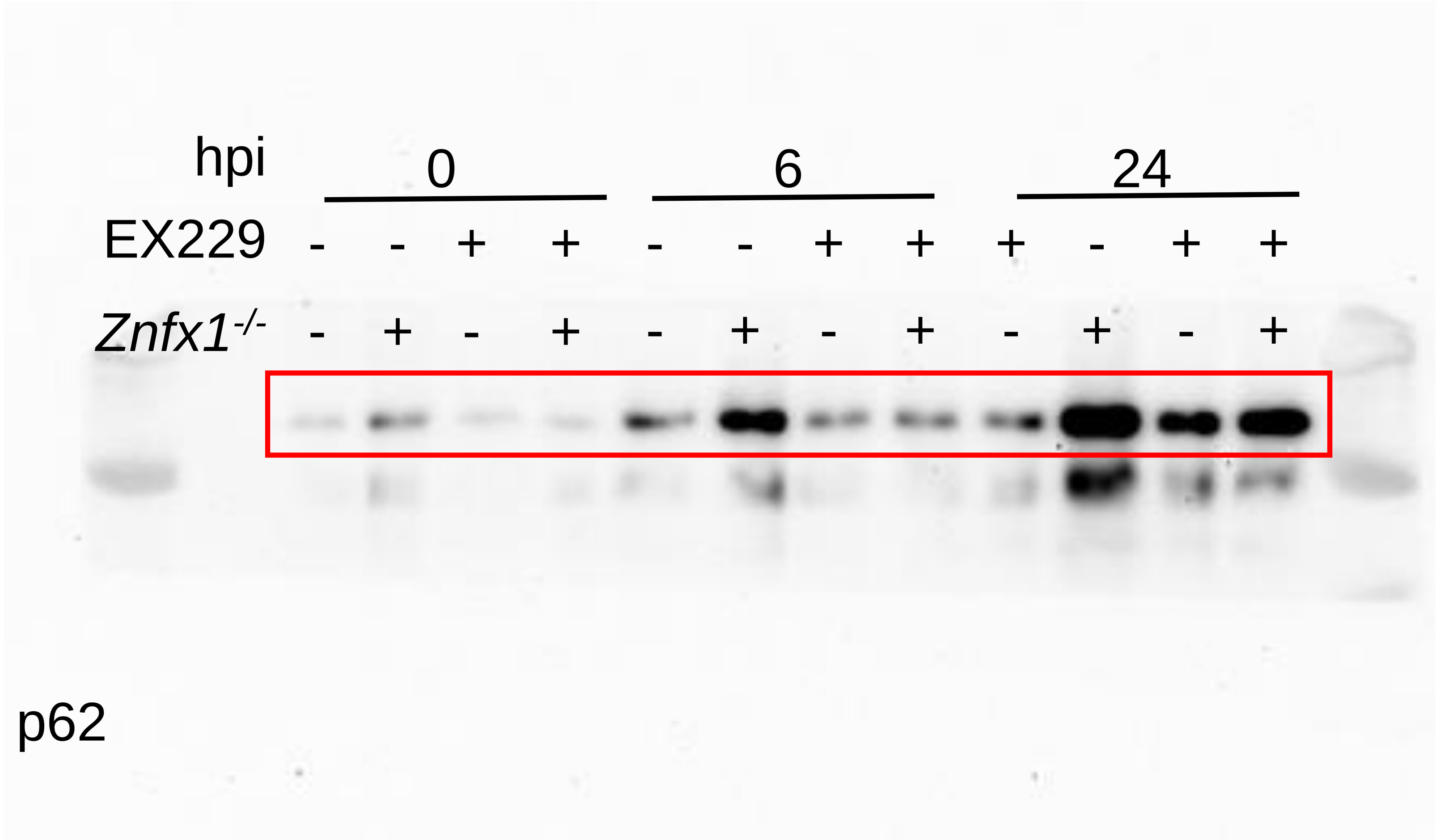

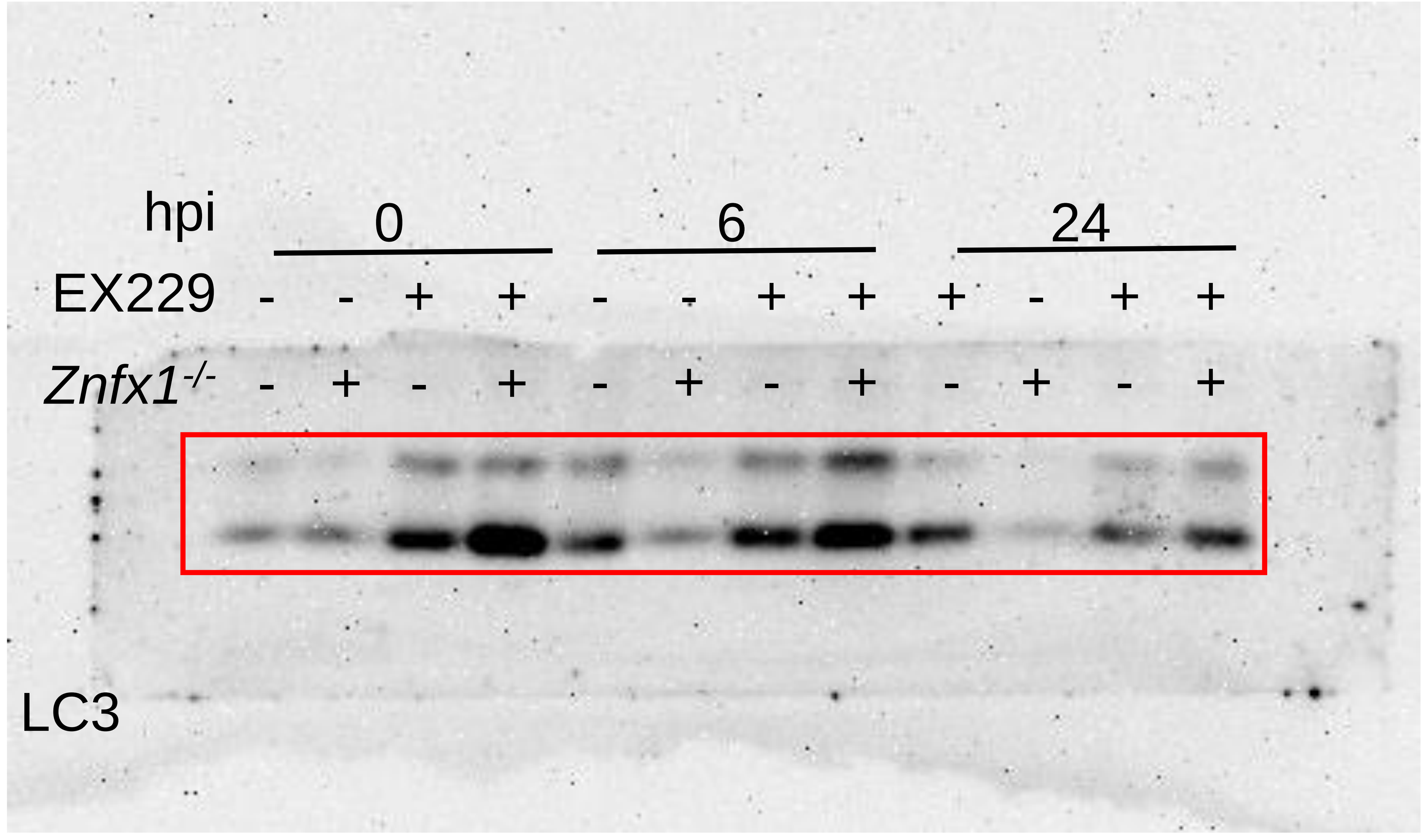

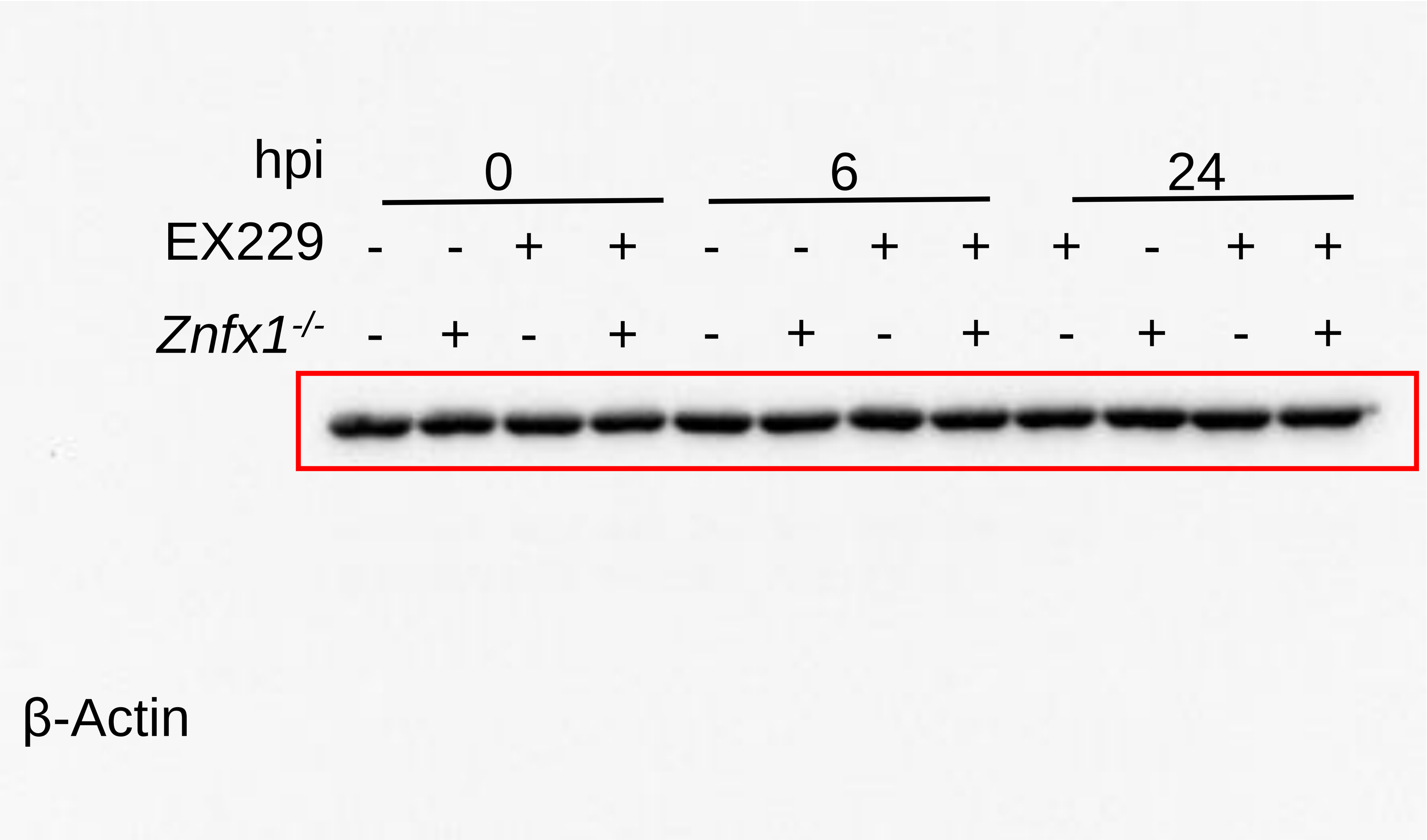

Full unedited gel for Supplementary Figure 5H

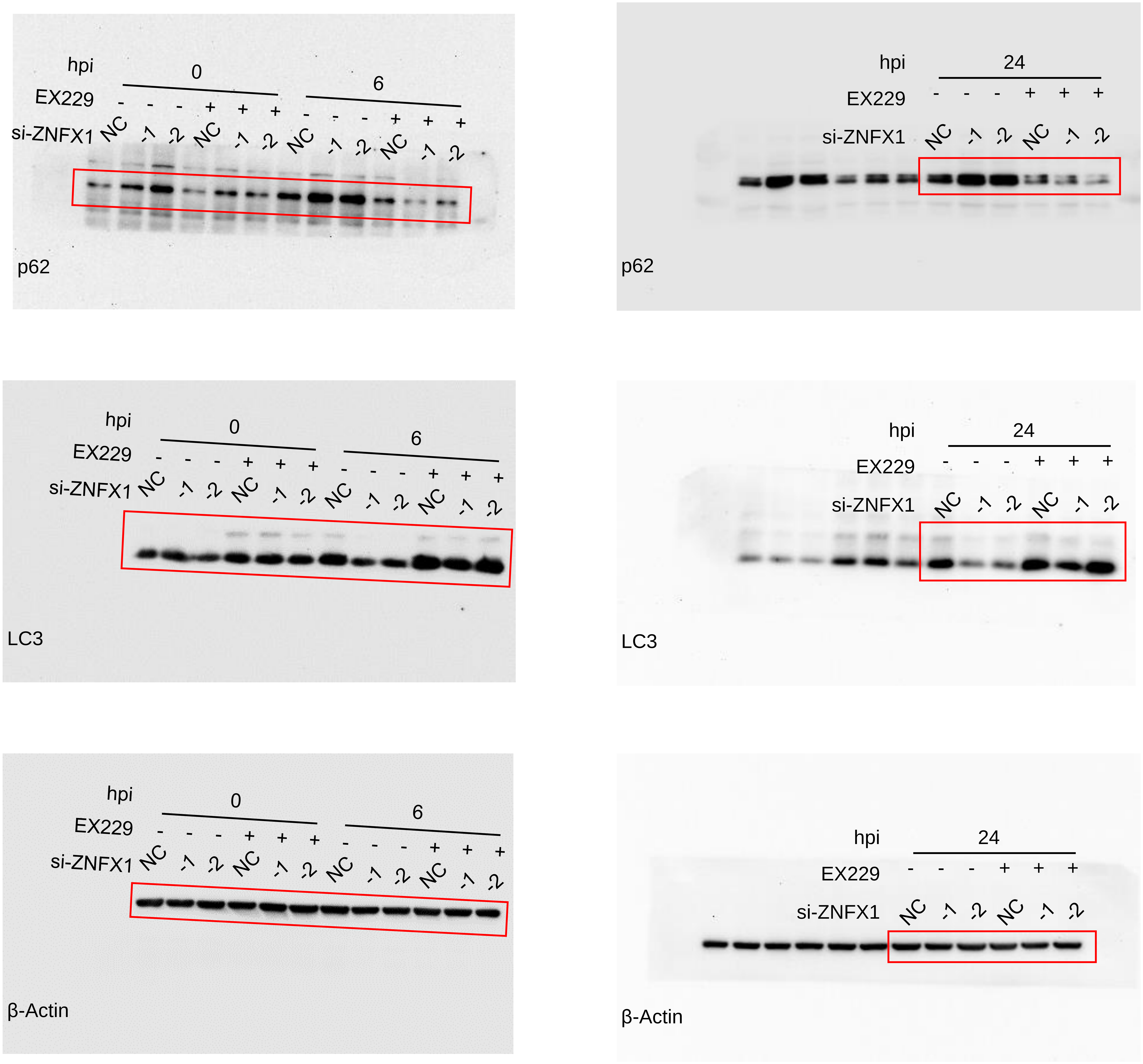

Full unedited gel for Supplementary Figure 6A

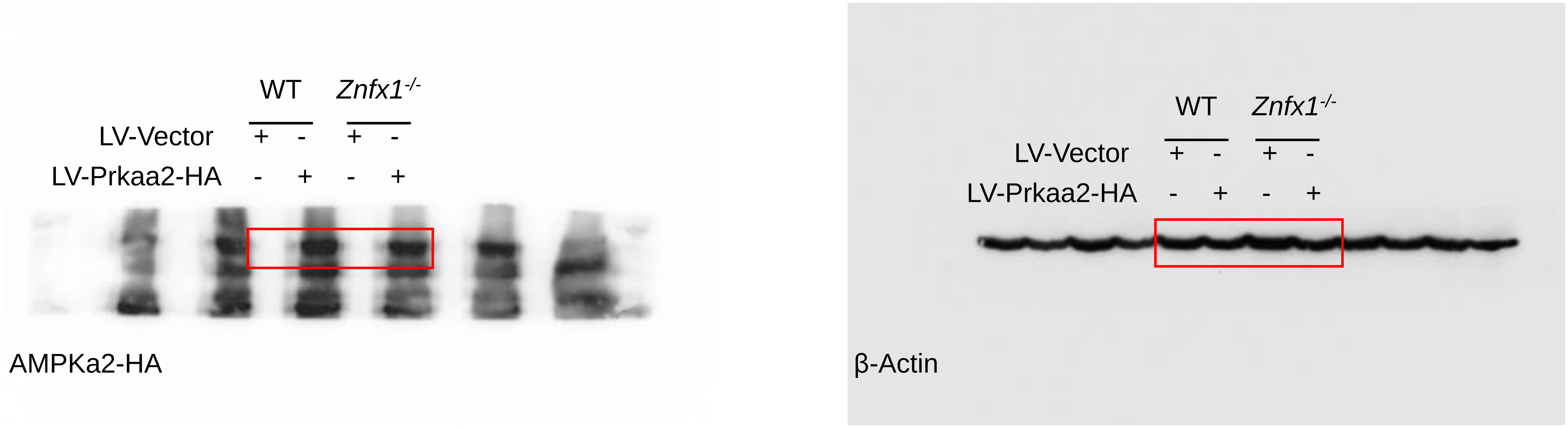

Full unedited gel for Supplementary Figure 7A

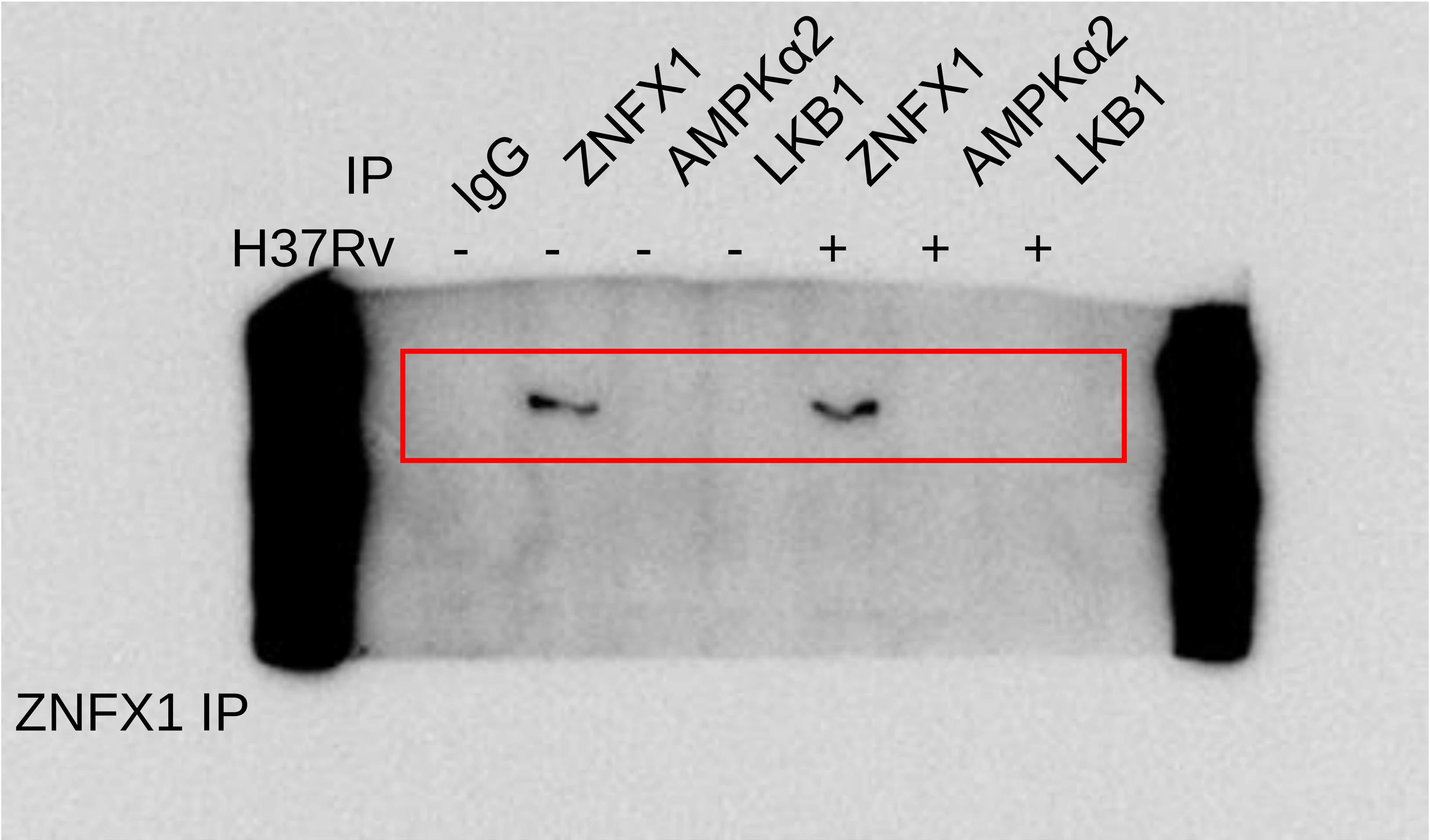

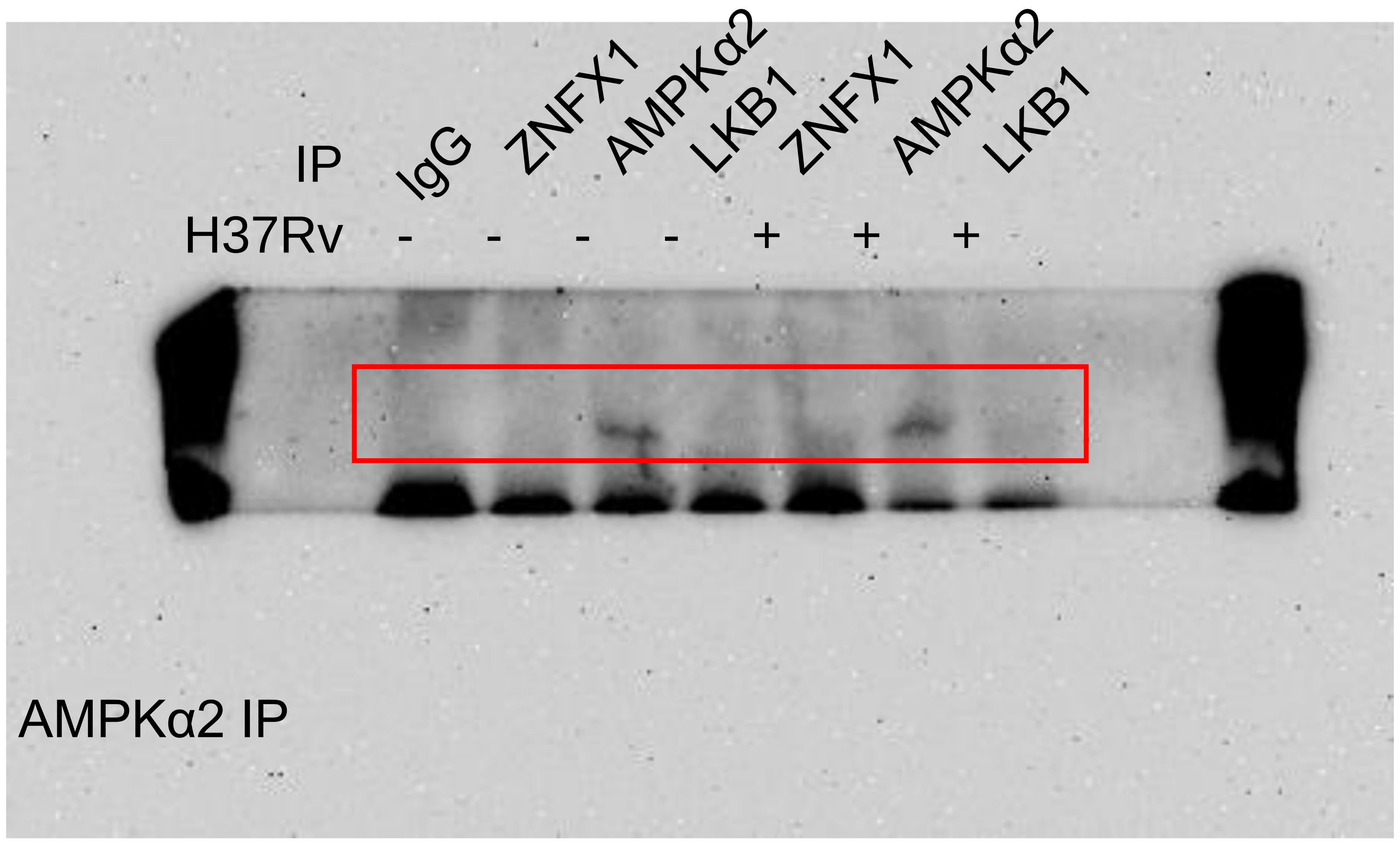

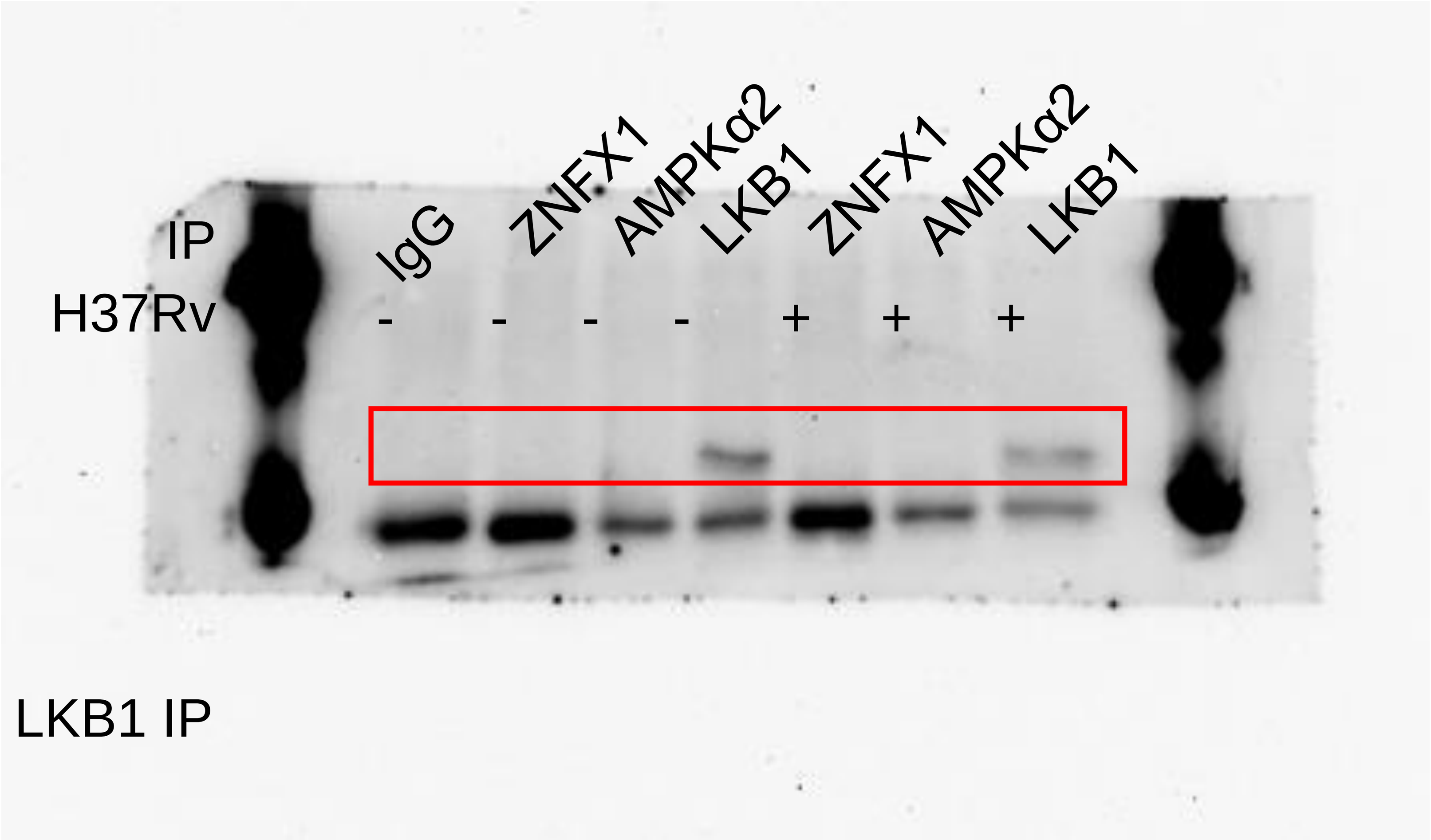

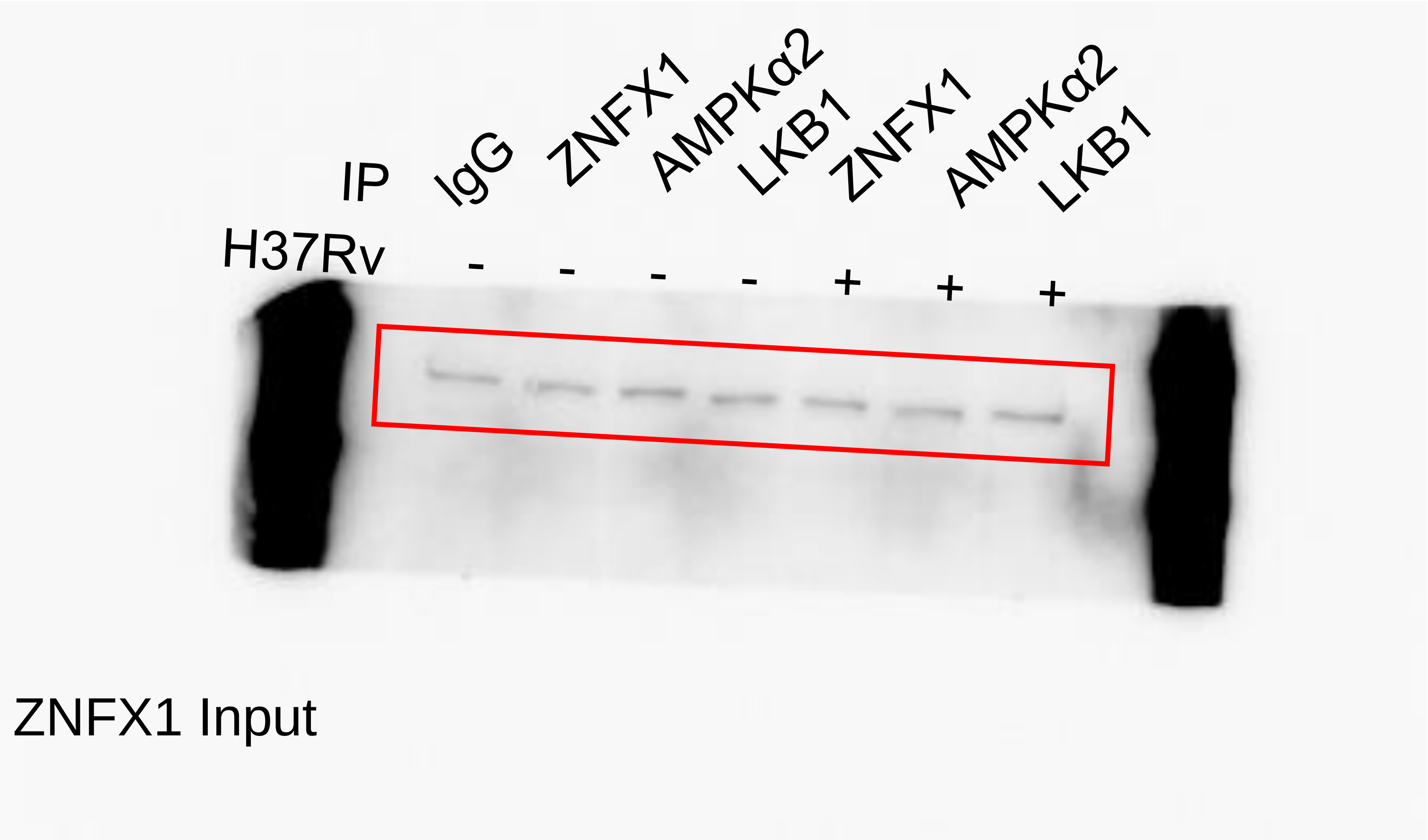

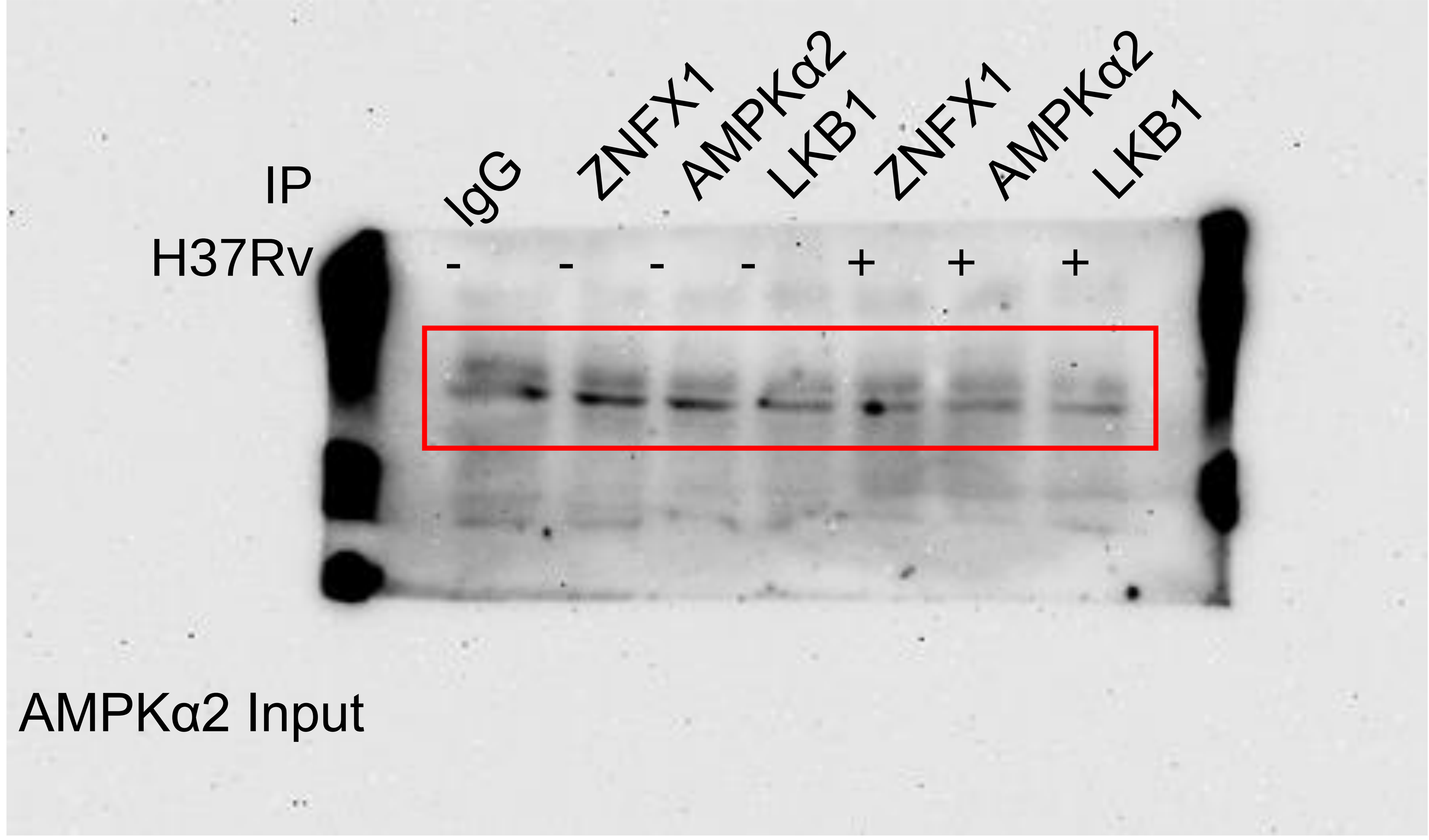

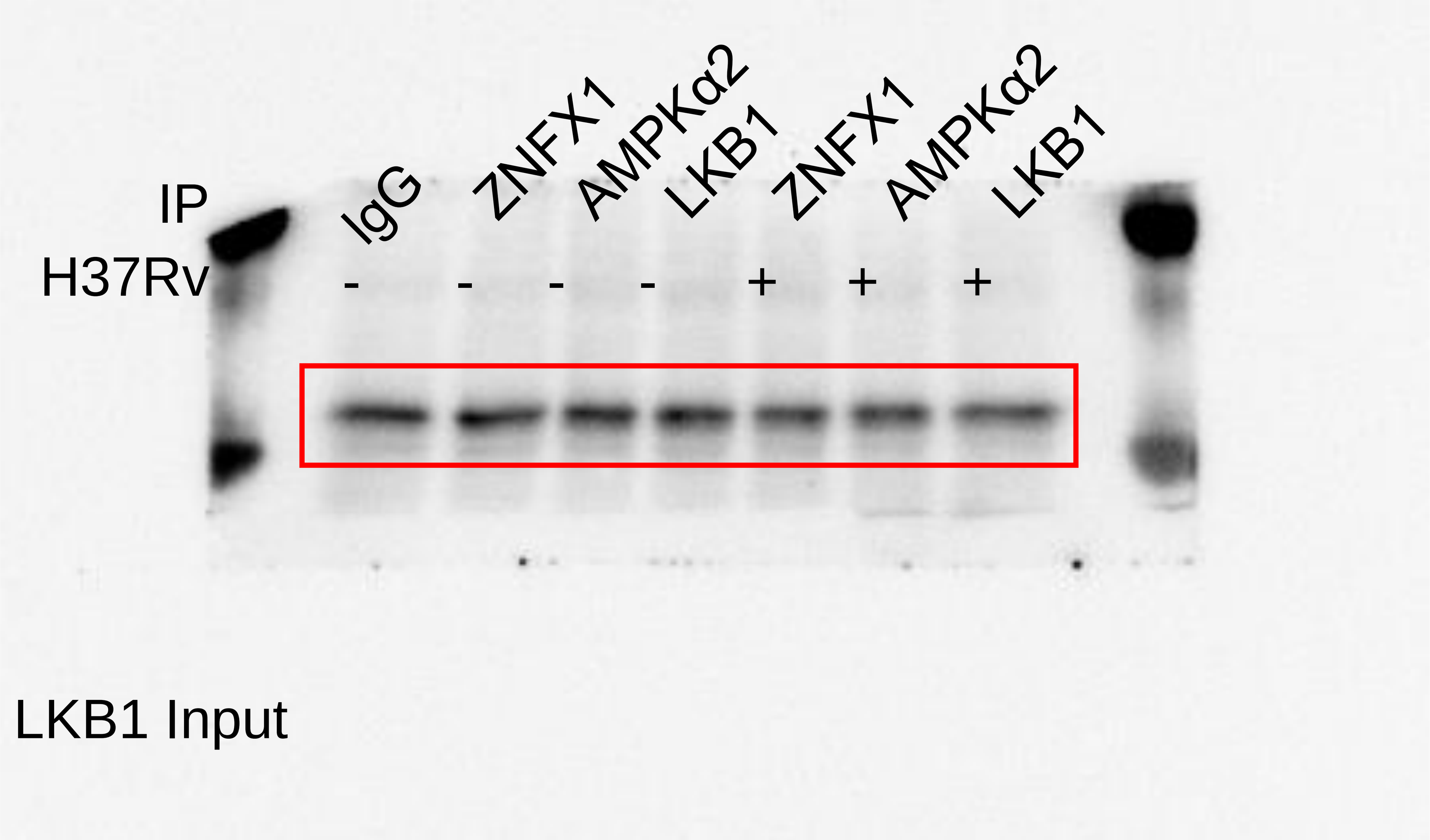

Full unedited gel for Figure 7B

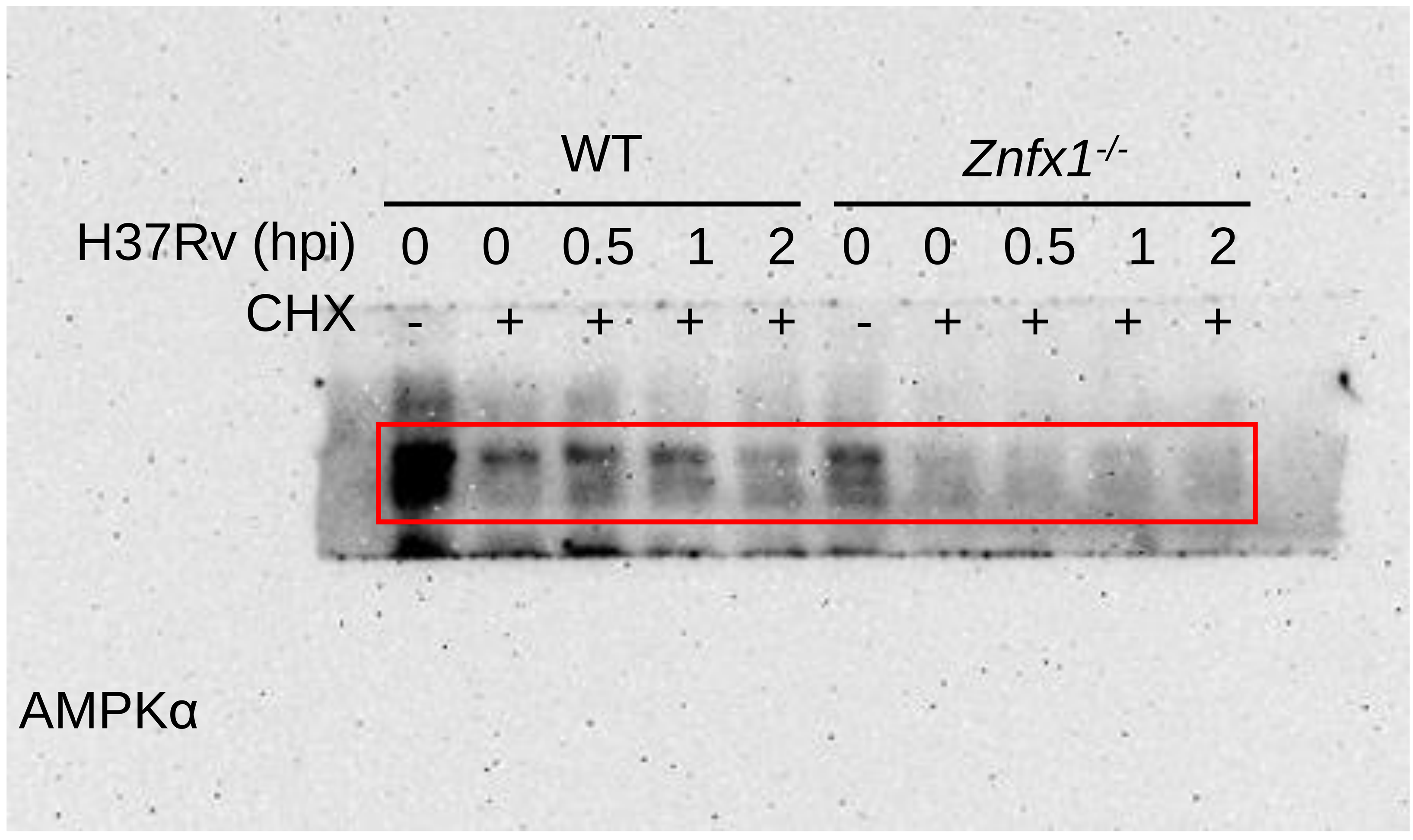

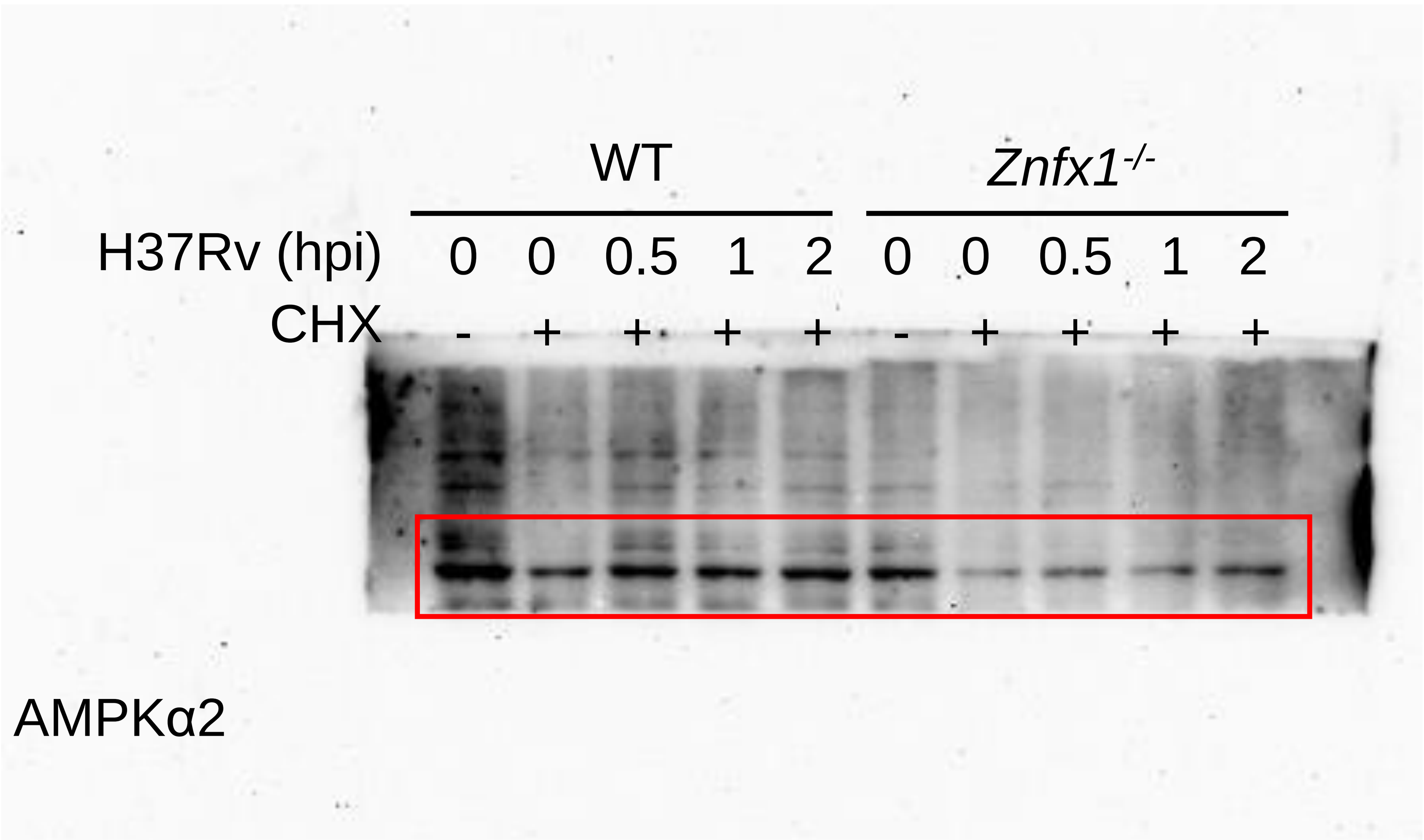

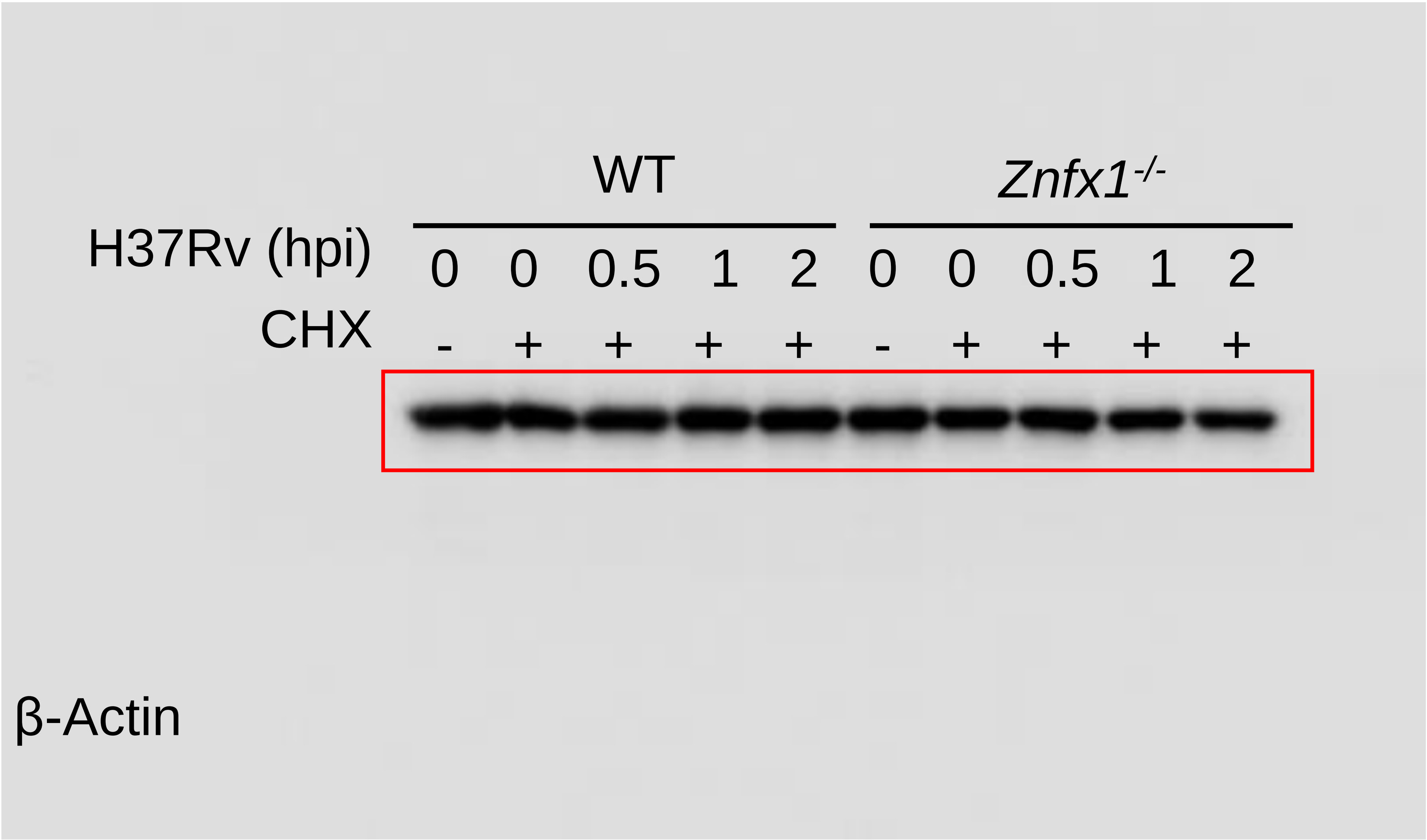

Full unedited gel for Supplementary Figure 7D

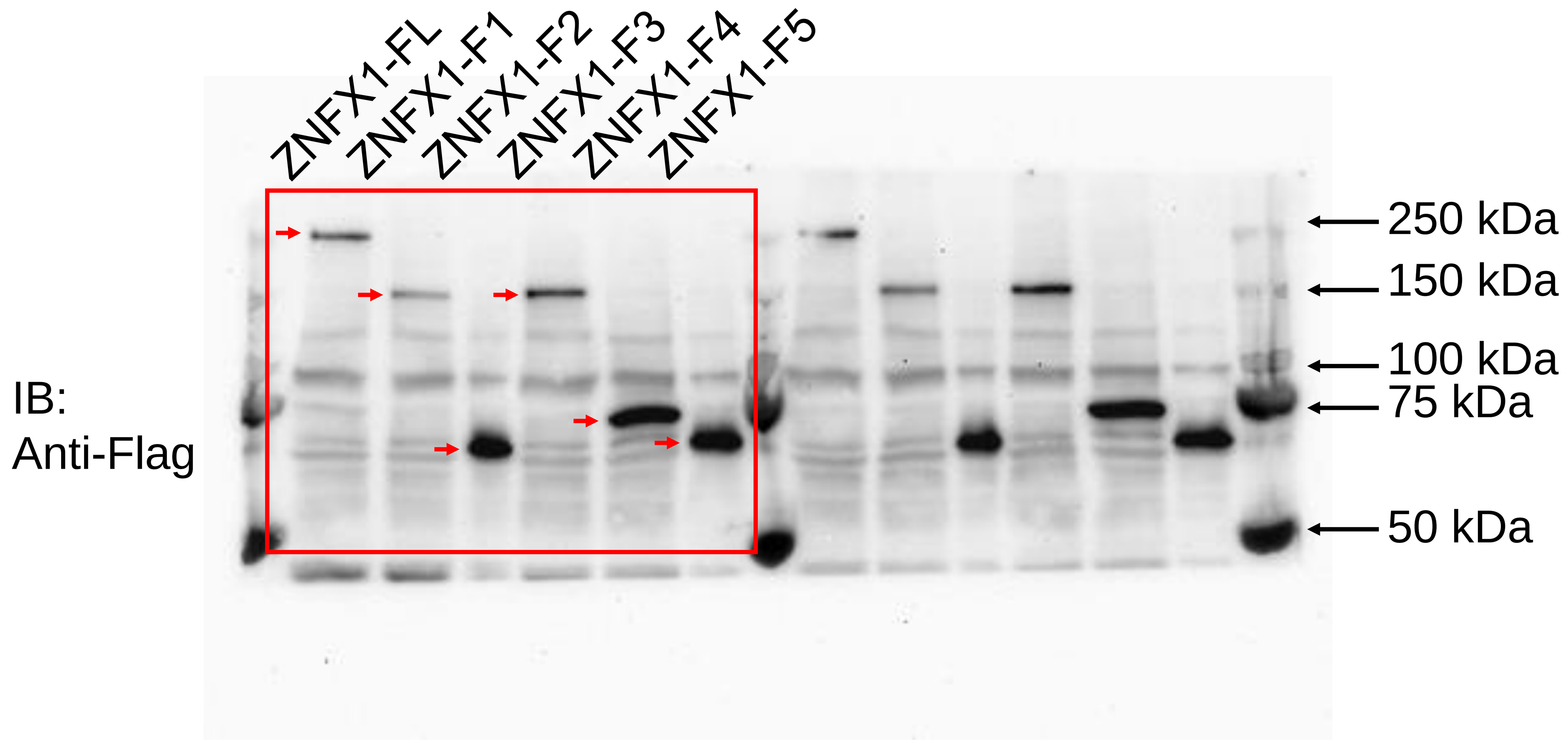

Supplement: Unedited blot and gel images [file jciinsight-9-171850-s083.pdf]
