# Supplementary material for: ZNFX1 promotes AMPK-mediated autophagy against Mycobacterium tuberculosis by stabilizing Prkaa2 mRNA
Source: JCI Insight. 2024 Jan 9;9(1):e171850. doi: 10.1172/jci.insight.171850 (PMC10906457; doi:10.1172/jci.insight.171850)
Supplement: Supplemental table 2 [file jciinsight-9-171850-s085.pdf]

Supplementary Table S2. siRNA and qRT-PCR primers

**siRNA**		
**SiRNA**	**Targeting sequence**	**Accession number**
si-h-ZNFX1_001	*GGAATATCGTGGAGTGTAA*	*NM_021035.3*
si-h-ZNFX1_002	*CCACCATGGTCCAATGACA*	
si-h-ZNFX1_003	*GCGAGAAGATTTCGTCAGA*	

**qRT-PCR primers**			
**Gene**	**Primer pairs**	**Amplicon**	**Accession number**
hm-Actin	*FP: 5'-TCAAGATCATTGCTCCTCCTGAG- 3'*<br>*RP: 5'-ACATCTGCTGGAAGGTGGACA- 3'*	*87 bp*	*NM_001101.5*
mZnfx1	*FP: 5'-GGATGGAGAATTGCCACCAAG- 3'*<br>*RP: 5'-CTTCGTCACTGGTATGCCCTT- 3'*	*188 bp*	*NM_001033196.2*
mIl1b	*FP: 5'-GAAATGCCACCTTTTGACAGTG- 3'*<br>*RP: 5'-TGGATGCTCTCATCAGGACAG- 3'*	*116 bp*	*NM_008361.4*
mIl6	*FP: 5'-TACCACTTCACAAGTCGGAGGC- 3'*<br>*RP: 5'-CTGCAAGTGCATCATCGTTGTTC- 3'*	*118 bp*	*NM_001314054.1*
mIl10	*FP: 5'-GCTCTTACTGACTGGCATGAG- 3'*<br>*RP: 5'-CGCAGCTCTAGGAGCATGTG- 3'*	*78 bp*	*NM_010548.2*
mIl12a	*FP: 5'-CAATCACGCTACCTCCTCTTTT- 3'*<br>*RP: 5'-CAGCAGTGCAGGAATAATGTTTC- 3'*	*181 bp*	*XM_006501044.5*
mIl12b	*FP: 5'-TGGTTTGCCATCGTTTTGCTG- 3'*<br>*RP: 5'-ACAGGTGAGGTTCACTGTTTCT- 3'*	*123 bp*	*NM_001303244.1*
mTnf	*FP: 5'-CCTGTAGCCCACGTCGTAG- 3'*<br>*RP: 5'-GGGAGTAGACAAGGTACAACCC- 3'*	*148 bp*	*NM_001278601.1*
mIfng	*FP: 5'-ACAGCAAGGCGAAAAAGGATG- 3'*<br>*RP: 5'-TGGTGGACCACTCGGATGA- 3'*	*106 bp*	*NM_008337.4*
mMx1	*FP: 5'-GACCATAGGGGTCTTGACCAA- 3'*<br>*RP: 5'-AGACTTGCTCTTTCTGAAAAGCC- 3'*	*182 bp*	*NM_010846.1*
mIrf1	*FP: 5'-ATGCCAATCACTCGAATGCG- 3'*<br>*RP: 5'-TTGTATCGGCCTGTGTGAATG- 3'*	*203 bp*	*NM_001159396.1*
mIfit1	*FP: 5'-CTGAGATGTCACTTCACATGGAA- 3'*<br>*RP: 5'-GTGCATCCCCAATGGGTTCT- 3'*	*117 bp*	*NM_008331.3*
mPrkaa1	*FP: 5'-GTCAAAGCCGACCCAATGATA- 3'*<br>*RP: 5'-CGTACACGCAAATAATAGGGGTT- 3'*	*100 bp*	*NM_001013367.3*
mPrkaa2	*FP: 5'-CAGGCCATAAAGTGGCAGTTA- 3'*<br>*RP: 5'-AAAAGTCTGTCGGAGTGCTGA- 3'*	*156 bp*	*NM_001356568.1*
mZc3h12c	*FP: 5'-CTCTGAGGGAAGCACGAGTTC- 3'*<br>*RP: 5'-GAGCGACATAGCTGTCTGTGA- 3'*	*102 bp*	*NM_001162921.2*
mZc3h12d	*FP: 5'-ACATCAAGGTTTTTGTCCCATCT- 3'*<br>*RP: 5'-GGTCATCGTAGCAGACCACTC- 3'*	*159 bp*	*NM_172785.3*
mZfp36	*FP: 5'-TCTCTGCCATCTACGAGAGCC- 3'*<br>*RP: 5'-CCAGTCAGGCGAGAGGTGA- 3'*	*80 bp*	*NM_011756.4*
mTrafd1	*FP: 5'-ATGGCCGAGTTTCGAGATGAC- 3'*	*115 bp*	*NM_001163470.1*

	RP: 5'-ACACACCAATGTTCCTTTGACAG- 3'		
mTnfaip3	*FP: 5'-ACCATGCACCGATACACGC- 3'*<br>*RP: 5'-AGCCACGAGCTTCCTGACT- 3'*	*159 bp*	*NM_001166402.1*
mHelz2	*FP: 5'-TGTAGGCGTCACTGGAATAGA- 3'*<br>*RP: 5'-ATCGGGGAAGGTTGTTCTTGA- 3'*	*91 bp*	*NM_183162.2*
mParp12	*FP: 5'-CAACCTGAGTGTGTTGAGGAC- 3'*<br>*RP: 5'-CACCTTTGTTGTAGTGTAGGCAT- 3'*	*122 bp*	*NM_172893.3*
mRnf114	*FP: 5'-CGGCAGATCGAGAGCATAGAG- 3'*<br>*RP: 5'-TGGCCTTTACACCTTCCATGA- 3'*	*127 bp*	*NM_030743.6*
mMorc3	*FP: 5'-AGGAGAGCGTTCCAAGACCA- 3'*<br>*RP: 5'-AGTTTCCGCTTCATGCTACTG- 3'*	*121 bp*	*NM_001045529.3*
mZfp36l2	*FP: 5'-AGCGGCTCCCAGATCAACT- 3'*<br>*RP: 5'-ACTTCTCGCCGTACTTGCAC- 3'*	*655 bp*	*NM_001001806.2*
mZfp36l1	*FP: 5'-GCTTTCGAGACCGCTCTTTCT- 3'*<br>*RP: 5'-TTGTCCCCGTACTTACAGGCA- 3'*	*148 bp*	*NM_007564.5*
mZdhhc20	*FP: 5'-GGAAAGACCGTTGTTTACCTTGT- 3'*<br>*RP: 5'-ACTCCTTCTCATAACGCTCCTTC- 3'*	*148 bp*	*NM_001360097.1*
mUhrf1	*FP: 5'-CCACACCGTGAACTCTCTGTC- 3'*<br>*RP: 5'-GGCGCACATCATAATCGAAGA- 3'*	*156 bp*	*NM_001111078.2*
hZNFX1	*FP: 5'-AAATCAGGCCAATAACCCACC- 3'*<br>*RP: 5'-GGCCCTAAATCTCTCTTCCCT- 3'*	*121 bp*	*NM_021035.3*
hPRKAA2	*FP: 5'-CGGCTCTTTCAGCAGATTCTGT- 3'*<br>*RP: 5'-ATCGGCTATCTTGGCATTCATG- 3'*	*120 bp*	*NM_006252.4*
hZC3H12C	*FP: 5'-CAGCAAAGTGGAGTCAAGTACA- 3'*<br>*RP: 5'-GCTGGACTTAACTGTGGGTCA- 3'*	*111 bp*	*NM_001411037.1*
hHELZ2	*FP: 5'-GGTGCATCTGTGTCGTTTCC- 3'*<br>*RP: 5'-CGTCGGTGAAGAACTCAGGG- 3'*	*201 bp*	*NM_001037335.2*

PCR primers for Mouse identification

**Gene**	**Primer pairs**	**Amplicon**
Znfx1-sg-F1	*5'-CAACCCTGTCTGTGTTATGGGCA- 3'*	*Wt: none*<br>*KO: ~327 bp*
Znfx1-sg-R1	*5'-GGTGAGAAATGGAGAGATGGCTCTA- 3'*	
Znfx1-sg-F1	*5'-CAACCCTGTCTGTGTTATGGGCA- 3'*	*Wt:397 bp*<br>*KO: none*
Znfx1-wt-R1	*5'-CCTCAGATGCCTTCCAGCATG- 3'*	
